# World Health Organization Estimates of the Global and Regional Disease Burden of 22 Foodborne Bacterial, Protozoal, and Viral Diseases, 2010: A Data Synthesis

**DOI:** 10.1371/journal.pmed.1001921

**Published:** 2015-12-03

**Authors:** Martyn D. Kirk, Sara M. Pires, Robert E. Black, Marisa Caipo, John A. Crump, Brecht Devleesschauwer, Dörte Döpfer, Aamir Fazil, Christa L. Fischer-Walker, Tine Hald, Aron J. Hall, Karen H. Keddy, Robin J. Lake, Claudio F. Lanata, Paul R. Torgerson, Arie H. Havelaar, Frederick J. Angulo

**Affiliations:** 1 The Australian National University, Canberra, Australia; 2 Danish Technical University, Copenhagen, Denmark; 3 Johns Hopkins University, Baltimore, Maryland, United States of America; 4 Food and Agriculture Organization, Rome, Italy; 5 Centre for International Health, University of Otago, Dunedin, New Zealand; 6 Ghent University, Merelbeke, Belgium; 7 Université catholique de Louvain, Brussels, Belgium; 8 Institute of Tropical Medicine, Antwerp, Belgium; 9 University of Wisconsin, Madison, Madison, Wisconsin, United States of America; 10 Public Health Agency of Canada, Guelph, Ontario, Canada; 11 Centers for Disease Control and Prevention, Atlanta, Georgia, United States of America; 12 Centre for Enteric Diseases, National Institute for Communicable Diseases, and Faculty of Health Sciences, University of Witwatersrand, Johannesburg, South Africa; 13 Institute of Environmental Science and Research, Christchurch, New Zealand; 14 Instituto de Investigación Nutricional, Lima, Peru; 15 US Naval Medical Research Unit No. 6, Callao, Peru; 16 University of Zürich, Zürich, Switzerland; 17 National Institute for Public Health and the Environment, Bilthoven, The Netherlands; 18 University of Florida, Gainesville, Gainesville, Florida, United States of America; 19 Utrecht University, Utrecht, The Netherlands; Mahidol-Oxford Tropical Medicine Research Unit, THAILAND

## Abstract

**Background:**

Foodborne diseases are important worldwide, resulting in considerable morbidity and mortality. To our knowledge, we present the first global and regional estimates of the disease burden of the most important foodborne bacterial, protozoal, and viral diseases.

**Methods and Findings:**

We synthesized data on the number of foodborne illnesses, sequelae, deaths, and Disability Adjusted Life Years (DALYs), for all diseases with sufficient data to support global and regional estimates, by age and region. The data sources included varied by pathogen and included systematic reviews, cohort studies, surveillance studies and other burden of disease assessments. We sought relevant data circa 2010, and included sources from 1990–2012. The number of studies per pathogen ranged from as few as 5 studies for bacterial intoxications through to 494 studies for diarrheal pathogens. To estimate mortality for *Mycobacterium bovis* infections and morbidity and mortality for invasive non-typhoidal *Salmonella enterica* infections, we excluded cases attributed to HIV infection. We excluded stillbirths in our estimates. We estimate that the 22 diseases included in our study resulted in two billion (95% uncertainty interval [UI] 1.5–2.9 billion) cases, over one million (95% UI 0.89–1.4 million) deaths, and 78.7 million (95% UI 65.0–97.7 million) DALYs in 2010. To estimate the burden due to contaminated food, we then applied proportions of infections that were estimated to be foodborne from a global expert elicitation. Waterborne transmission of disease was not included. We estimate that 29% (95% UI 23–36%) of cases caused by diseases in our study, or 582 million (95% UI 401–922 million), were transmitted by contaminated food, resulting in 25.2 million (95% UI 17.5–37.0 million) DALYs. Norovirus was the leading cause of foodborne illness causing 125 million (95% UI 70–251 million) cases, while *Campylobacter* spp. caused 96 million (95% UI 52–177 million) foodborne illnesses. Of all foodborne diseases, diarrheal and invasive infections due to non-typhoidal *S*. *enterica* infections resulted in the highest burden, causing 4.07 million (95% UI 2.49–6.27 million) DALYs. Regionally, DALYs per 100,000 population were highest in the African region followed by the South East Asian region. Considerable burden of foodborne disease is borne by children less than five years of age. Major limitations of our study include data gaps, particularly in middle- and high-mortality countries, and uncertainty around the proportion of diseases that were foodborne.

**Conclusions:**

Foodborne diseases result in a large disease burden, particularly in children. Although it is known that diarrheal diseases are a major burden in children, we have demonstrated for the first time the importance of contaminated food as a cause. There is a need to focus food safety interventions on preventing foodborne diseases, particularly in low- and middle-income settings.

## Abstract 2

In this data synthesis, Martyn Kirk and colleagues estimate the global and regional disease burden of 22 foodborne bacterial, protozoal and viral diseases.

## Editors' Summary

**Background:**

Foodborne diseases are responsible for a large burden of illness (morbidity) and death (mortality) in both resource-rich and resource-poor countries. More than 200 diseases can be transmitted to people through the ingestion of food contaminated with microorganisms (bacteria, viruses, and parasites) or with chemicals. Contamination of food can occur at any stage of food production—on farms where crops are grown and animals raised, in factories where food is processed, and during food storage and preparation in shops, restaurants and the home. Contamination can arise because of pollution of the water, soil or air or through poor food-handling practices such as failing to wash one’s hands before preparing food. Many foodborne diseases (for example, norovirus, *Escherichia coli*, and campylobacter infections) present with gastrointestinal symptoms—stomach cramps, diarrhea, and vomiting. However, some foodborne illnesses cause symptoms affecting other parts of the body and some have serious sequelae (abnormal bodily conditions or diseases arising from a pre-existing disease). For example, infection with some strains of *E*. *coli* can lead to kidney failure.

**Why Was This Study Done?:**

Accurate regional and global estimates of the disease burden of foodborne illnesses are needed to guide governmental and international efforts to improve food safety. However, estimates of the number of cases of foodborne illness, sequelae, deaths, and disability adjusted life years (a DALY represents the disease-related loss of one year of full health because of premature death or disability; DALYs provide a measure of the burden of a disease) are only available for a few countries. Consequently, in 2007, the World Health Organization (WHO) established the Foodborne Disease Burden Epidemiology Reference Group (FERG) to estimate the global and regional burden of disease attributable to foodborne illnesses. Here, researchers involved in one of the constituent task forces of FERG—the Enteric Diseases Task Force—undertake a data synthesis (the combination of information from many different sources) to provide global and regional estimates of the disease burden of several important foodborne bacterial, protozoal (parasitic), and viral diseases.

**What Did the Researchers Do and Find?:**

The researchers combined national estimates of foodborne diseases and data from systematic reviews (studies that identify all the research on a given topic using predefined criteria), national surveillance programs, and other sources to estimate the number of illnesses, sequelae, deaths and DALYs globally and regionally for 22 diseases with sufficient data to support such estimations. Together, these 17 bacterial infections, two viral infections, and three protozoal infections caused 2 billion cases of illness, more than 1 million deaths, and almost 80 million DALYs in 2010. Using information on the proportions of infections considered to be foodborne by expert panels, the researchers estimated that nearly a third of these cases of illness (582 million cases), resulting in 25 million DALYs, were transmitted by contaminated food. Notably, 38% of the cases of foodborne illness, 33% of deaths from these diseases, and 43% of the disease burden from contaminated food (11 million DALYs) occurred in children under 5 years old. The leading cause of foodborne illness was norovirus (125 million cases), closely followed by campylobacter (96 million); diarrheal and invasive infections caused by non-typhoidal *Salmonella enterica* infections caused the largest burden of disease (4.07 million DALYs). Finally, the burden of foodborne illness was highest in WHO’s African region.

**What Do These Findings Mean?:**

The lack of reliable data on the 22 illnesses considered in this analysis for many regions of the world, including some of the most populous regions, and uncertainty about the proportion of the cases of each illness that is foodborne may limit the accuracy of these findings. Nevertheless, these results provide new information about the regional and global disease burden caused by foodborne illnesses. In particular, these estimates reveal an unexpectedly high disease burden caused by foodborne illnesses among young children. Thus, although children under the age of 5 years represent only 9% of the global population, nearly half of the disease burden from contaminated food may occur in this age group. Overall, the findings of this study suggest that governments and international agencies should prioritize food safety to prevent foodborne illness, particularly among young children, and highlight the need to identify effective food hygiene interventions that can be implemented in low- and middle-income countries.

**Additional Information:**

This list of resources contains links that can be accessed when viewing the PDF on a device or via the online version of the article at http://dx.doi.org/10.1371/journal.pmed.1001921.

- The World Health Organization provides information about foodborne diseases, food safety and the estimation of the global burden of foodborne diseases (available in several languages)
- The US National Institute of Allergy and Infectious Diseases provides detailed information about several foodborne illnesses
- The US Centers for Disease Control and Prevention provides information about foodborne disease outbreaks in the US and elsewhere and information about food safety in the US
- The UK National Health Service Choices website provides information about food poisoning (another name for foodborne illness) and about food safety
- STOP Foodborne Illness STOP Foodborne Illness, a US non-profit public-health organization, provides personal stories about foodborne illness

## Introduction

Foodborne diseases are globally important, as they result in considerable morbidity, mortality, and economic costs [1,2]. Many different diseases, including those due to bacteria, viruses, parasites, chemicals, and prions, may be transmitted to humans by contaminated food [3]. Outbreaks and sporadic cases of foodborne disease are regular occurrences in all countries of the world. In recent decades, globalization of the food supply has also meant that pathogens causing foodborne diseases are rapidly transported across international borders [4]. Foodborne disease outbreaks have led to adverse impacts on trade and food security [5,6]. In response to foodborne diseases, national governments and international bodies have established elaborate systems to control and improve food safety [7].

Recognizing that contaminated food is an important cause of human disease, estimates of disease burden of the various foodborne diseases has been sought to enable advocacy for improved food safety and to assist governments to prioritize efforts for enhancing food safety. Although several countries have estimated the number of cases, sequelae, deaths, and Disability Adjusted Life Years (DALYs) resulting from foodborne diseases at the national level, most have not [8]. Furthermore, global and regional estimates of the burden of foodborne diseases have not been available [1,2]. In 2007, the World Health Organization (WHO) established the Foodborne Disease Burden Epidemiology Reference Group (FERG) to estimate the global and regional burden of disease attributable to food from all causes [9]. FERG consists of thematic task forces to estimate the human health burden of (1) enteric bacterial and viral infections, (2) parasitic infections, and (3) illnesses due to chemicals and toxins. The Director General of WHO nominated members of the FERG following an open call for applications to governments, in the scientific press, and through global networks [1]. The Enteric Diseases Task Force (EDTF) of the FERG comprised 14 experts in the epidemiology of viral, bacterial and parasitic infections transmitted by food, and was supported by various resource advisors who were expert in various aspects of infectious disease transmission.

## Methods

In this study, the EDTF estimates the global and regional disease health burden in 2010 resulting from the most common foodborne diseases. For a glossary of terms used in this paper see S1 Text.

FERG EDTF reviewed the epidemiology of all bacterial and viral diseases potentially transmitted by food and identified those for inclusion based on public health importance and data availability. We excluded enteroaggerative *Escherichia coli*, *Vibrio parahaemolyticus*, *V*. *vulnificus*, and *Yersinia* spp., which are infrequent causes of foodborne diseases, due to a lack of sufficient data for global estimation, ie–not commonly reported in systematic reviews of etiological agents of diarrhea [10]. After excluding these agents, we included 19 bacterial or viral diseases in our study. Of these 19 diseases, four are distinct manifestations of *Salmonella enterica* infection: invasive infections due to *S*. *enterica* serotype Typhi (*Salmonella* Typhi); invasive infections due to *S. enterica* serotype Paratyphi A (*Salmonella* Paratyphi A); invasive infections due to non-typhoidal *S*. *enterica* (iNTS); and diarrheal disease due to non-typhoidal *S*. *enterica* (NTS). We then determined that our approach for estimating the burden for diarrheal diseases could also be applied to protozoal diseases and included 3 protozoal diseases.

Diarrhea is a dominant feature for 14 of these diseases—ten caused by bacteria, three by protozoa, and one by a virus. One or more extra-intestinal manifestations including bacteremia, hepatitis, and meningitis are the dominant feature for the other eight diseases—seven caused by bacteria and one caused by a virus.

To identify data for estimation of incidence, mortality and sequelae for different agents, we conducted literature reviews to identify relevant studies, consulted with academics and expert committees, and evaluated systematic reviews that were conducted circa 2010. Where data sources were not available, the EDTF recommended that WHO commission systematic reviews into incidence and outcomes of specific agents, which were subsequently published in the peer-reviewed literature [11–22]. Where multiple data sources were available for a single agent, the EDTF made decisions to use the most contemporaneous and comprehensive sources, or incorporate both into the estimation process. In the following sections, we outline how we synthesized data in accordance with a documented framework reported elsewhere [23]. Table 1 summarizes our approach, while S2 Text provides a comprehensive description of the methods and distribution for all parameters used for estimating cases, sequelae, deaths and DALYs for each of the 22 diseases.

**Table 1:** Summary of enteric pathogens included and methods of estimating incidence of infection. For more information, see S2 Text.

Disease	Pathogens	Methods	Reference
Diarrheal Disease	*Campylobacter* spp., *Cryptosporidium* spp., *Entamoeba histolytica*, enterotoxigenic *E*. *coli* (ETEC), enteropathogenic *E*. *coli* (EPEC), *Giardia lamblia*, norovirus, nontyphoidal *Salmonella enterica*, *Shigella* spp	Synthesis of seven national studies into foodborne disease incidence for low-mortality countries in Region A combined with systematic reviews for middle- and high-mortality countries that was derived from 136 outpatient and community studies and 254 inpatient studies among children <5 years of age, and 97 inpatient, outpatient, and community studies among persons ≥5 years of age	[11]
Diarrheal Disease	*Vibrio cholerae* (cholera)	Data synthesis of WHO cholera reports and publicly available data sources.	[24]
Diarrheal Disease	Shiga-toxin producing *E*. *coli*	Systematic review of 16 published studies	[19]
Intoxications	*Bacillus cereus*, *Clostridium perfringens*, *Staphylococcus aureus*	Estimation from seven national studies in low-mortality sub-region A countries only	[11]
Intoxications	*Clostridium botulinum*	Estimation from five national studies in 55 EUR and AMR sub-region A countries only*	See S2 Text
Invasive enteric disease	Hepatitis A virus	Conversion of mortality data from Global Burden of Disease 2010 for hepatitis A to incidence	[25]
Invasive enteric disease	Typhoid, Paratyphoid fevers	Data from Global Burden of Disease 2010	[26]
Invasive enteric disease	Invasive non-typhoidal *Salmonella enterica* (iNTS)	Systematic review of 10 published studies of incidence of invasive infections	[22]
Invasive enteric disease	*Mycobacterium bovis*	Systematic review of 140 data sources combined with incidence estimates for human TB infections.	[16,17]
Invasive enteric disease	*Brucella* spp.	Systematic review of 29 published studies of incidence combined with information from countries free of *Brucella* in livestock.	[15]
Invasive enteric disease	*Listeria* spp.	Systematic review of 87 published studies, including 43 containing data on incidence	[18]

* EUR— European Region; AMR— Region of the Americas

### Estimating Cases, Sequelae, and Deaths for Diarrheal Diseases

For diarrheal diseases caused by *Campylobacter* spp., *Cryptosporidium* spp., *Entamoeba histolytica*, enterotoxigenic *E*. *coli* (ETEC), enteropathogenic *E*. *coli* (EPEC), *Giardia lamblia*, norovirus, NTS, and *Shigella* spp., national estimates of foodborne diseases were only available from a limited number of countries. We therefore used two approaches, documented in Pires *et al*. [11], depending on the level of development of the country. The first approach, based on national estimates of the incidence of foodborne diseases from seven studies published between 2005–2014, was applied to the 61 countries in low-mortality (European Region (EUR) and other subregion A [27]) countries [3,28–35]. For countries with national estimates of incidence and mortality, we used these data. We used the median and associated uncertainty intervals for diarrheal diseases for the subregion to estimate incidence and mortality of diarrheal diseases for other countries within the subregion without national data [10].

The second approach was applied to the remaining 133 countries not in EUR or other subregion A categories (see S1 Text for a country list by subregion). For this approach, we modified the WHO Child Health Epidemiology Reference Group (CHERG) method to estimate diarrheal incidence and mortality for all age groups [10]. First, we estimated the overall incidence of diarrhea from all causes (i.e., “envelope” of diarrheal incidence) for 2010 by combining estimates of diarrheal incidence for children <5 years of age and persons ≥5 years of age [13,36]. We used the overall diarrheal mortality (i.e., envelope for diarrheal deaths) derived by WHO [37] for 2010. We derived an etiological proportion for each disease by region from systematic reviews of stool sample isolation or detection proportions from inpatient, outpatient, and community-based studies of persons with diarrhea. We followed the CHERG standard approach because there is limited information on pathogens among people who have died, and assumed that the distribution of pathogens observed among inpatients hospitalized with severe diarrhea represented the pathogen prevalence among diarrheal deaths [10]. To derive etiological proportions for children <5 years of age, we assumed that the distribution of pathogens in outpatient and community studies represented the pathogen prevalence among diarrheal episodes for those who did not die. We made the same assumption for persons ≥5 years of age but due to sparseness of data also included inpatient studies. For more details of methods, see Pires *et al*. [11]. For some pathogens we assumed that different etiological agents, such as *Shigella* spp., NTS and *Campylobacter* spp., had similar clinical profiles (S2 Text).

Estimates for cholera were based on the incidence among populations at risk for cholera in endemic and non-endemic countries [24]. The case fatality ratio (CFR) for cholera was 1% in Western Pacific Region (WPR) subregion B, 1% in South-East Asian Region (SEAR) subregion B (except 1.5% in Bangladesh), 1.3% in Eastern Mediterranean Region (EMR) subregion B, 3% in SEAR subregion D, 3.2% in EMR subregion D, and 3.8% in African Region (AFR) [24]. For all other countries, we assumed cholera occurred only among international travellers and did not result in deaths. In this instance, we applied the median incidence from non-endemic countries with available data for cholera.

Shiga-toxin producing *E*. *coli* (STEC) infection incidence and mortality were based on a systematic review described elsewhere [19]. Sequelae, more common among O157 infections, were hemolytic uremic syndrome (HUS) and end stage renal disease (ESRD). Based on review, we estimated 0.8% of O157 infections and 0.03% of infections caused by other serotypes result in HUS, and 3% of HUS cases result in ESRD. We estimated that the CFR for HUS was 3.7%; for ESRD the CFR was 20% in the 35 subregion A countries and 100% in other countries.

We used data on the incidence and mortality of foodborne intoxications caused by *Bacillus cereus*, *Clostridium perfringens*, and *Staphylococcus aureus* from national studies conducted in low-mortality countries. We applied the median incidence from national studies to the 61 countries in EUR and other subregion A countries. We did not attempt to estimate burden due to these three foodborne intoxications in high- and middle-mortality countries due to the absence of data on diseases caused by these pathogens in these countries. The median CFR from national studies was 0.003% for *C*. *perfringens* and 0.0025% for *S*. *aureus*; there were no *B*. *cereus* deaths. We considered that 31% of Guillain-Barré Syndrome (GBS) cases globally were associated with antecedent *Campylobacter* infection and that the CFR for GBS was 4.1% [38,39].

### Estimating Cases, Sequelae, and Deaths of Extra-Intestinal Diseases

For diseases caused by hepatitis A virus, *Brucella* spp., *Listeria monocytogenes*, *Mycobacterium bovis*, iNTS, *Salmonella* Paratyphi A, and *Salmonella* Typhi we used a variety of approaches depending on availability of data.

We used Institute of Health Metrics and Evaluation (IHME) Global Burden of Disease (GBD) 2010 data to estimate the burden of disease for typhoid, paratyphoid, and hepatitis A [25]. IHME provided country-specific age-standardized prevalence data of typhoid and paratyphoid fever. These data were converted to incidence by dividing by duration, and partitioned into typhoid and paratyphoid assuming a 1.0 to 0.23 ratio [40]. Country-specific hepatitis A mortality data, stratified by age and sex, were converted to incidence assuming a CFR of 0.2%.

Rates of iNTS are highly correlated with HIV prevalence and malaria risk [22]. To estimate iNTS incidence globally, we used age-specific estimates of incidence from a systematic review [22] to construct a random effect log linear model using covariates of country specific HIV and malaria deaths, and the log of Gross Domestic Product. As data were sparse, we predicted incidence for all ages, which was converted to age-specific incidence based on age profiles for iNTS cases in low and high incidence settings [22]. From this, we predicted iNTS incidence among persons not infected with HIV [41,42]. To estimate deaths, we assumed that the CFR for iNTS in non-HIV infected individuals was a uniform distribution with a range 5–20% in sub-region B-E countries and range 3.9–6.6% in sub-region A countries [43].

Estimates for *M*. *bovis* infections were based on a systematic review where the proportion of human tuberculosis (TB) infections due to *M*. *bovis* ranged from 0.3% in Region of the Americas (AMR) to 2.8% in AFR [17]. We identified 51 countries that were free from *M*. *bovis* in cattle based on European Union certification and the World Organization for Animal Health [44]. All countries in a region except those free from *M*. *bovis* in cattle were assumed to have the same proportion of human TB infections due to *M*. *bovis*. To account for internationally acquired infections, all countries free of *M*. *bovis* in cattle were assigned the lowest observed proportion of human TB infections due to *M*. *bovis* (0.3%). To derive estimates of human *M*. *bovis* incidence, we multiplied WHO country-specific human TB incidence by the estimate of the proportion of human TB infections that were due to *M*. *bovis* [45]. To estimate mortality associated with *M*. *bovis* that accounted for HIV co-morbidity we used estimates of mortality due to human TB in HIV negative persons from WHO. We adjusted mortality data by assuming that the CFR for *M*. *bovis* was 20% lower than human TB, as *M*. *bovis* infections are more likely to be extrapulmonary [16].

To estimate the incidence and mortality for brucellosis we updated a systematic review and included additional data on 32 countries that were considered *Brucella-*free in livestock (free of *B*. *arbortus* in cattle and *B*. *melitensis* in sheep and goats) [15]. We imputed incidence data to countries without estimates using a Bayesian log-normal random effects model, except for countries that were *Brucella-*free in livestock [23]. To account for internationally acquired infections, all countries that were *Brucella-*free in livestock were assigned the median incidence of human brucellosis reported from these countries. The CFR for brucellosis was 0.05%, and 40% of cases resulted in chronic infections and 10% of cases in males resulted in orchitis [14].

We estimated the incidence and mortality for listeriosis using a systematic review that is described elsewhere [18]. In accordance with standard burden of disease practice, we excluded stillbirths, in our baseline burden estimates. The CFR was 14.9% for perinatal cases and 25.9% for other cases.

Incidence and mortality data for botulism were only available from countries in Europe and North America. We limited our estimation to the 55 countries in EUR and AMR subregion A, which was based on the median incidence derived from countries with national estimates of botulism. We estimated that 35% of botulism cases were severe and that the CFR of severe botulism was 15%.

### Estimation of Foodborne Proportion

We assumed that all infections from *L*. *monocytogenes*, *M*. *bovis*, and from the four foodborne intoxications (*B*. *cereus*, *C*. *botulinum*, *C*. *perfringens*, and *S*. *aureus*) were foodborne. To estimate the proportion of the other enteric diseases that were transmitted by contaminated food, we relied upon results of a FERG structured expert elicitation [46]. In this expert elicitation, foodborne and waterborne were considered separate transmission pathways. Proportions of brucellosis and hepatitis A infections and intestinal protozoa acquired from contaminated food were estimated using global panels of experts. Foodborne proportions for the remaining enteric diseases were estimated using a panel of experts for each region separately, although several experts were serving on more than one panel.

### Disability Weights

Disability weights for each clinical outcome for the enteric diseases were taken from the GBD 2010 study [47]. Disability weights for listeriosis meningitis and neurological sequelae were derived from GBD 2010 disability weights using a multiplicative methodology and expert judgment. Disability weights for individual conditions are specified in S2 Text.

### Data Analysis

The FERG approach to computing DALYs from the estimated cases, sequelae, deaths, and other parameters described in this paper is described in more detail elsewhere, including the modelling of uncertainty intervals incorporating (where relevant) uncertainty of estimated foodborne proportions [23]. Estimates were produced using the 2012 revisions for United Nations country-level population data for 2010 [48]. Calculations followed disease-specific models defined by incidence and probability parameters, each with a distribution [23]. Uncertainty around input parameters were propagated using Monte Carlo simulations; 10,000 values were sampled from each input parameter to calculate 10,000 estimates of cases, deaths or DALYs. The 2.5th and 97.5th percentile of each set of the 10,000 estimates yielded a 95% Uncertainty Interval (UI). Further details of analyses and modelling of DALYs and components described in this paper are in S2 Text.

## Results

### Cases

We estimated that the 22 diseases in our study caused 2.0 billion (95% UI 1.5–3.0 billion) illnesses in 2010, 39% (95% UI 26–53%) in children <5 years of age. Among the 1.9 billion (95% UI 1.4–2.8 billion) cases of diarrheal diseases, norovirus was responsible for 684 million (95% UI 491–1,112 million) illnesses; the largest number of cases for any pathogen (Table 2). The pathogens resulting in the next largest number of cases were ETEC, *Shigella* spp., *G*. *lamblia*, *Campylobacter* spp. and NTS. *Campylobacter* spp. cases included 31,700 (95% UI 25,400–40,200) GBS cases. There were also 2.48 million (95% UI 1.69–5.38 million) STEC cases which included 3,330 (95% UI 2,160–6,550) with HUS and 200 (95% UI 15–760) with ESRD. Among the extra-intestinal diseases, the pathogens resulting in the most infections were hepatitis A virus, *Salmonella* Typhi and *Salmonella* Paratyphi A. *Brucella* spp. resulted in 0.83 million (95% UI 0.34–19.6 million) illnesses, which included almost 333,000 (95% UI 135,000–7,820,000) chronic infections and 83,300 (95% UI 33,800–1,960,000) episodes of orchitis. *L*. *monocytogenes* resulted in 14,200 (95% UI 6,100–91,200) illnesses which included 7830 (95% UI 3,400–50,500) cases of septicaemia, 3,920 (95% UI 1,650–24,900) cases of meningitis, and 666 (95% UI 207–4,710) cases with neurological sequelae.

**Table 2:** Median number of foodborne illnesses, deaths, and Disability Adjusted Life Years (DALYs), with 95% uncertainty intervals, 2010.

PATHOGEN	ILLNESSES (95% UI)	DEATHS (95% UI)	DALYs (95% UI)	PROPORTION FOODBORNE (95% UI)	FOODBORNE ILLNESSES (95% UI)	FOODBORNE DEATHS (95% UI)	FOODBORNE DALYS (95% UI)
**Diarrheal Disease**	**1,912,159,038 (1,413,002,730–2,849,323,016)**	**715,196 (603,325–846,397)**	**55,139,959 (46,746,114–65,120,623)**	**0.29 (0.22–0.36)**	**548,285,159 (369,733,377–888,360,956)**	**199,892 (136,903–286,616)**	**15,780,400 (11,043,288–22,251,264)**
*Campylobacter* spp.*	166,175,078 (92,227,873–300,877,905)	37,604 (27,738–55,101)	3,733,822 (2,857,037–5,273,652)	0.58 (0.44–0.69)	95,613,970 (51,731,379–177,239,714)	21,374 (14,604–32,584)	2,141,926 (1,535,985–3,137,980)
*Cryptosporidium* spp.	64,003,709 (43,049,455–104,679,951)	27,553 (18,532–44,654)	2,159,331 (1,392,438–3,686,925)	0.13 (0.07–0.24)	8,584,805 (3,897,252–18,531,196)	3,759 (1,520–9,115)	296,156 (119,456–724,660)
*Entamoeba histolytica*	103,943,952 (47,018,659–210,632,459)	5,450 (2,194–17,127)	515,904 (222,446–1,552,466)	0.28 (0.14–0.44)	28,023,571 (10,261,254–68,567,590)	1,470 (453–5,554)	138,863 (47,339–503,775)
Enteropathogenic *E*. *coli*	81,082,327 (40,716,656–171,419,480)	122,760 (97,115–154,869)	9,717,390 (7,602,047–12,387,029)	0.30 (0.17–0.48)	23,797,284 (10,750,919–62,931,604)	37,077 (19,957–61,262)	2,938,407 (1,587,757–4,865,590)
Enterotoxigenic *E*. *coli*	240,886,759 (160,890,532–377,471,599)	73,857 (53,851–103,026)	5,887,541 (4,190,610–8,407,186)	0.36 (0.24–0.50)	86,502,735 (49,136,952–151,776,173)	26,170 (14,887–43,523)	2,084,229 (1,190,704–3,494,201)
*Giardia* spp.	183,842,615 (130,018,020–262,838,002)	0 (0–0)	171,100 (115,777–257,315)	0.15 (0.08–0.27)	28,236,123 (12,945,655–56,996,454)	0 (0–0)	26,270 (11,462–53,577)
Norovirus	684,850,131 (490,930,402–1,122,947,359)	212,489 (160,595–278,420)	15,105,714 (11,649,794–19,460,578)	0.18 (0.11–0.30)	124,803,946 (70,311,254–251,352,877)	34,929 (15,916–79,620)	2,496,078 (1,175,658–5,511,092)
*Salmonella enterica*, non-typhoidal	153,097,991 (64,733,607–382,208,079)	56,969 (43,272–88,129)	4,377,930 (3,242,020–7,175,522)	0.52 (0.35–0.67)	78,439,785 (31,579,011–210,875,866)	28,693 (17,070–49,768)	2,183,146 (1,314,295–3,981,424)
*Shigella* spp.	190,849,501 (97,832,995–363,915,689)	65,796 (46,317–97,036)	5,407,736 (3,771,300–8,107,456)	0.27 (0.13–0.44)	51,014,050 (20,405,214–118,927,631)	15,156 (6,839–30,072)	1,237,103 (554,204–2,520,126)
Shiga toxin-producing *E*. *coli*	2,481,511 (1,594,572–5,376,503)	269 (111–814)	26,827 (12,089–72,204)	0.48 (0.33–0.60)	1,176,854 (754,108–2,523,007)	128 (55–374)	12,953 (5,951–33,664)
*Vibrio cholerae*	3,183,394 (2,211,329–4,146,250)	105,170 (78,671–126,058)	7,347,635 (5,496,431–8,804,408)	0.24 (0.10–0.46)	763,451 (310,910–1,567,682)	24,649 (10,304–50,042)	1,722,312 (720,029–3,491,997)
**Intoxications**	**5,409,083 (2,187,762–12,929,293)**	**175 (70–407)**	**9,905 (3,993–23,527)**	**1.00**	**5,409,083 (2,187,762–12,929,293)**	**175 (70–407)**	**9,905 (3,993–23,527)**
*Bacillus cereus* **	256,775 (43,875–807,547)	0 (0–0)	45 (7–171)	1.00	256,775 (43,875–807,547)	0 (0–0)	45 (7–171)
*Clostridium botulinum* ***	475 (183–990)	24 (7–65)	1,036 (299–2,805)	1.00	475 (183–990)	24 (7–65)	1,036 (299–2,805)
*Clostridium perfringens* **	3,998,164 (837,262–11,529,642)	120 (25–351)	6,963 (1,423–20,493)	1.00	3,998,164 (837,262–11,529,642)	120 (25–351)	6,963 (1,423–20,493)
*Staphylococcus aureus* **	1,073,339 (658,463–1,639,524)	25 (10–55)	1,575 (702–3,244)	1.00	1,073,339 (658,463–1,639,524)	25 (10–55)	1,575 (702–3,244)
**Invasive enteric diseases**	**77,929,723 (36,606,712–149,676,316)**	**371,002 (218,593–631,271)**	**23,070,841 (13,388,154–39,912,033)**	**0.34 (0.17–0.52)**	**25,569,838 (10,019,370–58,282,758)**	**146,981 (81,052–274,835)**	**9,107,557 (4,891,985–17,483,327)**
*Brucella* spp.	832,633 (337,929–19,560,440)	4,145 (1,557–95,894)	264,073 (100,540–6,187,148)	0.47 (0.30–0.61)	393,239 (143,815–9,099,394)	1,957 (661–45,545)	124,884 (43,153–2,910,416)
Hepatitis A	46,864,406 (14,417,704–111,771,902)	93,961 (29,602–221,677)	4,580,758 (1,599,296–10,408,164)	0.30 (0.14–0.49)	13,709,836 (3,630,847–38,524,946)	27,731 (7,169–77,320)	1,353,767 (383,684–3,672,726)
*Listeria monocytogenes*	14,169 (6,112–91,175)	3,175 (1,339–20,428)	118,340 (49,634–754,680)	1.00	14,169 (6,112–91,175)	3,175 (1,339–20,428)	118,340 (49,634–754,680)
*Mycobacterium bovis*	121,268 (99,852–150,239)	10,545 (7,894–14,472)	607,775 (458,364–826,115)	1.00	121,268 (99,852–150,239)	10,545 (7,894–14,472)	607,775 (458,364–826,115)
*Salmonella enterica*, invasive non-typhoidal	596,824 (596,824–596,824)	63,312 (38,986–94,193)	3,895,547 (2,401,034–5,790,874)	0.48 (0.28–0.64)	284,972 (167,455–384,321)	29,391 (14,948–50,463)	1,794,575 (886,443–3,107,172)
*Salmonella enterica* Paratyphi A	4,826,477 (1,782,796–10,323,273)	33,325 (12,309–71,278)	2,367,164 (875,236–5,066,375)	0.37 (0.19–0.58)	1,741,120 (536,650–4,310,983)	12,069 (3,784–29,521)	855,730 (268,879–2,100,120)
*Salmonella enterica* Typhi	20,984,683 (7,751,285–44,883,794)	144,890 (53,519–309,903)	10,292,017 (3,805,373–22,027,716)	0.37 (0.19–0.58)	7,570,087 (2,333,263–18,743,406)	52,472 (16,454–128,350)	3,720,565 (1,169,040–9,130,956)
**TOTAL**	**2,000,626,631 (1,494,986,030–2,942,534,533)**	**1,092,584 (892,999–1,374,238)**	**78,730,084 (64,963,913–97,740,062)**	**0.29 (0.23–0.36)**	**581,902,722 (400,741,151–922,031,380)**	**350,686 (240,030–524,042)**	**25,175,035 (17,547,264–37,021,003)**

* Includes Guillain-Barré Syndrome cases and deaths.

** 61 EUR and other subregion A (low mortality) countries only.

*** EUR and subregion A (low mortality) countries only, excluding WPR A countries.

Overall, 29% (95% UI 23–36%) of all 22 diseases were estimated to be transmitted by contaminated food equating to 582 million (95% UI 400–922 million) foodborne cases in 2010; 38% (95% UI 24–53%) in children <5 years of age. The pathogens resulting in the most foodborne cases were norovirus, *Campylobacter* spp., ETEC, NTS, and *Shigella* spp. A high proportion of foodborne infections caused by *V*. *cholerae*, *Salmonella* Typhi, and *Salmonella* Paratyphi A occurred in the African region (Table 3). A high proportion of foodborne infections caused by EPEC, *Cryptosporidium* spp., and *Campylobacter* spp. occurred among children <5 years of age (Table 4). Among the 11 diarrheal diseases, the rate ratio of foodborne cases occurring among children <5 years of age compared to those ≥5 years of age was 6.44 (95%UI 3.15–12.46).

**Table 3:** Median rates of foodborne illnesses, deaths and Disability Adjusted Life Years (DALYs) per 100,000 persons, by region, with 95% uncertainty intervals, 2010.

PATHOGEN*	African Region (AFR)			Region of the Americas (AMR)			Eastern Mediterranean Region (EMR)		
	ILLNESSES (95% UI)	DEATHS (95% UI)	DALYs (95% UI)	ILLNESSES (95% UI)	DEATHS (95% UI)	DALYs (95% UI)	ILLNESSES (95% UI)	DEATHS (95% UI)	DALYs (95% UI)
**Diarrheal Disease**	9,830 (3,969–21,567)	9 (5–14)	687 (369–1,106)	7,900 (4,497–13,850)	0.5 (0.3–0.7)	44 (30–63)	16,387 (7,729–34,176)	4 (2–6)	354 (218–544)
*Campylobacter* spp.**	2,221 (335–8,482)	0.8 (0.4–1)	70 (41–112)	1,389 (490–3,207)	0.07 (0.04–0.1)	13 (8–18)	1,873 (488–5,608)	1 (0.6–1)	90 (56–130)
*Cryptosporidium* spp.	205 (35–813)	0.2 (0.04–0.4)	13 (3–37)	114 (32–355)	0.007 (0.002–0.02)	0.6 (0.2–2)	346 (52–1,287)	0.04 (0.004–0.2)	4 (0.4–20)
*Entamoeba histolytica*	796 (98–3,868)	0.05 (0.009–0.4)	5 (0.9–39)	212 (16–1,209)	0.001 (0–0.009)	0.3 (0.03–1)	737 (79–3,110)	0.02 (0.002–0.2)	2 (0.3–14)
Enteropathogenic *E*. *coli*	454 (125–1,215)	2 (0.6–3)	140 (50–282)	189 (35–730)	0.06 (0.01–0.1)	5 (1–12)	430 (116–1,222)	0.7 (0.2–2)	57 (18–131)
Enterotoxigenic *E*. *coli*	982 (312–2,480)	1 (0.6–3)	109 (46–216)	1,281 (299–3,295)	0.05 (0.01–0.1)	5 (1–12)	4,971 (1,685–10,849)	0.4 (0.1–1)	35 (11–89)
*Giardia* spp.	809 (172–2,574)	0 (0–0)	0.8 (0.2–3)	309 (62–1,249)	0 (0–0)	0.3 (0.05–1)	670 (133–2,193)	0 (0–0)	0.6 (0.1–2)
Norovirus	1,749 (491–5,060)	1 (0.3–3)	81 (24–185)	2,491 (898–6,186)	0.1 (0.04–0.3)	9 (3–23)	2,796 (744–7,376)	0.4 (0.1–1)	33 (9–76)
*Salmonella enterica*, non-typhoidal	896 (175–2,994)	1 (0.5–2)	89 (42–147)	1,002 (378–1,990)	0.1 (0.06–0.2)	7 (4–12)	1,610 (147–14,052)	0.6 (0.3–1)	54 (26–87)
*Shigella* spp.	523 (45–2,865)	0.5 (0.1–2)	43 (8–124)	278 (35–1,443)	0.02 (0.003–0.05)	1 (0.3–5)	627 (55–4,648)	0.4 (0.07–1)	38 (6–117)
Shiga toxin-producing *E*. *coli*	5 (2–9)	0 (0–0.002)	0.05 (0.02–0.1)	16 (9–30)	0.004 (0.001–0.01)	0.3 (0.1–0.9)	65 (37–97)	0.002 (0–0.004)	0.2 (0.1–0.5)
*Vibrio cholerae*	43 (13–101)	2 (0.5–4)	1 12 (35–252)	0.02 (0.008–0.05)	0 (0–0)	0 (0–0)	9 (0.4–28)	0.3 (0.01–1)	20 (0.7–69)
**Invasive enteric diseases**	**425 (156–976)**	**5 (3–8)**	**307 (160–508)**	**31 (11–81)**	**0.3 (0.2–0.6)**	**16 (8–35)**	**394 (80–1,056)**	**2 (0.7–4)**	**108 (41–250)**
*Brucella* spp.	3 (0.4–110)	0.02 (0.002–0.5)	1 (0.1–34)	3 (1–37)	0.01 (0.005–0.2)	0.9 (0.3–12)	33 (10–187)	0.2 (0.05–0.9)	11 (3–60)
Hepatitis A	232 (60–643)	0.5 (0.1–1)	23 (7–60)	12 (3–33)	0.02 (0.006–0.07)	1 (0.3–3)	237 (17–772)	0.5 (0.04–2)	23 (2–74)
*Listeria monocytogenes*	0.1 (0–2)	0.03 (0–0.6)	1 (0–21)	0.3 (0.1–1)	0.07 (0.03–0.3)	3 (1–11)	0.1 (0–2)	0.03 (0–0.6)	1 (0–21)
*Mycobacterium bovis*	7 (4–9)	0.5 (0.3–0.7)	30 (19–42)	0.1 (0.05–0.2)	0.007 (0.003–0.01)	0.4 (0.2–0.8)	1 (0.8–2)	0.2 (0.08–0.3)	9 (5–18)
*Salmonella* enterica., invasive non-typhoidal	25 (12–37)	3 (1–5)	169 (71–306)	0.7 (0.4–0.9)	0.06 (0.03–0.1)	3 (1–5)	1 (0.7–2)	0.1 (0.06–0.3)	8 (3–14)
*Salmonella enterica* Paratyphi A	25 (5–73)	0.2 (0.04–0.5)	12 (3–36)	2 (0.4–7)	0.02 (0.003–0.05)	1 (0.2–4)	17 (2–55)	0.1 (0.01–0.4)	9 (1–28)
*Salmonella enterica* Typhi	108 (24–317)	0.7 (0.2–2)	53 (12–155)	10 (2–32)	0.07 (0.01–0.2)	5 (0.9–16)	73 (9–240)	0.5 (0.06–2)	37 (5–122)
**TOTAL**	**10,304 (4,279–22,108)**	**14 (8–21)**	**1,001 (562–1,543)**	**7,937 (4,515–13,899)**	**0.8 (0.5–1)**	**61 (40–93)**	**16,865 (8,051–34,712)**	**6 (4–9)**	**470 (286–728)**

PATHOGEN*	European Region (EUR)			South-East Asian Region (SEAR)			Western Pacific Region (WPR)			GLOBAL		
	ILLNESSES (95% UI)	DEATHS (95% UI)	DALYs (95% UI)	ILLNESSES (95% UI)	DEATHS (95% UI)	DALYs (95% UI)	ILLNESSES (95% UI)	DEATHS (95% UI)	DALYs (95% UI)	ILLNESSES (95% UI)	DEATHS (95% UI)	DALYs (95% UI)
**Diarrheal Disease**	2,483 (1,439–4,136)	0.3 (0.2–0.4)	23 (16–30)	7,074 (2,570–19,537)	5 (2–9)	363 (177–649)	6,302 (2,501–17,289)	0.3 (0.1–0.5)	33 (17–54)	7,968 (5,373–12,911)	3 (2–4)	229 (160–323)
*Campylobacter* spp.**	522 (363–687)	0.05 (0.03–0.09)	9 (6–13)	1,152 (200–3,372)	0.4 (0.1–0.9)	33 (9–83)	876 (359–3,855)	0.04 (0.02–0.1)	10 (4–17)	1,390 (752–2,576)	0.3 (0.2–0.5)	31 (22–46)
*Cryptosporidium* spp.	21 (4–70)	0.003 (0–0.009)	0.2 (0.03–0.6)	78 (10–474)	0.09 (0.01–0.4)	6 (0.9–29)	32 (2–170)	0.003 (0–0.03)	0.3 (0.02–3)	125 (57–269)	0.05 (0.02–0.1)	4 (2–11)
*Entamoeba histolytica*	0 (0–0)	0 (0–0)	0 (0–0)	256 (27–1,188)	0.03 (0.004–0.2)	3 (0.3–17)	229 (0–1,598)	0.001 (0–0.003)	0.3 (0–1)	407 (149–997)	0.02 (0.007–0.08)	2 (0.7–7)
Enteropathogenic *E*. *coli*	8 (3–16)	0 (0–0)	0.005 (0.002–0.01)	594 (62–2,775)	0.9 (0.2–2)	66 (15–146)	166 (8–395)	0.06 (0.003–0.1)	5 (0.2–12)	346 (156–915)	0.5 (0.3–0.9)	43 (23–71)
Enterotoxigenic *E*. *coli*	6 (2–13)	0 (0–0)	0.004 (0.001–0.01)	1,075 (229–3,521)	0.6 (0.1–1)	42 (10–104)	555 (43–2,430)	0.04 (0.003–0.1)	4 (0.3–10)	1,257 (714–2,206)	0.4 (0.2–0.6)	30 (17–51)
*Giardia* spp.	54 (16–123)	0 (0–0)	0.03 (0.009–0.1)	159 (16–903)	0 (0–0)	0.1 (0.01–0.9)	354 (8–1,519)	0 (0–0)	0.3 (0.005–1)	410 (188–828)	0 (0–0)	0.4 (0.2–0.8)
Norovirus	1,652 (630–3,294)	0.05 (0.02–0.1)	4 (1–8)	841 (113–5,631)	1 (0.2–3)	71 (15–230)	1,305 (189–6,441)	0.05 (0.004–0.2)	4 (0.4–17)	1,814 (1,022–3,653)	0.5 (0.2–1)	36 (17–80)
*Salmonella enterica*, non-typhoidal	186 (118–275)	0.1 (0.08–0.2)	8 (5–14)	908 (88–4,758)	0.7 (0.2–2)	49 (11–147)	898 (170–6,428)	0.02 (0.01–0.03)	2 (1–7)	1,140 (459–3,065)	0.4 (0.2–0.7)	32 (19–58)
*Shigella* spp.	3 (0.9–8)	0.003 (0–0.01)	0.2 (0.03–0.8)	1,084 (177–3,927)	0.3 (0.07–1)	25 (5–83)	689 (19–2,549)	0.04 (0.002–0.1)	4 (0.1–10)	741 (297–1,728)	0.2 (0.1–0.4)	18 (8–37)
Shiga toxin-producing *E*. *coli*	18 (9–28)	0.003 (0.001–0.006)	0.3 (0.1–0.8)	19 (2–95)	0.002 (0–0.01)	0.2 (0.02–1)	3 (2–6)	0 (0–0.001)	0.05 (0.02–0.1)	17 (11–37)	0.002 (0–0.005)	0.2 (0.09–0.5)
*Vibrio cholerae*	0.03 (0.01–0.06)	0 (0–0)	0 (0–0)	17 (0.7–52)	0.4 (0.007–2)	30 (0.5–109)	0.2 (0.01–0.5)	0.002 (0–0.005)	0.1 (0.005–0.3)	11 (5–23)	0.4 (0.1–0.7)	25 (10–51)
**Invasive enteric diseases**	**19 (9–51)**	**0.2 (0.1–0.3)**	**10 (7–19)**	**872 (169–2,288)**	**4 (1–9)**	**250 (81–598)**	**163 (30–391)**	**0.9 (0.3–2)**	**58 (19–134)**	**372 (146–847)**	**2 (1–4)**	**132 (71–254)**
*Brucella* spp.	4 (1–33)	0.02 (0.006–0.2)	1 (0.4–11)	2 (0.02–302)	0.01 (0–1)	0.8 (0.006–96)	2 (0.5–61)	0.01 (0.002–0.3)	0.6 (0.1–19)	6 (2–132)	0.03 (0.01–0.7)	2 (0.6–42)
Hepatitis A	11 (3–28)	0.02 (0.006–0.06)	1 (0.3–3)	494 (57–1,590)	1 (0.1–3)	48 (6–151)	51 (3–164)	0.1 (0.006–0.3)	5 (0.3–15)	199 (53–560)	0.4 (0.1–1)	20 (6–53)
*Listeria monocytogenes*	0.2 (0.2–0.3)	0.04 (0.03–0.06)	2 (1–2)	0.1 (0–2)	0.03 (0–0.6)	1 (0–21)	0.2 (0.1–0.4)	0.04 (0.03–0.09)	1 (1–3)	0.2 (0.09–1)	0.05 (0.02–0.3)	2 (0.7–11)
*Mycobacterium bovis*	0.2 (0.1–0.3)	0.02 (0.01–0.03)	0.9 (0.7–1)	2 (0.9–4)	0.2 (0.1–0.5)	13 (6–26)	0.8 (0.4–1)	0.04 (0.02–0.07)	2 (1–4)	2 (1–2)	0.2 (0.1–0.2)	9 (7–12)
*Salmonella* enterica., invasive non-typhoidal	0.8 (0.6–1)	0.07 (0.04–0.1)	3 (2–5)	2 (0.3–2)	0.2 (0.03–0.3)	9 (2–16)	1 (0.5–2)	0.1 (0.05–0.2)	6 (2–10)	4 (2–6)	0.4 (0.2–0.7)	26 (13–45)
*Salmonella enterica* Paratyphi A	0.2 (0.03–1)	0.002 (0–0.008)	0.1 (0.01–0.6)	58 (11–167)	0.4 (0.1–1)	29 (7–83)	18 (3–47)	0.1 (0.02–0.3)	8 (1–20)	25 (8–63)	0.2 (0.05–0.4)	12 (4–31)
*Salmonella enterica* Typhi	1 (0.1–5)	0.007 (0–0.03)	0.5 (0.05–2)	250 (50–725)	2 (0.4–5)	128 (29–361)	77 (12–203)	0.5 (0.07–1)	33 (5–87)	110 (34–272)	0.8 (0.2–2)	54 (17–133)
**TOTAL**	**2,506 (1,455–4,168)**	**0.5 (0.3–0.6)**	**32 (24–45)**	**8,068 (3,294–20,663)**	**9 (4–17)**	**622 (306–1,145)**	**6,491 (2,630–17,528)**	**1 (0.6–2)**	**93 (45–175)**	**8,369 (5,723–13,318)**	**5 (3–8)**	**366 (255–538)**

* Table does not include four foodborne intoxications due to *Clostridium botulinum*, *C*. *perfringens*, *S*. *aureus*, and *Bacillus cereus* due to a lack of data for global estimation.

** Includes Guillain-Barré Syndrome cases and deaths.

**Table 4:** Median number of foodborne illnesses, deaths, and Disability Adjusted Life Years (DALYs) by age group, with 95% uncertainty intervals, 2010.

PATHOGEN*	Age Group: <5 Years of Age			Age Group: ≥5 Years of Age			Ratio <5:≥5		
	ILLNESSES	DEATHS	DALYs	ILLNESSES	DEATHS	DALYs	ILLNESSES	DEATHS	DALYs
	Number (95% UI)	Number (95% UI)	Number (95% UI)	Number (95% UI)	Number (95% UI)	Number (95% UI)	Ratio <5:≥5 (95% UI)	Ratio <5:≥5 (95% UI)	Ratio <5:≥5 (95% UI)
**Diarrheal Disease**	**216,839,210 (148,937,428–309,926,253)**	**91,621 (62,442–132,707)**	**8,547,149 (5,903,945–12,254,175)**	**327,209,075 (179,670,939–643,705,133)**	**107,500 (69,907–163,979)**	**7,205,002 (4,790,026–10,747,526)**	**0.66 (0.32–1.28)**	**0.86 (0.60–1.16)**	**1.19 (0.86–1.60)**
*Campylobacter* spp.**	47,988,357 (22,436,891–102,663,926)	13,861 (8,754–23,670)	1,383,499 (911,878–2,279,897)	42,883,268 (18,350,672–112,061,441)	7,436 (4,930–9,974)	750,578 (540,003–956,663)	1.11 (0.34–3.47)	1.91 (1.21–3.08)	1.87 (1.26–2.92)
*Cryptosporidium* spp.	5,986,213 (2,569,532–12,738,924)	1,989 (678–5,683)	185,057 (64,847–518,497)	2,253,036 (774,628–8,639,265)	1,673 (638–4,149)	104,794 (40,408–256,055)	2.61 (0.69–8.01)	1.23 (0.42–2.72)	1.83 (0.65–3.93)
*Entamoeba histolytica*	8,480,759 (1,593,697–30,849,576)	896 (90–4,852)	92,213 (15,997–444,002)	17,828,477 (5,378,578–50,963,825)	524 (218–1,110)	43,984 (20,149–85,551)	0.48 (0.08–2.38)	1.75 (0.18–8.71)	2.14 (0.38–9.49)
Enteropathogenic *E*. *coli*	17,312,780 (6,767,766–54,104,398)	22,156 (11,944–37,473)	2,004,543 (1,084,856–3,389,584)	5,458,601 (2,145,370–16,561,005)	14,647 (7,305–25,447)	911,012 (457,215–1,575,768)	3.20 (0.85–11.78)	1.52 (1.03–2.29)	2.21 (1.52–3.29)
Enterotoxigenic *E*. *coli*	38,352,806 (21,144,875–64,795,160)	14,056 (7,045–26,784)	1,303,490 (668,837–2,446,758)	46,811,878 (20,306,649–103,801,449)	11,933 (6,382–18,887)	767,975 (419,834–1,204,273)	0.82 (0.35–1.96)	1.21 (0.63–2.10)	1.74 (0.95–2.93)
*Giardia* spp.	18,773,028 (8,075,497–38,649,748)	0 (0–0)	20,677 (8,552–44,101)	8,693,968 (3,337,657–24,195,602)	0 (0–0)	5,016 (1,945–13,791)	2.11 (0.84–5.22)	N/A	4.04 (1.57–10.28)
Norovirus	34,582,700 (19,595,826–59,592,939)	8,992 (4,251–19,347)	844,376 (406,822–1,776,252)	89,056,582 (46,054,795–206,532,318)	25,807 (11,201–61,642)	1,638,925 (730,924–3,844,771)	0.38 (0.19–0.73)	0.35 (0.22–0.54)	0.52 (0.33–0.78)
*Salmonella enterica*, non-typhoidal	15,274,234 (6,514,539–41,696,874)	12,531 (6,562–30,779)	1,149,675 (609,216–2,792,992)	60,293,254 (18,488,275–189,066,838)	15,807 (8,762–21,942)	1,016,047 (576,408–1,405,079)	0.26 (0.06–1.16)	0.84 (0.44–1.83)	1.19 (0.64–2.57)
*Shigella* spp.	15,516,627 (5,416,319–38,620,351)	8,863 (3,250–20,925)	819,280 (309,576–1,909,450)	34,049,173 (10,186,959–95,312,884)	6,060 (2,734–11,511)	404,144 (188,009–749,866)	0.45 (0.13–1.70)	1.49 (0.60–3.26)	2.06 (0.87–4.43)
Shiga toxin-producing *E*. *coli*	339,905 (217,805–728,708)	63 (30–170)	6,969 (3,278–17,751)	836,948 (536,302–1,794,298)	65 (24–204)	5,989 (2,654–15,877)	0.41 (0.41–0.41)	0.96 (0.81–1.31)	1.16 (1.09–1.29)
*Vibrio cholerae*	114,518 (46,636–235,152)	3,697 (1,546–7,506)	331,395 (138,538–672,643)	648,933 (264,273–1,332,530)	20,952 (8,758–42,535)	1,390,973 (581,491–2,820,499)	0.18 (0.18–0.18)	0.18 (0.18–0.18)	0.24 (0.24–0.24)
**Invasive enteric diseases**	**4,336,215 (1,675,945–9,422,681)**	**23,727 (11,866–45,950)**	**2,180,916 (1,085,765–4,219,254)**	**21,182,632 (8,375,340–49,059,198)**	**123,026 (69,306–230,318)**	**6,900,776 (3,799,471–13,355,093)**	**0.21 (0.15–0.23)**	**0.19 (0.14–0.22)**	**0.32 (0.23–0.35)**
*Brucella* spp.	4,144 (1,527–93,225)	21 (7–463)	1,988 (687–44,999)	389,106 (142,279–9,006,169)	1,936 (654–45,081)	122,904 (42,484–2,865,643)	0.01 (0.01–0.01)	0.01 (0.01–0.01)	0.02 (0.02–0.02)
Hepatitis A	2,165,243 (573,433–6,084,381)	4,380 (1,132–12,211)	411,592 (112,767–1,130,290)	11,544,593 (3,057,415–32,440,565)	23,351 (6,036–65,109)	941,278 (269,448–2,538,627)	0.19 (0.19–0.19)	0.19 (0.19–0.19)	0.44 (0.39–0.46)
*Listeria monocytogenes*	1,240 (393–10,502)	330 (126–2,138)	30,750 (11,700–198,862)	12,936 (5,716–80,766)	2,851 (1,200–18,271)	87,569 (36,830–561,221)	0.10 (0.07–0.13)	0.11 (0.08–0.16)	0.34 (0.24–0.49)
*Mycobacterium bovis*	869 (732–1,049)	76 (58–101)	7,134 (5,477–9,496)	120,398 (99,119–149,188)	10,470 (7,836–14,372)	600,639 (452,917–816,737)	0.01 (0.01–0.01)	0.01 (0.01–0.01)	0.01 (0.01–0.01)
*Salmonella enterica*, invasive non-typhoidal	45,549 (25,019–62,638)	4,700 (2,268–8,188)	421,523 (203,340–733,940)	239,467 (142,115–321,539)	24,692 (12,655–42,246)	1,373,635 (684,718–2,373,326)	0.19 (0.17–0.20)	0.19 (0.17–0.20)	0.31 (0.29–0.32)
*Salmonella enterica* Paratyphi A	357,814 (110,286–885,942)	2,480 (778–6,067)	227,507 (71,530–557,578)	1,383,306 (426,364–3,425,041)	9,588 (3,007–23,454)	627,953 (197,302–1,541,909)	0.26 (0.26–0.26)	0.26 (0.26–0.26)	0.36 (0.36–0.36)
*Salmonella enterica* Typhi	1,555,715 (479,504–3,851,923)	10,783 (3,381–26,377)	989,159 (311,001–2,424,250)	6,014,372 (1,853,758–14,891,483)	41,689 (13,072–101,973)	2,730,232 (857,835–6,703,954)	0.26 (0.26–0.26)	0.26 (0.26–0.26)	0.36 (0.36–0.36)
**TOTAL**	**221,451,463 (153,244,508–315,075,166)**	**116,613 (80,862–165,379)**	**10,831,919 (7,587,557–15,271,603)**	**350,711,509 (199,599,319–673,777,073)**	**232,916 (152,283–368,498)**	**14,250,088 (9,419,295–22,483,691)**	**0.63 (0.32–1.18)**	**0.50 (0.36–0.68)**	**0.76 (0.56–1.01)**

* Table does not include four foodborne intoxications due to *Clostridium botulinum*, *C*. *perfringens*, *S*. *aureus*, and *Bacillus cereus* due to a lack of data for global estimation.

** Includes Guillain-Barré Syndrome cases and deaths.

### Deaths

We estimated that the 22 diseases in our study caused 1.09 million (95% UI 0.89–1.37 million) deaths in 2010, 34% (95% UI 29–38%) in children <5 years of age. Among the diarrheal diseases, norovirus was responsible for the most deaths. Other diarrheal pathogens responsible for large numbers of deaths were EPEC, *V*. *cholerae*, and *Shigella* spp. The 37,600 (95% UI 27,700–55,100) deaths attributed to *Campylobacter* spp. included 1,310 (95% UI 887–1,880) deaths from GBS. Among the extra-intestinal enteric diseases, the pathogens resulting in the most deaths were *Salmonella* Typhi, hepatitis A virus, iNTS and *Salmonella* Paratyphi A.

Overall, the 22 diseases in our study resulted in 351,000 (95% UI 240,000–524,000) deaths due to contaminated food in 2010; 33% (95% UI 27–40%) in children <5 years of age. The enteric pathogens resulting in the most foodborne deaths were *Salmonella* Typhi, EPEC, norovirus, iNTS, NTS, and hepatitis A. The mortality rates of foodborne diseases were consistently highest in the African region followed by the South Eastern Asian region (Table 3). Among the 11 diarrheal diseases due to contaminated food, the rate ratio of deaths in children <5 years of age compared to those ≥5 years of age was 8.37 (95%UI 5.90–11.4). For all 22 diseases, the rate ratio of deaths in children <5 years of age compared to those ≥5 years of age was 4.85 (95%UI 3.54–6.59).

### DALYs

We estimated that the 22 diseases in our study caused 78.7 million (95% UI 65.0–97.7 million) DALYS in 2010, 43% (95% UI 38–48%) in children <5 years of age. The pathogens resulting in the most DALYs were norovirus, *Salmonella* Typhi, EPEC, *V*. *cholerae*, ETEC, and hepatitis A (Table 2).

**Fig 1:**
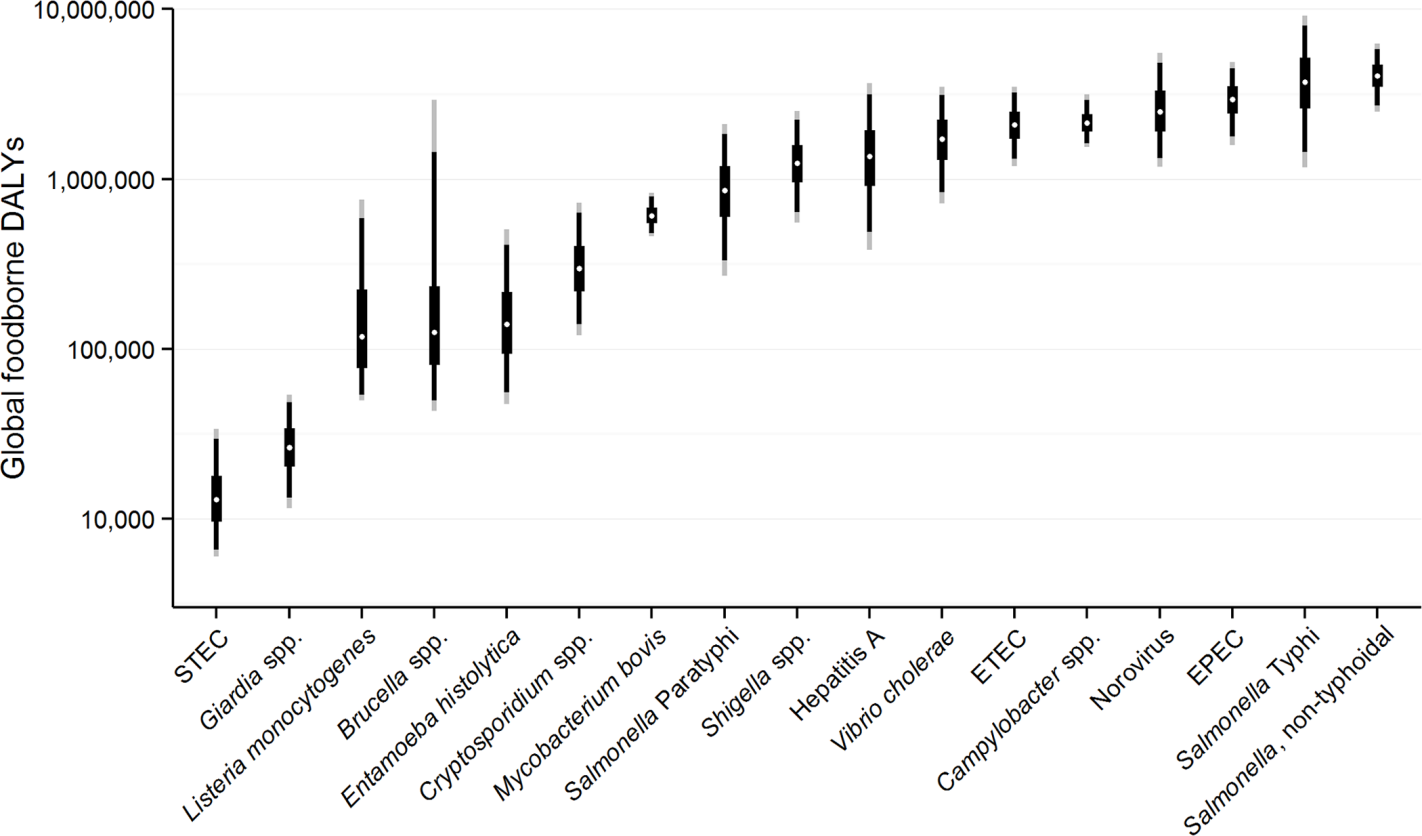
Disability Adjusted Life Years for each pathogen acquired from contaminated food ranked from lowest to highest with 95% Uncertainty Intervals, 2010. Note figure is on a logarithmic scale. The figure shows the median (white dot); Inter-Quartile Range = 50%UI = 25%/75% percentiles (thick black line); 90% UI = 5%/95% percentiles (thin black line); 95% UI = 2.5%/97.5% percentiles (thin grey line). Note, figure does not include four foodborne intoxications due to *Clostridium botulinum*, *C*. *perfringens*, *S*. *aureus*, and *Bacillus cereus* due to a lack of data for global estimation. In addition, data for non-typhoidal *Salmonella enterica* infections and invasive non-typhoidal *S*. *enterica* have been combined.

We estimate that the 22 diseases in our study resulted in 25.2 million (95% UI 17.5–37.0 million) DALYs due to contaminated food; 43% (95% UI 36–50%) in children <5 years of age. Fig 1 shows the relative burden of foodborne enteric infections, if iNTS and NTS were grouped together. The pathogen resulting in the most foodborne DALYs was nontyphoidal *S*. *enterica*, if iNTS were included (4.07 million DALYs; 95% UI 2.49–6.27 million DALYs). Other pathogens resulting in substantial foodborne DALYs included: *Salmonella* Typhi, EPEC, norovirus, and *Campylobacter* spp. The rates of DALYs for foodborne diseases were highest in the African region. Overall, the 22 diseases transmitted by contaminated food resulted in 10.8 million (95% UI 7.59–15.3 million) DALYs in children <5 years of age compared to 14.3 million (95% UI 9.42–22.5 million) DALYs in those ≥5 years of age.

## Discussion

To our knowledge, we estimate for the first time the substantial worldwide burden of foodborne bacterial, viral, and protozoal diseases in humans, particularly among children. Although children <5 years of age represent only 9% of the global population, we found that 43% of the disease burden from contaminated food occurred in this group. Foodborne illnesses from diarrheal and invasive non-typhoidal *S*. *enterica* resulted in the largest disease burden reflecting the ubiquitous nature of *Salmonella*, the severe nature of illness, and the fact that young children are commonly infected [19]. Large human disease burdens are also imposed by foodborne infections due to norovirus and typhoid. It is important to recognize that diseases with lower burden may still warrant intervention. In particular, certain foodborne diseases may represent a larger problem in some regions. For example, the most substantial burden due to foodborne cholera occurs in African and Asian regions. Similarly, the burden of brucellosis and *M*. *bovis* infections were highest in the Middle Eastern and African regions.

To develop these comprehensive estimates of the disease burden of foodborne diseases, we adopted an innovative approach to incorporate the highest quality data available for each foodborne disease [11]. Due to their quality, we gave highest priority to studies with national estimates of foodborne diseases. Since studies with national estimates were only available in a few countries, we adapted the CHERG approach for estimating the disease burden of diarrheal diseases [10,36]. This approach was facilitated by the availability of estimates of the envelope of diarrheal deaths, along with recent advances in diarrheal disease diagnosis, such as widespread application of polymerase chain reaction (PCR) for norovirus detection [20,49].

In our study, norovirus resulted in the largest number of cases of foodborne diseases and overall burden, highlighting the global importance of this agent [20]. However, the disease model we used in the 135 middle- and high-mortality counties included only norovirus infections that resulted in a diarrheal illness. If we also included estimates for norovirus infections that resulted in vomiting without diarrhea, there would be an estimated additional 163 million norovirus cases in these countries [50]. We also found, similar to what has been reported in national studies, that in the countries where we applied the modified CHERG approach, the etiological cause of almost half of diarrheal cases and deaths remained unknown. This was likely due, in large part, to pathogens that are possibly foodborne but with insufficient data for estimation, and unknown agents not yet discovered. In this study, we focused our attention on the burden due to pathogens that were known to be transmitted by contaminated food.

When we examined the human health impact of different pathogens, various serotypes of *Salmonella* resulted in the greatest foodborne burden. If we consider the combined burden attributable to *S*. *enterica* from all invasive (including iNTS, *Salmonella* Typhi and *Salmonella* Paratyphi A) and diarrheal infections, there were an estimated 8.76 million (95% UI 5.01–15.6 million) DALYs from all transmission sources and 6.43 million (95% UI 3.08–13.2 million) DALYs from contaminated food. This highlights the significant public health importance of *Salmonella* infections and the urgency of control, particularly for invasive infections in low- and middle-income settings where most of the mortality occurs [51–53].

Twelve of the diseases included in our study were also included in the GBD 2010 study [25,26,54]. For three diseases (typhoid, paratyphoid and hepatitis A) we used GBD 2010 data to derive estimates of incidence. For the other nine diarrheal diseases, we elected to conduct our own analysis or used updated data from commissioned systematic reviews to derive estimates. Our study builds upon the GBD 2010 study by providing estimates of the proportion of each disease acquired from food; we also provide, in addition to estimates of deaths, estimates of the number of illnesses for each of the diseases [26]. Before we applied our estimate of foodborne proportions to each pathogen, our estimates of the disease burden for a few pathogens, in terms of the estimated number of deaths and DALYs were relatively similar for diseases in common with GBD 2010 and FERG.

However, there were important differences in other estimates. The GBD 2010 study estimates of deaths due to *E*. *histolytica*, *Cryptosporidium* spp., and *Campylobacter* spp. were 10 times, 4 times and 3 times greater, respectively, than the FERG estimates [25]. The FERG estimate for DALYs for nontyphoidal *S*. *enterica* combining diarrheal and invasive infections was 4 times greater than GBD 2010 [54]. The FERG estimates are relatively similar to previous global estimates of cholera, typhoid fever, salmonellosis, and shigellosis [19,24,40,55]. The GBD 2010 study estimates included ‘cysts and liver abscesses’ as a complication of typhoid fever, which has been questioned [56]. However, we understood this categorization to be a proxy for serious typhoid fever and incorporated these data into our estimates. The CFR for each of the diseases included in our estimate are comparable to those reported in national studies. There is a continuing need for high quality studies assessing the foodborne disease burden at the national level. Our methodology for estimating the disease burden attributable to foodborne transmission can be used in future studies.

In comparing the overall burden of our findings, the diseases we included in our study resulted in 79 million DALYs in 2010. This represent approximately 3% of the 2.49 billion DALYs reported in the GBD 2010 study [54]. The GBD 2010 study estimated that approximately 25% of DALYs globally were due to deaths and disability in children younger than 5 years of age, while we estimated that 43% of the DALYs in our study were among children <5 years of age.

There are obvious policy implications of our findings. Countries and international agencies must prioritize food safety to prevent foodborne illness, particularly among young children. The highest burden of disease due to contaminated food was in the African region, largely due to iNTS in children. The considerable regional difference in the burden of foodborne diseases suggests that current control methods exist to avoid an important proportion of the current burden. Our results should stimulate research into the epidemiology of foodborne diseases, with a view to informing prevention efforts. There is an urgent need to identify and implement effective food hygiene interventions in low- and middle-income countries.

A limitation of our estimates of the consequences for human health of foodborne diseases is that, due to data gaps particularly in middle- and high-mortality countries, we included only a few sequelae in our estimates. We did not include data on post-infectious sequelae, such as reactive arthritis and irritable bowel syndrome following foodborne infections. Studies of the burden of enteric pathogens in low-mortality countries highlight that excluding these sequelae under-represents the true burden of disease, but reliable data were not available from middle- and high-mortality countries [57]. Where we did include sequelae, there were insufficient data to account for age-specific effects. We also excluded stillbirths; this exclusion only affected disease burden estimates for *L*. *monocytogenes*. A recent review estimated that listeriosis, which we assume is all foodborne, causes 273 stillbirths globally annually [18].

We were unable to distinguish between the effects on health from the condition under study and that of co-morbid conditions, which is common to many studies of human health. For example, salmonellosis and *M*. *bovis* infections may occur as HIV co-infections. If FERG included deaths among HIV-infected persons, there would have substantial additional deaths due to invasive nontyphoidal *S*. *enterica* deaths, some of which could presumably be averted by improvements in food safety. For some other pathogens in our estimates, such as *L*. *monocytogenes* and *Cryptosporidium* spp., we were unable to account for the excess mortality due to HIV infection due to a lack of reliable data.

Another important limitation of our attempt to quantify the disease burden due of foodborne disease is the inherent difficulty in estimating the proportion of illness acquired from food [58]. We relied on a structured expert elicitation study for these estimates. We were unable to estimate differences in mode of transmission by age, despite this potentially being important. Expert elicitation studies can result in highly variable proportions attributed to food, depending on the nature of experts included in elicitation studies [59,60]. Without specific studies attributing sources of infection, it is difficult to obtain accurate estimates of foodborne transmission, but this finding regarding the need for more attribution studies is an important outcome of our study [61]. For example, the FERG expert elicitation study estimated that 18% of norovirus was foodborne compared with 14% estimated from a recent study based on outbreak genotyping [21].

The major limitation of our study was the lack of reliable data from many regions of the world. In particular, for the most populous regions of the world we had the least data for some pathogens [10]. We tried to use the best data available and attempted to make reasoned assumptions wherever possible [8]. For some agents, such as toxin-mediated illnesses, we elected to limit our estimates to countries where diseases were endemic or where there was sufficient data. Further data on burden of enteric diseases from low- and middle-income settings, particularly high quality epidemiological data, are needed to improve our understanding of foodborne diseases [8,11].

Despite these limitations, our estimates of the disease burden due to 22 foodborne diseases should provide policy makers with information for advocacy for improved regulation and control of foodborne diseases. Of particular importance, we estimate that almost half of the burden of foodborne disease occurs in children under 5 years of age. This previously underappreciated disease burden requires greater attention from governments and resourcing to improve food safety. In our study we highlight regions where diseases, such as tuberculosis and brucellosis are still transmitted by contaminated food. Our results highlight the benefits of countries conducting national studies estimating and attributing the incidence, hospitalizations and deaths due to foodborne diseases to improve understanding of burden and improve control measures. Our regional estimates could be used to fill data gaps for countries attempting to estimate the burden of disease due to contaminated food.

## Supporting Information

S1 Text Glossary and regions. (DOCX)

S2 Text Information sheets for foodborne agents. (DOCX)

## References

[1] Stein C, Kuchenmuller T, Hendrickx S, Prüss-Űstün A, Wolfson L, Engels D, et al The Global Burden of Disease assessments—WHO is responsible? PLoS Negl Trop Dis. 2007;1:e161 10.1371/journal.pntd.0000161 18160984 PMC2154395

[2] Tauxe RV, Doyle MP, Kuchenmuller T, Schlundt J, Stein CE. Evolving public health approaches to the global challenge of foodborne infections. Int J Food Microbiol. 2010;139 Suppl 1:S16–28. 10.1016/j.ijfoodmicro.2009.10.014 19931203

[3] Scallan E, Hoekstra RM, Angulo FJ, Tauxe RV, Widdowson MA, Roy SL, et al Foodborne illness acquired in the United States—major pathogens. Emerg Infect Dis. 2011;17:7–15. 10.3201/eid1701.091101p1 21192848 PMC3375761

[4] Coulombier D, Takkinen J. From national to international—challenges in cross-border multi-country, multi-vehicle foodborne outbreak investigations. Eurosurveillance. 2013;18:20423 23517867 10.2807/ese.18.11.20423-en

[5] Bernard H, Faber M, Wilking H, Haller S, Hohle M, Schielke A, et al Large multistate outbreak of norovirus gastroenteritis associated with frozen strawberries, Germany, 2012. Eurosurveillance. 2014;19:20719 24602278 10.2807/1560-7917.es2014.19.8.20719

[6] Frank C, Werber D, Cramer JP, Askar M, Faber M, an der Heiden M, et al Epidemic profile of Shiga-toxin-producing Escherichia coli O104:H4 outbreak in Germany. New Engl J Med. 2011;365:1771–1780. 10.1056/NEJMoa1106483 21696328

[7] Hathaway SC. Food control from farm to fork: implementing the standards of Codex and the OIE. Rev Sci Tech Oie. 2013;32:479–485. 10.20506/rst.32.2.2247 24547651

[8] Haagsma JA, Polinder S, Stein CE, Havelaar AH. Systematic review of foodborne burden of disease studies: quality assessment of data and methodology. Int J Food Microbiol. 2013;166:34–47. 10.1016/j.ijfoodmicro.2013.05.029 23827806

[9] Kuchenmuller T, Hird S, Stein C, Kramarz P, Nanda A, Havelaar AH. Estimating the global burden of foodborne diseases—a collaborative effort. Eurosurveillance. 2009;14. 10.2807/ese.14.18.19195-en 19422776

[10] Lanata CF, Fischer-Walker CL, Olascoaga AC, Torres CX, Aryee MJ, Black RE, et al Global causes of diarrheal disease mortality in children <5 years of age: a systematic review. PLoS ONE. 2013;8:e72788 10.1371/journal.pone.0072788 24023773 PMC3762858

[11] Pires SM, Fischer-Walker CL, Lanata CF, Devleesschauwer B, Hall AJ, Kirk MD, et al Aetiology-specific estimates of the global and regional incidence and mortality of diarrhoeal diseases commonly transmitted through food. PLoS ONE. 2015; 10: e 0142927 10.1371/journal.pone.0142927 PMC4668836 26632843

[12] Fischer Walker CL, Sack D, Black RE. Etiology of diarrhea in older children, adolescents and adults: a systematic review. PLoS Negl Trop Dis. 2010;4:e768 10.1371/journal.pntd.0000768 20689809 PMC2914743

[13] Walker CL, Black RE. Diarrhoea morbidity and mortality in older children, adolescents, and adults. Epidemiol Infect. 2010;138:1215–1226. 10.1017/S0950268810000592 20307341

[14] Dean AS, Crump L, Greter H, Hattendorf J, Schelling E, Zinsstag J. Clinical manifestations of human brucellosis: a systematic review and meta-analysis. PLoS Negl Trop Dis. 2012;6:e1929 10.1371/journal.pntd.0001929 23236528 PMC3516581

[15] Dean AS, Crump L, Greter H, Schelling E, Zinsstag J. Global burden of human brucellosis: a systematic review of disease frequency. PLoS Negl Trop Dis. 2012;6:e1865 10.1371/journal.pntd.0001865 23145195 PMC3493380

[16] Durr S, Muller B, Alonso S, Hattendorf J, Laisse CJ, van Helden PD, et al Differences in primary sites of infection between zoonotic and human tuberculosis: results from a worldwide systematic review. PLoS Negl Trop Dis. 2013;7:e2399 10.1371/journal.pntd.0002399 24009789 PMC3757065

[17] Muller B, Durr S, Alonso S, Hattendorf J, Laisse CJ, Parsons SD, et al Zoonotic Mycobacterium bovis-induced tuberculosis in humans. Emerg Infect Dis. 2013;19:899–908. 10.3201/eid1906.120543 23735540 PMC4816377

[18] Maertens-de-Noordhout C, Devleesschauwer B, Angulo FJ, Verbeke G, Haagsma J, Kirk M, et al The global burden of listeriosis: a systematic review and meta-analysis. Lancet Infect Dis. 2014;14:1073–1082. 10.1016/S1473-3099(14)70870-9 25241232 PMC4369580

[19] Majowicz SE, Scallan E, Jones-Bitton A, Sargeant JM, Stapleton J, Angulo FJ, et al Global Incidence of Human Shiga Toxin-Producing Escherichia coli Infections and Deaths: A Systematic Review and Knowledge Synthesis. Foodborne Pathog Dis. 2014;11:447–455. 10.1089/fpd.2013.1704 24750096 PMC4607253

[20] Ahmed SM, Hall AJ, Robinson AE, Verhoef L, Premkumar P, Parashar UD, et al Global Prevalence of Norovirus Among Cases of Gastroenteritis. Lancet Infect Dis. 2014;14:725–730. 10.1016/S1473-3099(14)70767-4 24981041 PMC8006533

[21] Verhoef L, Hewitt J, Barclay L, Ahmed SM, Lake R, Hall AJ, et al Norovirus Genotype Profiles Associated with Foodborne transmission. Emerg Infect Dis. 2015;21:592–599. 10.3201/eid2104.141073 25811368 PMC4378480

[22] Ao T, Feasy N, Gordon M, Keddy K, Angulo F, Crump J. Global burden of invasive nontyphoidal Salmonella disease, 2010. Emerg Infect Dis. 2015;21:941–949. 10.3201/eid2106.140999 PMC4451910 25860298

[23] Devleesschauwer B, Haagsma JA, Angulo FJ, Bellinger DC, Cole D, Döpfer D, et al (2015) Methodological Framework for World Health Organization Estimates of the Global Burden of Foodborne Disease. PLoS ONE 10: e 0142498 10.1371/journal.pone.0142498 PMC4668830 26633883

[24] Ali M, Lopez AL, You YA, Kim YE, Sah B, Maskery B, et al The global burden of cholera. Bulletin World Health Organization. 2012;90:209–218A. 10.2471/BLT.11.093427 PMC3314202 22461716

[25] Lozano R, Naghavi M, Foreman K, Lim S, Shibuya K, Aboyans V, et al Global and regional mortality from 235 causes of death for 20 age groups in 1990 and 2010: a systematic analysis for the Global Burden of Disease Study 2010. Lancet. 2012;380:2095–2128. 10.1016/S0140-6736(12)61728-0 23245604 PMC10790329

[26] Murray CJ, Ezzati M, Flaxman AD, Lim S, Lozano R, Michaud C, et al GBD 2010: design, definitions, and metrics. Lancet. 2012;380:2063–2066. 10.1016/S0140-6736(12)61899-6 23245602

[27] Ezzati M, Lopez AD, Rodgers A, Vander Hoorn S, Murray CJL. Selected major risk factors and global and regional burden of disease. Lancet. 2002;360(9343):1347–1360. 12423980 10.1016/S0140-6736(02)11403-6

[28] Scallan E, Griffin PM, Angulo FJ, Tauxe RV, Hoekstra RM. Foodborne illness acquired in the United States—unspecified agents. Emerg Infect Dis. 2011;17:16–22. 10.3201/eid1701.091101p2 21192849 PMC3204615

[29] Thomas MK, Murray R, Flockhart L, Pintar K, Pollari F, Fazil A, et al Estimates of the burden of foodborne illness in Canada for 30 specified pathogens and unspecified agents, circa 2006. Foodborne Pathog Dis. 2013;10:639–648. 10.1089/fpd.2012.1389 23659355 PMC3696931

[30] Havelaar AH, Haagsma JA, Mangen MJ, Kemmeren JM, Verhoef LP, Vijgen SM, et al Disease burden of foodborne pathogens in the Netherlands, 2009. Int J Food Microbiol. 2012;156:231–238. 10.1016/j.ijfoodmicro.2012.03.029 22541392

[31] Ford L, Glass K, Kirk M, Hall K. The burden of sequelae due to five pathogens acquired from contaminated food in Australia Circa 2010. Emerg Infect Dis. 2014;20:1865–1871. 10.3201/eid2011.131316 25340885 PMC4214289

[32] Kirk M, Ford L, Glass K, Hall K. Foodborne illness, Australia, circa 2000 and circa 2010. Emerg Infect Dis. 2014;20:1857–1864. 10.3201/eid2011.131315 25340705 PMC4214288

[33] Vaillant V, de Valk H, Baron E, Ancelle T, Colin P, Delmas MC, et al Foodborne infections in France. Foodborne Pathog Dis. 2005;2:221–232. 16156703 10.1089/fpd.2005.2.221

[34] Tam CC, Rodrigues LC, Viviani L, Dodds JP, Evans MR, Hunter PR, et al Longitudinal study of infectious intestinal disease in the UK (IID2 study): incidence in the community and presenting to general practice. Gut. 2012;61:69–77. 10.1136/gut.2011.238386 21708822 PMC3230829

[35] Lake RJ, Cressey PJ, Campbell DM, Oakley E. Risk ranking for foodborne microbial hazards in New Zealand: burden of disease estimates. Risk Analysis. 2010;30:743–752. 10.1111/j.1539-6924.2009.01269.x 19645753

[36] Walker CL, Rudan I, Liu L, Nair H, Theodoratou E, Bhutta ZA, et al Global burden of childhood pneumonia and diarrhoea. Lancet. 2013;381:1405–1416. 10.1016/S0140-6736(13)60222-6 23582727 PMC7159282

[37] Global Health Observatory (GHO) data [Internet]. World Health Organization. [cited 6 June 2014]. Available: http://www.who.int/gho/en/

[38] McGrogan A, Madle GC, Seaman HE, de Vries CS. The epidemiology of Guillain-Barre syndrome worldwide. A systematic literature review. Neuroepidemiology. 2009;32:150–163. 10.1159/000184748 19088488

[39] Poropatich KO, Walker CL, Black RE. Quantifying the association between Campylobacter infection and Guillain-Barre syndrome: a systematic review. J Health Popul Nutr. 2010;28:545–552. 21261199 10.3329/jhpn.v28i6.6602 PMC2995022

[40] Crump JA, Luby SP, Mintz ED. The global burden of typhoid fever. Bulletin World Health Organization. 2004;82:346–353. PMC2622843 15298225

[41] Biggs HM, Lester R, Nadjm B, Mtove G, Todd JE, Kinabo GD, et al Invasive Salmonella infections in areas of high and low malaria transmission intensity in Tanzania. Clin Infect Dis. 2014;58:638–647. 10.1093/cid/cit798 24336909 PMC3922215

[42] Mtove G, Amos B, von Seidlein L, Hendriksen I, Mwambuli A, Kimera J, et al Invasive salmonellosis among children admitted to a rural Tanzanian hospital and a comparison with previous studies. PLoS ONE. 2010;5:e9244 10.1371/journal.pone.0009244 20168998 PMC2821934

[43] Koch K, Kristensen B, Holt HM, Ethelberg S, Mølbak K, Schønheyder HC. International travel and the risk of hospitalization with non-typhoidal Salmonella bacteremia. A Danish population-based cohort study, 1999–2008. BMC Infect Dis. 2011;11:277 10.1186/1471-2334-11-277 22011371 PMC3206861

[44] World Animal Health Information System (WAHIS) [Internet]. 2012. Available: http://www.oie.int/wahis_2/public/index.php/home

[45] Anon. Global tuberculosis report 2013. WHO/HTM/TB/201311. Geneva: The World Health Organization; 2014.

[46] Hald T, Aspinall W, Devleesschauwer B, Cooke R, Corrigan T, Havelaar AH, et al (2015) World Health Organization estimates of the relative contributions of food to the burden of disease due to selected foodborne hazards: a structured expert elicitation. PLoS ONE. In Press. 10.1371/journal.pone.0145839 PMC4718673 26784029

[47] Salomon JA, Vos T, Hogan DR, Gagnon M, Naghavi M, Mokdad A, et al Common values in assessing health outcomes from disease and injury: disability weights measurement study for the Global Burden of Disease Study 2010. Lancet. 2012;380:2129–2143. 10.1016/S0140-6736(12)61680-8 23245605 PMC10782811

[48] Country-level population data for 2010 [Internet]. United Nations. [cited 14 August 2014]. Available: http://esa.un.org/wpp/

[49] Kotloff KL, Nataro JP, Blackwelder WC, Nasrin D, Farag TH, Panchalingam S, et al Burden and aetiology of diarrhoeal disease in infants and young children in developing countries (the Global Enteric Multicenter Study, GEMS): a prospective, case-control study. Lancet. 2013;382:209–222. 10.1016/S0140-6736(13)60844-2 23680352

[50] Hall AJ. Estimating the global burden of foodborne norovirus Foodborne Disease Epidemiology Reference Group meeting; Geneva, Switzerland: World Health Organization; 2013.

[51] Crump JA, Heyderman RS. Invasive Salmonella infections in Africa. T Roy Soc Trop Med H. 2014;108:673–675. 10.1093/trstmh/tru152 25301531

[52] Feasey NA, Dougan G, Kingsley RA, Heyderman RS, Gordon MA. Invasive non-typhoidal salmonella disease: an emerging and neglected tropical disease in Africa. Lancet. 2012;379:2489–2499. 10.1016/S0140-6736(11)61752-2 22587967 PMC3402672

[53] Majowicz SE, Musto J, Scallan E, Angulo FJ, Kirk M, O'Brien SJ, et al The global burden of nontyphoidal Salmonella gastroenteritis. Clin Infect Dis. 2010;50:882–889. 10.1086/650733 20158401

[54] Murray CJ, Vos T, Lozano R, Naghavi M, Flaxman AD, Michaud C, et al Disability-adjusted life years (DALYs) for 291 diseases and injuries in 21 regions, 1990–2010: a systematic analysis for the Global Burden of Disease Study 2010. Lancet. 2012;380:2197–2223. 10.1016/S0140-6736(12)61689-4 23245608

[55] Ram PK, Crump JA, Gupta SK, Miller MA, Mintz ED. Part II. Analysis of data gaps pertaining to Shigella infections in low and medium human development index countries, 1984–2005. Epidemiol Infect. 2008;136:577–603. 17686195 10.1017/S0950268807009351 PMC2870860

[56] Crump JA. Updating and refining estimates of typhoid fever burden for public health action. Lancet Global Health. 2014;2:e551–553. 10.1016/S2214-109X(14)70306-7 25304622 PMC4404498

[57] Plass D, Mangen MJ, Kraemer A, Pinheiro P, Gilsdorf A, Krause G, et al The disease burden of hepatitis B, influenza, measles and salmonellosis in Germany: first results of the Burden of Communicable Diseases in Europe Study. Epidemiol Infect. 2014:1–12. 10.1017/S0950268813003312 PMC9151280 24480146

[58] Pires SM. Assessing the applicability of currently available methods for attributing foodborne disease to sources, including food and food commodities. Foodborne Pathog Dis. 2013;10:206–213. 10.1089/fpd.2012.1134 23489045

[59] Vally HG, K., Ford L, Hall G, Kirk M, Shadbolt C, Veitch M, et al The proportion of illness acquired by foodborne transmission for nine enteric pathogens in Australia: an expert elicitation Foodborne Pathog Dis. 2014;11:727–733. 10.1089/fpd.2014.1746 25072416

[60] Ravel A, Davidson VJ, Ruzante JM, Fazil A. Foodborne proportion of gastrointestinal illness: estimates from a Canadian expert elicitation survey. Foodborne Pathog Dis. 2010;7:1463–1472. 10.1089/fpd.2010.0582 20704505

[61] Pires SM, Vieira AR, Hald T, Cole D. Source Attribution of Human Salmonellosis: An Overview of Methods and Estimates. Foodborne Pathog Dis. 2014. 10.1089/fpd.2014.1744 PMC10938214 24885917

