# Correction: World Health Organization Estimates of the Global and Regional Disease Burden of 22 Foodborne Bacterial, Protozoal, and Viral Diseases, 2010: A Data Synthesis

**DOI:** 10.1371/journal.pmed.1001940

**Published:** 2015-12-23

**Authors:** Martyn D. Kirk, Sara M. Pires, Robert E. Black, Marisa Caipo, John A. Crump, Brecht Devleesschauwer, Dörte Döpfer, Aamir Fazil, Christa L. Fischer-Walker, Tine Hald, Aron J. Hall, Karen H. Keddy, Robin J. Lake, Claudio F. Lanata, Paul R. Torgerson, Arie H. Havelaar, Frederick J. Angulo

There is an error in the fourth paragraph of the Discussion, second sentence. The correct sentence is: If we consider the combined burden attributable to *S. enterica* from all invasive (including iNTS, *Salmonella* Typhi and *Salmonella* Paratyphi A) and diarrheal infections, there were an estimated 21.2 million (95% UI 12.8–35.8 million) DALYs from all transmission sources and 8.76 million (95% UI 5.01–15.6 million) DALYs from contaminated food.
