# Supplementary material for: World Health Organization Estimates of the Global and Regional Disease Burden of 22 Foodborne Bacterial, Protozoal, and Viral Diseases, 2010: A Data Synthesis
Source: PLoS Med. 2015 Dec 3;12(12):e1001921. doi: 10.1371/journal.pmed.1001921 (PMC4668831; doi:10.1371/journal.pmed.1001921)
Supplement: S1 Text — (DOCX) [file pmed.1001921.s001.docx]

**S1 Text.**

**GLOSSARY**

*Foodborne disease*

A foodborne disease (FBD) can be defined as a disease commonly transmitted through ingested food. FBDs comprise a broad group of illnesses, and may be caused by microbial pathogens, parasites, chemical contaminants and biotoxins.

*Burden of disease*

In the context of this Initiative, the term “burden of disease” follows the principles of the Global Burden of Disease Study, and includes the quantification of morbidity, all disabling complications and mortality in a single summary measure (DALY).

*DALY (disability-adjusted life year)*

A health gap measure that combines the years of life lost due to premature death (YLL) and the years lived with disability (YLD) from a disease or condition, for varying degrees of severity, making time itself the common metric for death and disability. One DALY equates to one year of healthy life lost.

*Food*

According to the Codex Alimentarius Commission, “food means any substance, whether processed, semi-processed or raw, which is intended for human consumption, and includes drink, chewing gum and any substance which has been used in the manufacture, preparation or treatment of food but does not include cosmetics or tobacco or substances used only as drugs”(Murray *et al.*, 2013). The definition includes all bottled drinks.

*Source attribution*

Source attribution (SA) is the partitioning of the human burden of a particular disease to specific sources. With regards to foodborne diseases, SA can be conducted at various points along the food distribution chain, from the animal reservoir to the point of consumption.

**ABBREVIATIONS**

AFR WHO African Region

AMR WHO Region of the Americas

CI Confidence Interval

CSTF Country Studies Task Force of FERG

CHERG  Child Health Epidemiology Reference Group

CSTF Country Studies Task Force of FERG

CTF Computational Task Force of FERG

CTTF Chemicals and Toxins Task Force

DALY disability-adjusted life year

EAggEC Enteroaggerative E. coli

EDTF Enteric Disease Task Force of FERG

EE Expert Elicitation

EMR WHO Eastern Mediterranean Region

EPEC enteropathogenic Escherichia coli

ETEC enterotoxigenic Escherichia coli

EUR WHO European Region

FAO Food and Agriculture Organization of the United Nations

FERG Foodborne Disease Burden Epidemiology Reference Group

GBD Global Burden of Disease

GBS Guillain-Barré Syndrome

HAV Hepatitis A Virus

iNTS Invasive Non-typhoidal *Salmonella enterica* infection

NTS Non-typhoidal *Salmonella enterica* infection

OIE World Organisation for Animal Health

PAHO Pan American Health Organization

PDTF Parasitic Diseases Task Force of FERG

SATF Source Attribution Task Force of FERG

SEAR WHO South-East Asian Region

STEC Shiga-toxin-producing Escherichia coli

WHO World Health Organization

YLD years lived with disability

YLL years of life lost

WORLD HEALTH ORGANIZATION REGIONS

| Subregions^1^ | WHO member states |
| --- | --- |
| AFR D | Algeria; Angola; Benin; Burkina Faso; Cameroon; Cape Verde; Chad; Comoros; Equatorial Guinea; Gabon; Gambia; Ghana; Guinea; Guinea-Bissau; Liberia; Madagascar; Mali; Mauritania; Mauritius; Niger; Nigeria; Sao Tome and Principe; Senegal; Seychelles; Sierra Leone; Togo. |
| AFR E | Botswana; Burundi; Central African Republic; Congo; Côte d'Ivoire; Democratic Republic of the Congo; Eritrea; Ethiopia; Kenya; Lesotho; Malawi; Mozambique; Namibia; Rwanda; South Africa; Swaziland; Uganda; United Republic of Tanzania; Zambia; Zimbabwe. |
| AMR A | Canada; Cuba; United States of America. |
| AMR B | Antigua and Barbuda; Argentina; Bahamas; Barbados; Belize; Brazil; Chile; Colombia; Costa Rica; Dominica; Dominican Republic; El Salvador; Grenada; Guyana; Honduras; Jamaica; Mexico; Panama; Paraguay; Saint Kitts and Nevis; Saint Lucia; Saint Vincent and the Grenadines; Suriname; Trinidad and Tobago; Uruguay; Venezuela (Bolivarian Republic of). |
| AMR D | Bolivia (Plurinational State of); Ecuador; Guatemala; Haiti; Nicaragua; Peru. |
| EMR B | Bahrain; Iran (Islamic Republic of); Jordan; Kuwait; Lebanon; Libyan Arab Jamahiriya; Oman; Qatar; Saudi Arabia; Syrian Arab Republic; Tunisia; United Arab Emirates. |
| EMR D | Afghanistan; Djibouti; Egypt; Iraq; Morocco; Pakistan; Somalia; South Sudan^2^; Sudan; Yemen. |
| EUR A | Andorra; Austria; Belgium; Croatia; Cyprus; Czech Republic; Denmark; Finland; France; Germany; Greece; Iceland; Ireland; Israel; Italy; Luxembourg; Malta; Monaco; Netherlands; Norway; Portugal; San Marino; Slovenia; Spain; Sweden; Switzerland; United Kingdom. |
| EUR B | Albania; Armenia; Azerbaijan; Bosnia and Herzegovina; Bulgaria; Georgia; Kyrgyzstan; Montenegro; Poland; Romania; Serbia; Slovakia; Tajikistan; The Former Yugoslav Republic of Macedonia; Turkey; Turkmenistan; Uzbekistan. |
| EUR C | Belarus; Estonia; Hungary; Kazakhstan; Latvia; Lithuania; Republic of Moldova; Russian Federation; Ukraine. |
| SEAR B | Indonesia; Sri Lanka; Thailand. |
| SEAR D | Bangladesh; Bhutan; Democratic People's Republic of Korea; India; Maldives; Myanmar; Nepal; Timor-Leste. |
| WPR A | Australia; Brunei Darussalam; Japan; New Zealand; Singapore. |
| WPR B | Cambodia; China; Cook Islands; Fiji; Kiribati; Lao People's Democratic Republic; Malaysia; Marshall Islands; Micronesia (Federated States of); Mongolia; Nauru; Niue; Palau; Papua New Guinea; Philippines; Republic of Korea; Samoa; Solomon Islands; Tonga; Tuvalu; Vanuatu; Viet Nam. |

^1^ The subregions are defined on the basis of child and adult mortality as described by Ezzati et al ([1](file:///C:\Users\u3853379.UDS\AppData\Local\Microsoft\Windows\Temporary%20Internet%20Files\Content.Outlook\FFFG6DNE\Table%202-revised.docx#_ENREF_1)). Stratum A: very low child and adult mortality, Stratum B: low child mortality and very low adult mortality, Stratum C: low child mortality and high adult mortality, Stratum D: high child and adult mortality, and Stratum E: high child mortality and very high adult mortality. The use of the term ‘subregion’ here and throughout the text does not identify an official grouping of WHO Member States, and the “subregions” are not related to the six official WHO regions.
AFR = African Region; AMR = Region of the Americas; EMR = Eastern Mediterranean Region; EUR = European Region; SEAR = South-East Asia Region; WPR = Western Pacific Region.

^2^ South Sudan was reassigned to the WHO African Region in May 2013. As this study relates to time periods prior to this date, estimates for South Sudan were included in the WHO Eastern Mediterranean Region.

1. Ezzati M, Lopez AD, Rodgers A, Vander Hoorn S, Murray CJ, Comparative Risk Assessment Collaborating G. Selected major risk factors and global and regional burden of disease. Lancet. 2002;360(9343):1347-60.
