# Supplementary material for: World Health Organization Estimates of the Global and Regional Disease Burden of 22 Foodborne Bacterial, Protozoal, and Viral Diseases, 2010: A Data Synthesis
Source: PLoS Med. 2015 Dec 3;12(12):e1001921. doi: 10.1371/journal.pmed.1001921 (PMC4668831; doi:10.1371/journal.pmed.1001921)
Supplement: S2 Text — (DOCX) [file pmed.1001921.s002.docx]

# S2 Text. Information sheets for foodborne agents.

1. **Brucellosis**

| INCIDENCE: | We identified 32 countries as "free of brucellosis in livestock" using 2006-2012 data reported to the World Organisation for Animal Health (OIE) [[1](#_ENREF_1_1)] and a list of European countries recognized by the European Union as "officially brucellosis free" in cattle, sheep and goats in 2010 [[2](#_ENREF_1_2)]. Using 2001-2004 OIE data, a previous review [[3](#_ENREF_1_3)] estimated human brucellosis incidence for 9 of the countries we identified as free of brucellosis in livestock. The median human brucellosis incidence from these 9 countries free of brucellosis in livestock was used as the estimated human brucellosis incidence for each of the 32 countries free of brucellosis in livestock. We then used a FERG-commissioned systematic review which screened 2,385 articles [[4](#_ENREF_1_4)] and a literature review for national human brucellosis incidence estimates [[5-10](#_ENREF_1_5)], to extract brucellosis national incidence estimates for 17 countries (Argentina, Canada, Chad, China, Egypt, France, Greece, Iraq, Iran, Italy, Kyrgyzstan, Jordan, Mexico, Oman, Saudi Arabia, Turkey, and the United States); we compared the human brucellosis incidence estimates in each of these countries to human brucellosis incidence estimate in the same country in the previous review which used 2001-2004 OIE data [[3](#_ENREF_1_3)] to estimate a multiplier (mean=5.4, range 1.6-15.4) to account for under-reporting. We used this multiplier to estimate national human brucellosis incidence for countries with OIE human brucellosis data in the previous review but without national human brucellosis incidence estimates identified in the systematic review or literature review by multiplying the human brucellosis incidence reported to OIE by the multiplier; there were 32 such countries. These steps yielded human brucellosis incidence estimates in 81 countries. We then used the FERG Computational Task Force imputation model to impute an incidence of human brucellosis in all countries with missing incidence. |
| --- | --- |
| CLINICAL OUTCOMES: | FERG-commissioned systematic review assisted in determining the clinical outcomes for human brucellosis [[11](#_ENREF_1_11)]. These were: acute brucellosis (severe), acute brucellosis (moderate), chronic brucellosis, brucellosis orchitis, and brucellosis death. For acute brucellosis, we assumed 50% of cases were severe and 50% of cases were moderate [[11](#_ENREF_1_11)]. We assumed that 40% of brucellosis cases resulted in chronic brucellosis, and 10% of brucellosis cases in males resulted in orchitis [[11](#_ENREF_1_11)]. |
| DURATION: | Acute brucellosis: duration 14 days (minimum duration 7 days - maximum duration 21 days). Chronic brucellosis: duration 6 months (minimum duration 3 months - maximum duration 24 months). Brucellosis orchitis: duration 6 months (minimum duration 3 months - maximum duration 24 months) [[11](#_ENREF_1_11)]. |
| DISABILITY WEIGHT: | Acute brucellosis (severe): GBD 2010 disability weight of 0.210 (95% CI 0.139-0.298) for infectious disease, acute episode, severe. Acute brucellosis (moderate): GBD 2010 disability weight of 0.053 (95% CI 0.033-0.081) for infectious disease, acute episode, mild. Chronic brucellosis: GBD 2010 disability weight 0.079 (95%CI 0.053-0.115) for musculoskeletal problems, legs, moderate. Brucellosis orchitis: GBD 2010 disability weight of 0.097 (95% CI 0.063-0.0137) for epididymo-orchitis [[12](#_ENREF_1_12)]. |
| MORTALITY | Acute brucellosis and chronic brucellosis case fatality ratio 0.05% (minimum case fatality ratio 0.025% - maximum case fatality rate 0.075%) [[13](#_ENREF_1_13), [14](#_ENREF_1_14)]. |
| AGE DISTRIBUTION | Acute brucellosis, chronic brucellosis, brucellosis orchitis, and brucellosis death age distribution: 3% <15 years, 29% 15-24 years, 24% 25-34 years, 16% 35-44 years, 13% 45-54 years, 12% 55-64 years, 3% >65 years [[15](#_ENREF_1_15)]. |
| SEX DISTRIBUTION | Acute brucellosis, chronic brucellosis and brucellosis deaths sex distribution: 55% male (95% CI 50% - 60% male) [[11](#_ENREF_1_11)]. Brucellosis orchitis 100% male. |

References

1. World Organisation for Animal Health Information Database (WAHID) at the World Organisation for Animal Health (OIE) [Internet]. [cited November 1, 2013]. Available from: <http://www.oie.int/wahis_2/public/wahid.php/Diseaseinformation/diseasehome>.

2. The European Union summary report on trends and sources of zoonoses, zoonotic agents and food-borne outbreaks in 2010. Euro Surveill. 2012;17.

3. Pappas G, Papadimitriou P, Akritidis N, Christou L, Tsianos EV. The new global map of human brucellosis. Lancet Infect Dis. 2006;6:91-99.

4. Dean AS, Crump L, Greter H, Schelling E, Zinsstag J. Global burden of human brucellosis: a systematic review of disease frequency. PLoS Negl Trop Dis. 2012;6:e1865.

5. Zhong Z, Yu S, Wang X, Dong S, Xu J, Wang Y, et al. Human brucellosis in the People's Republic of China during 2005-2010. Int J Infect Dis. 2013;17:e289-292.

6. Cressey P, Lake R. Estimated incidence of foodborne illness in New Zealand: Application of overseas models and multipliers. Report—New Zealand Government, Christ Church, New Zealand. 2011.

7. Gkogka E, Reij MW, Havelaar AH, Zwietering MH, Gorris LG. Risk-based estimate of effect of foodborne diseases on public health, Greece. Emerg Infect Dis. 2011;17:1581-1590.

8. Vaillant V, de Valk H, Baron E, Ancelle T, Colin P, Delmas MC, et al. Foodborne infections in France. Foodborne Pathog Dis. 2005;2:221-232.

9. Thomas MK, Murray R, Flockhart L, Pintar K, Pollari F, Fazil A, et al. Estimates of the burden of foodborne illness in Canada for 30 specified pathogens and unspecified agents, circa 2006. Foodborne Pathog Dis. 2013;10:639-648.

10. Scallan E, Griffin PM, Angulo FJ, Tauxe RV, Hoekstra RM. Foodborne illness acquired in the United States--unspecified agents. Emerg Infect Dis. 2011;17:16-22.

11. Dean AS, Crump L, Greter H, Hattendorf J, Schelling E, Zinsstag J. Clinical manifestations of human brucellosis: a systematic review and meta-analysis. PLoS Negl Trop Dis. 2012;6:e1929.

12. Salomon JA, Vos T, Hogan DR, Gagnon M, Naghavi M, Mokdad A, et al. Common values in assessing health outcomes from disease and injury: disability weights measurement study for the Global Burden of Disease Study 2010. Lancet. 2012;380:2129-2143.

13. Al-Tawfiq JA. Therapeutic options for human brucellosis. Expert Rev Anti Infect Ther. 2008;6:109-120.

14. Al Dahouk S, Neubauer H, Hensel A, Schoneberg I, Nockler K, Alpers K, et al. Changing epidemiology of human brucellosis, Germany, 1962-2005. Emerg Infect Dis. 2007;13:1895-1900.

15. Buzgan T, Karahocagil MK, Irmak H, Baran AI, Karsen H, Evirgen O, et al. Clinical manifestations and complications in 1028 cases of brucellosis: a retrospective evaluation and review of the literature. Int J Infect Dis. 2010;14:e469-478.

1. ***Mycobacterium bovis* infections**

| INCIDENCE: | We identified 51 countries as "free of *Mycobacterium bovis* in cattle" using 2005-2012 data reported to the World Organisation for Animal Health [[1](#_ENREF_2_1)] and a list of European countries recognized by the European Union as "officially free of bovine tuberculosis" in 2010 [[2](#_ENREF_2_2)]. A FERG-commissioned systematic review screened 1,203 [[3](#_ENREF_2_3)]articles data from 91 countries estimated the median proportion of human tuberculosis cases due to *M. bovis* at the WHO region level as 2.8% for AFR, 0.4% for EUR, and 0.3% for AMR; the overall median proportion from studies in the review (1.0%) was used in the three other WHO regions. These proportions were applied to all countries in each respective WHO region except for the 51 countries free of *M. bovis* in cattle; the lowest observed proportion (0.3%) was assigned to the 51 countries free of *M. bovis* in cattle. Country level human tuberculosis incidence were abstracted from the WHO Global Tuberculosis Report [[4](#_ENREF_2_4)] and multiplied by population estimates and the proportion of human tuberculosis cases due to *M. bovis* to estimate human *M. bovis* cases. |
| --- | --- |
| CLINICAL OUTCOMES: | Clinical outcomes were *M. bovis* tuberculosis and *M. bovis* death. |
| DURATION: | *M. bovis* tuberculosis: we estimated duration using data in the 2014 WHO Global Tuberculosis Report on incidence and prevalence of human TB infections [[4](#_ENREF_2_4)]; these data yielded a duration of 1.5 years in all WHO regions except AFR where the duration was 1 year. |
| DISABILITY WEIGHT: | *M. bovis* tuberculosis: GBD 2010 disability weight of 0.331 (95% CI 0.222-0.450) for tuberculosis without HIV infection [[5](#_ENREF_2_5)]. |
| MORTALITY | We estimated *M. bovis* deaths following the same approach for estimating *M. bovis* cases after reducing the mortality by 20% due to the recognition from another FERG-commissioned review that *M. bovis* infections are more likely to result in extra pulmonary infections [[6](#_ENREF_2_6)] and extra pulmonary infections have a lower case-fatality ratio than pulmonary tuberculosis infections; a 20% reduction in mortality was based on a review of the US national surveillance data from 2009-2010 which found that the case fatality ratio (CFR) for extra pulmonary tuberculosis infections was approximately 20% lower than the CFR for pulmonary tuberculosis infections. Therefore, country level human tuberculosis mortality rates of tuberculosis among persons not infected with HIV were abstracted from the WHO Global Tuberculosis Report [[4](#_ENREF_2_4)], reduced by 20%, and then multiplied by population estimates and the proportion of human tuberculosis cases due to *M. bovis* to estimate *M. bovis* deaths. |
| AGE DISTRIBUTION | We assumed that the age distribution of *M. bovis* cases and deaths was the same as the age distribution of human tuberculosis cases and deaths and therefore used the age distribution from table 3.2 of the WHO Global Tuberculosis Report: 2% <15 years, 60% 15-44 years, 28% 45-64 years, 10% >65 years [[4](#_ENREF_2_4)]. |
| SEX DISTRIBUTION | We assumed that the sex distribution of *M. bovis* cases and deaths was the same as the sex distribution of human tuberculosis cases and deaths and therefore used the sex distribution from table 3.2 of the WHO Global Tuberculosis Report: 65% male [[4](#_ENREF_2_4)]. |

Reference

1. World Organisation for Animal Health Information Database (WAHID) at the World Organisation for Animal Health (OIE) [Internet]. [cited November 1, 2013]. Available from: <http://www.oie.int/wahis_2/public/wahid.php/Diseaseinformation/diseasehome>.

2. The European Union summary report on trends and sources of zoonoses, zoonotic agents and food-borne outbreaks in 2010. Euro Surveill. 2012;17.

3. Muller B, Durr S, Alonso S, Hattendorf J, Laisse CJ, Parsons SD, et al. Zoonotic Mycobacterium bovis-induced tuberculosis in humans. Emerg Infect Dis. 2013;19:899-908.

4. Organization WH. Global Tuberculosis Report 2012. WHO, Geneva, Switzerland. WHO/HTM/TB; 2012.

5. Salomon JA, Vos T, Hogan DR, Gagnon M, Naghavi M, Mokdad A, et al. Common values in assessing health outcomes from disease and injury: disability weights measurement study for the Global Burden of Disease Study 2010. Lancet. 2012;380:2129-2143.

6. Durr S, Muller B, Alonso S, Hattendorf J, Laisse CJ, van Helden PD, et al. Differences in primary sites of infection between zoonotic and human tuberculosis: results from a worldwide systematic review. PLoS Negl Trop Dis. 2013;7:e2399.

1. **Typhoid**

| INCIDENCE: | FERG reviewed available burden of disease estimates for typhoid fever [[1](#_ENREF_3_1), [2](#_ENREF_3_2)] before selecting the Institute of Health Metrics and Evaluation (IHME) Global Burden of Disease 2010 (GBD 2010) estimates because these estimates were published in the peer-reviewed literature and were available for all countries. At the request of FERG, IHME provided GBD 2010 data with country-specific, age-standardized prevalence (per 100,000 population) of "typhoid and paratyphoid fever", and "typhoid and paratyphoid liver abscesses and cysts" [[1](#_ENREF_3_1)]. Assuming a steady disease state, prevalence of typhoid and paratyphoid fever was converted to incidence by dividing by duration; similarly for typhoid and paratyphoid abscesses and cysts. Typhoid fever incidence was determined using a ratio of 1.0 *Salmonella* serotype Typhi cases to 0.23 *Salmonella* serotype Paratyphi A cases observed in national laboratory-based surveillance in the United States and in a global survey in 1997 [[3](#_ENREF_3_3)] ; similarly for typhoid abscesses and cysts. We used the GBD 2010 range of estimates around the mean estimate of global deaths due to typhoid and paratyphoid fevers (190,242 with 23,786 - 359,075) to derive a range of estimates for typhoid incidence. |
| --- | --- |
| CLINICAL OUTCOMES: | Clinical outcomes were typhoid fever, typhoid liver abscesses and cysts, and typhoid death [[1](#_ENREF_3_1)]. |
| DURATION: | Typhoid fever: duration 28 days (minimum duration 7 days - maximum 42 days). Typhoid liver abscesses and cysts: duration 42 days (minimum 28 days - maximum 56 days). Duration was estimated based on median duration before hospitalization for typhoid fever or typhoid abscesses/cysts of 10 days, recommendations for treatment duration for typhoid fever of 10-14 days and for typhoid abscesses/cysts of 28-112 days , and presumed longer duration in patients with typhoid fever or typhoid abscesses/cysts who are not hospitalized [[4](#_ENREF_3_4)]. |
| DISABILITY WEIGHT: | Typhoid fever: GBD 2010 disability weight of 0.210 (95% CI 0.139-0.298) for infectious disease, acute episode, severe. Typhoid liver abscesses and cysts: GBD 2010 disability weight of 0.254 (95% CI 0.170-0.355) for infectious disease, post-acute consequences, severe [[5](#_ENREF_3_5)]. |
| MORTALITY | GBD 2010 country-specific mortality data for "typhoid and paratyphoid fevers" were obtained by sex and 20 age groups from the IHME website [[6](#_ENREF_3_6)]. Typhoid mortality was determined using a ratio of 1.0 *Salmonella* serotype Typhi cases to 0.23 *Salmonella* serotype Paratyphi A cases observed in national laboratory-based surveillance in the United States and in a global survey in 1997 [[3](#_ENREF_3_3)]. We used the GBD 2010 range of estimates around the mean estimate of global deaths due to typhoid and paratyphoid fevers (190,242 with 23,786 - 359,075) to derive a range of estimates for paratyphoid deaths. |
| AGE DISTRIBUTION | Using data from IHME, the age distribution for typhoid fever, typhoid liver abscesses and cysts, and typhoid deaths was 5% <1 year, 16% 1-4 years, 22% 5-14 years, 19% 15-24 years, 14% 25-34 years, 9% 35-44 years, 6% 45-54 years, 3% 55-64 years, 3% 65-74 years, 1 % 75-84 years, 1% >85 years [[1](#_ENREF_3_1)]. |
| SEX DISTRIBUTION | Using data from IHME, the sex distribution for cases of typhoid fever, and typhoid liver abscesses and cysts was 56% male and the sex distribution for typhoid deaths was 58% male [[1](#_ENREF_3_1)]. |

References

1. Murray CJ, Ezzati M, Flaxman AD, Lim S, Lozano R, Michaud C, et al. GBD 2010: design, definitions, and metrics. Lancet. 2012;380:2063-2066.

2. Buckle GC, Walker CL, Black RE. Typhoid fever and paratyphoid fever: Systematic review to estimate global morbidity and mortality for 2010. J Glob Health. 2012;2:010401.

3. Crump JA, Luby SP, Mintz ED. The global burden of typhoid fever. Bulletin World Health Organization. 2004;82:346-353.

4. Butler T, Islam A, Kabir I, Jones PK. Patterns of morbidity and mortality in typhoid fever dependent on age and gender: review of 552 hospitalized patients with diarrhea. Reviews Infectious Diseases. 1991;13:85-90.

5. Salomon JA, Vos T, Hogan DR, Gagnon M, Naghavi M, Mokdad A, et al. Common values in assessing health outcomes from disease and injury: disability weights measurement study for the Global Burden of Disease Study 2010. Lancet. 2012;380:2129-2143.

6. Lozano R, Naghavi M, Foreman K, Lim S, Shibuya K, Aboyans V, et al. Global and regional mortality from 235 causes of death for 20 age groups in 1990 and 2010: a systematic analysis for the Global Burden of Disease Study 2010. Lancet. 2012;380:2095-2128.

1. **Paratyphoid**

| INCIDENCE: | FERG reviewed available burden of disease estimates for typhoid and paratyphoid fever [[1](#_ENREF_4_1), [2](#_ENREF_4_2)] before selecting the Institute of Health Metrics and Evaluation (IHME) Global Burden of Disease 2010 (GBD 2010) estimates because these estimates were published in the peer-reviewed literature and were available for all countries. At the request of FERG, IHME provided GBD 2010 data with country-specific, age-standardized prevalence (per 100,000 population) of "typhoid and paratyphoid fever", and "typhoid and paratyphoid liver abscesses and cysts" [[1](#_ENREF_4_1)]. Assuming a steady disease state, prevalence of typhoid and paratyphoid fever was converted to incidence by dividing by duration; similarly for typhoid and paratyphoid abscesses and cysts. Paratyphoid fever incidence was determined using a ratio of 0.23 *Salmonella* serotype Paratyphi A cases to 1.0 *Salmonella* serotype Typhi cases observed in national laboratory-based surveillance in the United States and in a global survey in 1997 [[3](#_ENREF_4_3)]; similarly for paratyphoid abscesses and cysts. We used the GBD 2010 range of estimates around the mean estimate of global deaths due to typhoid and paratyphoid fevers (190,242 with 23,786 - 359,075) to derive a range of estimates for paratyphoid incidence. |
| --- | --- |
| CLINICAL OUTCOMES: | Clinical outcomes were paratyphoid fever, paratyphoid liver abscesses and cysts, and paratyphoid deaths [[1](#_ENREF_4_1)]. |
| DURATION: | Paratyphoid fever: duration 28 days (minimum duration 7 days - maximum 42 days); paratyphoid liver abscesses and cysts: duration 42 days (minimum 28 days - maximum 56 days). Duration was estimated based on median duration before hospitalization for paratyphoid fever or paratyphoid abscesses/cysts of 10 days, recommendations treatment duration for paratyphoid fever of 10-14 days and for paratyphoid abscesses and cysts of 28-112 days, and presumed longer duration in patients with paratyphoid fever or paratyphoid abscesses/cysts who are not hospitalized [[4](#_ENREF_4_4)]. |
| DISABILITY WEIGHT: | Paratyphoid fever: GBD 2010 disability weight of 0.210 (95% CI 0.139-0.298) for infectious disease, acute episode, severe. Paratyphoid liver abscesses and cysts: GBD 2010 disability weight of 0.254 (95% CI 0.170-0.355) for infectious disease, post-acute consequences, severe [[5](#_ENREF_4_5)]. |
| MORTALITY | GBD 2010 country-specific mortality data for "typhoid and paratyphoid fevers" were obtained by sex and 20 age groups from the IHME website [[6](#_ENREF_4_6)]. Paratyphoid mortality was determined using a ratio of 0.23 *Salmonella* serotype Paratyphi A cases to 1.0 *Salmonella* serotype Typhi cases observed in national laboratory-based surveillance in the United States and in a global survey in 1997 [[4](#_ENREF_4_4)]. We used the GBD 2010 range of estimates around the mean estimate of global deaths due to typhoid and paratyphoid fevers (190,242 with 23,786 - 359,075) to derive a range of estimates for paratyphoid deaths. |
| AGE DISTRIBUTION | Using data from IHME, the age distribution for paratyphoid fever, paratyphoid liver abscesses and cysts, and paratyphoid deaths was 5% <1 year, 16% 1-4 years, 22% 5-14 years, 19% 15-24 years, 14% 25-34 years, 9% 35-44 years, 6% 45-54 years, 3% 55-64 years, 3% 65-74 years, 1 % 75-84 years, 1% >85 years [[1](#_ENREF_4_1)]. |
| SEX DISTRIBUTION | Using data from IHME, the sex distribution for cases of paratyphoid fever, and paratyphoid liver abscesses and cysts was 56% male and the sex distribution for paratyphoid deaths was 58% male [[1](#_ENREF_4_1)]. |

Reference

1. Buckle GC, Walker CL, Black RE. Typhoid fever and paratyphoid fever: Systematic review to estimate global morbidity and mortality for 2010. J Glob Health. 2012;2:010401.

2. Murray CJ, Ezzati M, Flaxman AD, Lim S, Lozano R, Michaud C, et al. GBD 2010: design, definitions, and metrics. Lancet. 2012;380:2063-2066.

3. Crump JA, Luby SP, Mintz ED. The global burden of typhoid fever. Bulletin World Health Organization. 2004;82:346-353.

4. Butler T, Islam A, Kabir I, Jones PK. Patterns of morbidity and mortality in typhoid fever dependent on age and gender: review of 552 hospitalized patients with diarrhea. Reviews Infectious Diseases. 1991;13:85-90.

5. Salomon JA, Vos T, Hogan DR, Gagnon M, Naghavi M, Mokdad A, et al. Common values in assessing health outcomes from disease and injury: disability weights measurement study for the Global Burden of Disease Study 2010. Lancet. 2012;380:2129-2143.

6. Lozano R, Naghavi M, Foreman K, Lim S, Shibuya K, Aboyans V, et al. Global and regional mortality from 235 causes of death for 20 age groups in 1990 and 2010: a systematic analysis for the Global Burden of Disease Study 2010. Lancet. 2012;380:2095-2128.

1. **Hepatitis A infection**

| INCIDENCE: | Assuming a case-fatality ratio of 0.2% [[1](#_ENREF_5_1)], the Institute of Health Metrics and Evaluation (IHME) Global Burden of Disease 2010 (GBD 2010) country-specific data on "hepatitis A", available on IHME website by sex and 20 age groups [[2](#_ENREF_5_2)] were converted to incidence. We used the GBD 2010 range of estimates around the mean estimate of global deaths due to hepatitis A (102,850 with 51,157 - 228,057) to derive a range of estimates for hepatitis A incidence. |
| --- | --- |
| CLINICAL OUTCOMES: | Clinical outcomes were acute hepatitis A (severe), acute hepatitis A (mild), and hepatitis A death. For acute hepatitis, we assumed 50% of cases were severe and 50% of cases were mild [[1](#_ENREF_5_1)]. |
| DURATION: | Acute hepatitis A: duration 21 days (minimum duration 14 days - maximum duration 30 days) [[1](#_ENREF_5_1)]. |
| DISABILITY WEIGHT: | Acute hepatitis A (severe): GBD 2010 disability weight of 0.210 (95% CI 0.139 - 0.298) for infectious disease, acute episode, severe. Acute hepatitis A (mild): GBD 2010 disability weight of 0.005 (95% CI 0.002 - 0.011) for infectious disease, acute episode, mild [[3](#_ENREF_5_3)]. |
| MORTALITY | GBD 2010 country-specific mortality data for "hepatitis A" were obtained by sex and 20 age groups from the IHME website [[2](#_ENREF_5_2)]. We used the GBD 2010 range of estimates around the mean estimate of global deaths due to hepatitis A (102,850 with 51,157 - 228,057) to derive a range of estimates for hepatitis A deaths. |
| AGE DISTRIBUTION | Using data from IHME, the age distribution for acute hepatitis A cases and hepatitis A deaths was 10% <1 year, 5% 1-4 years, 2% 5-14 years, 3% 15-24 years, 5% 25-34 years, 11% 35-44 years, 17% 45-54 years, 20% 55-64 years, 17% 65-74 years, 5 % 75-84 years, 5% >85 years [[2](#_ENREF_5_2)]. |
| SEX DISTRIBUTION | Using data from IHME, the sex distribution for acute hepatitis A cases and hepatitis A deaths was 57% male [[2](#_ENREF_5_2)]. |

Reference

1. European Center for Disease Control. Disease specific information: Hepatitis A virus. 2013.

2. Lozano R, Naghavi M, Foreman K, Lim S, Shibuya K, Aboyans V, et al. Global and regional mortality from 235 causes of death for 20 age groups in 1990 and 2010: a systematic analysis for the Global Burden of Disease Study 2010. Lancet. 2012;380:2095-2128.

3. Salomon JA, Vos T, Hogan DR, Gagnon M, Naghavi M, Mokdad A, et al. Common values in assessing health outcomes from disease and injury: disability weights measurement study for the Global Burden of Disease Study 2010. Lancet. 2012;380:2129-2143.

1. **Shiga toxin-producing *Escherichia coli* (STEC) infection**

| INCIDENCE: | Using a FERG-commissioned systematic review which screened 17,178 articles and a search for national surveillance data, Shiga toxin-producing *Escherichia coli* (STEC) incidence data from 21 countries were identified. Using a hierarchical study selection process, in the WHO subregions with prospective cohort studies or multipliers studies which estimated national STEC incidence, that STEC incidence was assigned to all countries in the subregion. In WHO subregions with only STEC notifiable disease data, STEC incidence for all countries in the subregion was estimated using a multiplier of 36 (range 7.4 - 106.8) to account for under-reporting; the STEC incidence from notifiable disease data was multiplied by the multiplier to estimate the national STEC incidence. In WHO subregions with no STEC incidence data, geographic proximity was used to extrapolate the STEC incidence to all countries in the subregion [[1](#_ENREF_6_1)]. These efforts led to the following regional incidence (per 100,000 population) estimates: AFR subregions D and E 1.4, AMR subregions A and D 93.5, AMR subregion B 27.2, EMR 152.6. EUR subregion A 47.1, EUR subregion B 2.7, EUR subregion C 2.5, SEAR 66.3, WPR subregion A 44.5, and WPR subregion B 3.5. |
| --- | --- |
| CLINICAL OUTCOMES: | Clinical outcomes of STEC infections were acute STEC diarrhea (severe), acute STEC diarrhea (moderate), acute STEC diarrhea (mild), STEC hemolytic uremic syndrome (HUS), STEC end stage renal disease (ESRD), and STEC death. We assumed that 2% of STEC infections resulted in severe diarrhea, 18% of STEC infections resulted in moderate diarrhea, and 80% of STEC infections resulted in mild diarrhea [[2](#_ENREF_6_2)]. We assumed that the following percent of STEC infections were serotype O157: 36% in AMR A, AMR B, EUR, and WPR A; 10% in AMR D, AFR and SEAR; and 0% in EMR. We assumed 0.8% (minimum 0.7% - maximum 0.9%) of O157 STEC infections and 0.03% (minimum 0.01% - maximum 0.04%) of non-O157 STEC infections resulted in HUS, and the 3% (minimum 0% - maximum 30%) of HUS cases resulted in ESRD [[1](#_ENREF_6_1)]. |
| DURATION: | Acute STEC diarrhea: duration 7 days (minimum 5 days - maximum 10 days) [[2](#_ENREF_6_2)]. STEC hemolytic uremic syndrome: duration 28 days (minimum 14 days - maximum 42 days). STEC end stage renal disease: results in lifelong disability in countries in AMR A, EUR A, and WPR A and death in other countries [[1](#_ENREF_6_1), [3](#_ENREF_6_3), [4](#_ENREF_6_4)]. |
| DISABILITY WEIGHT: | Acute diarrhea (severe): GBD 2010 disability weight of 0.281 (95% CI 0.184-0.399) for diarrhea, severe. Acute diarrhea (moderate): GBD 2010 disability weight of 0.202 (95% CI 0.133-0.299) for diarrhea, moderate. Acute diarrhea (mild): GBD 2010 disability weight of 0.061 (95% CI 0.036-0.093) for diarrhea, mild. STEC hemolytic uremic syndrome: GBD 2010 disability weight 0.210 (95% CI 0.139-0.298) for infectious disease, acute episode, severe. STEC end stage renal disease: GBD 2010 disability weight of 0.573 (95% CI 0.397-0.749) for end-stage renal disease, on dialysis [[5](#_ENREF_6_5)]. |
| MORTALITY | STEC hemolytic uremic syndrome case fatality ratio 3.7%. STEC end stage renal disease case fatality ratio 20% in countries in AMR A, EUR A, and WPR A; case fatality ratio 100% in other countries [[1](#_ENREF_6_1)]. |
| AGE DISTRIBUTION | Acute STEC diarrhea and STEC hemolytic uremic syndrome age (HUS) distribution: 29% <5 years, 20% 5-14 years, 35% 15-54 years, 16% >=55 years. STEC end stage renal disease (ESRD) and ESRD deaths age distribution: 41% <5 years, 18% 5-14 years, 26% 15-54 years, 15% >= 55 years. HUS deaths age distribution: 11% < 1 year, 47% 1-4 years, 14% 5-14 years, 22% 15-64 years, 6% >=65 years [[3](#_ENREF_6_3)]. |
| SEX DISTRIBUTION | Acute STEC diarrhea, STEC hemolytic uremic syndrome (HUS), STEC end stage renal disease (ESRD), HUS deaths, and ESRD deaths sex distribution: 50% male [[1](#_ENREF_6_1)]. |

Reference

1. Majowicz SE, Scallan E, Jones-Bitton A, Sargeant JM, Stapleton J, Angulo FJ, et al. Global Incidence of Human Shiga Toxin-Producing Escherichia coli Infections and Deaths: A Systematic Review and Knowledge Synthesis. Foodborne Pathog Dis. 2014;11:447-455.

2. Lamberti LM, Fischer Walker CL, Black RE. Systematic review of diarrhea duration and severity in children and adults in low- and middle-income countries. BMC Public Health. 2012;12:276.

3. Shiga/Vero toxin producing Escherichia coli infection (STEC/VTEC). [Internet]. 2013. Available from: <http://webcache.googleusercontent.com/search?q=cache:1k0NTdsWu8YJ:https://www.foodstandards.gov.au/publications/Documents/Shiga%2520toxin-producing%2520Escherichia%2520coli%2520%28STEC%29.doc+&cd=2&hl=en&ct=clnk&gl=au>.

4. Havelaar AH, Haagsma JA, Mangen MJ, Kemmeren JM, Verhoef LP, Vijgen SM, et al. Disease burden of foodborne pathogens in the Netherlands, 2009. Int J Food Microbiol. 2012;156:231-238.

5. Salomon JA, Vos T, Hogan DR, Gagnon M, Naghavi M, Mokdad A, et al. Common values in assessing health outcomes from disease and injury: disability weights measurement study for the Global Burden of Disease Study 2010. Lancet. 2012;380:2129-2143.

1. ***Clostridium botulinum* (Botulism)**

| INCIDENCE: | Estimates of incidence were only conducted for the 60 EUR and other WHO subregion A (low mortality) countries. Based on a literature review for articles with national estimates of foodborne diseases including botulism, we identified national estimates of the incidence of botulism from five countries: Canada [[1](#_ENREF_7_1)], France [[2](#_ENREF_7_2)], Georgia [[3](#_ENREF_7_3)], Poland [[4](#_ENREF_7_4)], and the United States [[5](#_ENREF_7_5)]. The median botulism incidence from these five countries was from Canada, therefore the botulism incidence from Canada (0.04 per 100,000 population with a 95% confidence interval of 0.02-0.08 per 100,000) was used as the incidence for all 56 countries in EUR and AMR A. |
| --- | --- |
| CLINICAL OUTCOMES: | Clinical outcomes were botulism (mild to moderate), botulism (severe), and botulism death. We assumed that 35% (range: 20%-50%) of botulism cases resulted in severe botulism [[3](#_ENREF_7_3), [4](#_ENREF_7_4), [6](#_ENREF_7_6)]. |
| DURATION: | Botulism (mild to moderate): duration 10 days (minimum duration 5 days - maximum duration 20 days); botulism (severe): duration 30 days (minimum duration 15 days - maximum duration 180 days) [[3](#_ENREF_7_3), [4](#_ENREF_7_4), [6](#_ENREF_7_6)]. |
| DISABILITY WEIGHT: | Botulism (mild to moderate): GBD 2010 disability weight 0.198 (95% CI 0.137 - 0.278) for multiple sclerosis, mild. Botulism (severe): GBD 2010 disability weight 0.445 (95% CI 0.303 - 0.593) for multiple sclerosis, moderate [[7](#_ENREF_7_7)]. |
| MORTALITY | Estimates of mortality were only conducted for the 56 countries in EUR and AMR A. Severe botulism case fatality ratio 15% (range: 5%-25%). Assume no deaths among mild to moderate botulism cases [[3-5](#_ENREF_7_3)]. |
| AGE DISTRIBUTION | Mild to moderate botulism, severe botulism, and botulism death age distribution: mode 50 years (minimum age 4 years - maximum age 88 years) [[3](#_ENREF_7_3), [4](#_ENREF_7_4), [6](#_ENREF_7_6)]. |
| SEX DISTRIBUTION | Mild to moderate botulism, severe botulism, and botulism death sex distribution: 48% male [[3](#_ENREF_7_3), [4](#_ENREF_7_4), [6](#_ENREF_7_6)]. |

Reference

1. Thomas MK, Murray R, Flockhart L, Pintar K, Pollari F, Fazil A, et al. Estimates of the burden of foodborne illness in Canada for 30 specified pathogens and unspecified agents, circa 2006. Foodborne Pathog Dis. 2013;10:639-648.

2. Vaillant V, de Valk H, Baron E, Ancelle T, Colin P, Delmas MC, et al. Foodborne infections in France. Foodborne Pathog Dis. 2005;2:221-232.

3. Varma JK, Katsitadze G, Moiscrafishvili M, Zardiashvili T, Chokheli M, Tarkhashvili N, et al. Foodborne botulism in the Republic of Georgia. Emerg Infect Dis. 2004;10:1601.

4. Czerwinski M, Czarkowski MP, Kondej B. [Botulism in Poland in 2010]. Przeglad Epidemiologiczny. 2012;66:267-271.

5. Scallan E, Griffin PM, Angulo FJ, Tauxe RV, Hoekstra RM. Foodborne illness acquired in the United States--unspecified agents. Emerg Infect Dis. 2011;17:16-22.

6. Sobel J, Tucker N, Sulka A, McLaughlin J, Maslanka S. Foodborne botulism in the United States, 1990-2000. Emerg Infects Dis. 2004;10:1606-1611.

7. Salomon JA, Vos T, Hogan DR, Gagnon M, Naghavi M, Mokdad A, et al. Common values in assessing health outcomes from disease and injury: disability weights measurement study for the Global Burden of Disease Study 2010. Lancet. 2012;380:2129-2143.

1. ***Clostridium* *perfringens* intoxication**

| INCIDENCE: | Estimates of incidence were only conducted for the 61 EUR and other WHO subregion A (low mortality) countries. Based on a literature review for articles with national estimates of foodborne diseases that included *Clostridium perfringens* intoxications*,* we identified national incidence estimates for *Clostridium perfringens* intoxications from seven countries: Australia[[1](#_ENREF_8_1)], Canada [[2](#_ENREF_8_2)], France [[3](#_ENREF_8_3)], Netherlands [[4](#_ENREF_8_4)], New Zealand [[5](#_ENREF_8_5)], United Kingdom [[6](#_ENREF_8_6)], and the United States [[7](#_ENREF_8_7)]. The median *C. perfringens* intoxication incidence from these seven countries was from the United States, therefore the *C. perfringens* intoxication incidence from the United States (324.19 per 100,000 population with a 95% confidence interval of 126.14 - 833.44 per 100,000) was used as the *C. perfringens* intoxication incidence for all EUR and other WHO subregion A countries. |
| --- | --- |
| CLINICAL OUTCOMES: | Clinical outcomes were acute gastroenteritis due to *C. perfringens* intoxication and death due to *C. perfringens* intoxication [[8](#_ENREF_8_8)]. |
| DURATION: | Acute gastroenteritis due to *C. perfringens* intoxication: duration 1 day (minimum duration 0.25 days - maximum duration 2.5 days) [[8](#_ENREF_8_8)]. |
| DISABILITY WEIGHT: | Acute gastroenteritis due to *C. perfringens* intoxication: GBD 2010 disability weight 0.061 (95% CI 0.036-0.093) for diarrhea, mild [[9](#_ENREF_8_9)]. |
| MORTALITY | Estimates of mortality were only conducted for the 61 EUR and other WHO subregion A (low mortality) countries. National estimates of *C. perfringens* intoxications cases and deaths were available from Australia [[1](#_ENREF_8_1)], France [[3](#_ENREF_8_3)], Netherlands [[4](#_ENREF_8_4)], New Zealand [[5](#_ENREF_8_5)], and the United States [[7](#_ENREF_8_7)]; the median case fatality ratio (CFR) from these five countries was the New Zealand (0.0030% [95%CI: 0.0024%-0.0038%]), therefore the CFR from New Zealand was used as the CFR for all EUR and other WHO subregion A countries. |
| AGE DISTRIBUTION | Acute gastroenteritis and deaths due to *C. perfringens* intoxication age distribution: 1% <5 years, 13% 5-14 years, 59% 15-54 years, 27% >=55 years [[8](#_ENREF_8_8)]. |
| SEX DISTRIBUTION | Acute gastroenteritis and deaths due to *C. perfringens* intoxication sex distribution: 63% male [[8](#_ENREF_8_8)]. |

Reference

1. Kirk M, Ford L, Glass K, Hall K. Foodborne illness, Australia, circa 2000 and circa 2010. Emerg Infect Dis. 2014;20:1857-1864.

2. Thomas MK, Murray R, Flockhart L, Pintar K, Pollari F, Fazil A, et al. Estimates of the burden of foodborne illness in Canada for 30 specified pathogens and unspecified agents, circa 2006. Foodborne Pathog Dis. 2013;10:639-648.

3. Vaillant V, de Valk H, Baron E, Ancelle T, Colin P, Delmas MC, et al. Foodborne infections in France. Foodborne Pathog Dis. 2005;2:221-232.

4. Havelaar AH, Haagsma JA, Mangen MJ, Kemmeren JM, Verhoef LP, Vijgen SM, et al. Disease burden of foodborne pathogens in the Netherlands, 2009. Int J Food Microbiol. 2012;156:231-238.

5. Cressey P, Lake R. Estimated incidence of foodborne illness in New Zealand: Application of overseas models and multipliers. Report—New Zealand Government, Christ Church, New Zealand. 2011.

6. Tam CC, Rodrigues LC, Viviani L, Dodds JP, Evans MR, Hunter PR, et al. Longitudinal study of infectious intestinal disease in the UK (IID2 study): incidence in the community and presenting to general practice. Gut. 2012;61:69-77.

7. Scallan E, Griffin PM, Angulo FJ, Tauxe RV, Hoekstra RM. Foodborne illness acquired in the United States--unspecified agents. Emerg Infect Dis. 2011;17:16-22.

8. Bennett SD, Walsh KA, Gould LH. Foodborne disease outbreaks caused by Bacillus cereus, Clostridium perfringens, and Staphylococcus aureus--United States, 1998-2008. Clin Infect Dis. 2013;57:425-433.

9. Salomon JA, Vos T, Hogan DR, Gagnon M, Naghavi M, Mokdad A, et al. Common values in assessing health outcomes from disease and injury: disability weights measurement study for the Global Burden of Disease Study 2010. Lancet. 2012;380:2129-2143.

1. ***Staphylococcus aureus* intoxication**

| INCIDENCE: | Estimates of incidence were only conducted for the 61 EUR and other WHO subregion A (low mortality) countries. Based on a literature review for articles with national estimates of foodborne diseases that included *Staphylococcus aureus* intoxication*,* we identified national incidence estimates for *S. aureus* intoxication from seven countries: Australia [[1](#_ENREF_9_1)], Canada [[2](#_ENREF_9_2)], France [[3](#_ENREF_9_3)], Netherlands [[4](#_ENREF_9_4)], New Zealand [[5](#_ENREF_9_5)], England and Wales as a proxy for United Kingdom [[6](#_ENREF_9_6)], and the United States [[7](#_ENREF_9_7)]. The median *S. aureus* intoxication incidence from these seven countries was from Canada, therefore the *S. aureus* intoxication incidence from the Canada (77.3 per 100,000 population with a 95% confidence interval of 50.65-118.0 per 100,000) was used as the *S. aureus* intoxication incidence for all EUR and other subregion A countries. |
| --- | --- |
| CLINICAL OUTCOMES: | Clinical outcomes were acute gastroenteritis due to *S. aureus* intoxication and death due to *S. aureus* intoxication [[8](#_ENREF_9_8)]. |
| DURATION: | Acute gastroenteritis due to *S. aureus* intoxication: duration 1 day (minimum duration 0.25 days - maximum duration 2.5 days) [[8](#_ENREF_9_8)]. |
| DISABILITY WEIGHT: | Acute gastroenteritis due to *S. aureus* intoxication: GBD 2010 disability weight 0.061 (95% CI 0.036-0.093) for diarrhea, mild [[9](#_ENREF_9_9)]. |
| MORTALITY | Estimates of mortality were only conducted for the 61 EUR and other WHO subregion A (low mortality) countries. National estimates of *S. aureus* intoxication cases and deaths were available from the Netherlands [[4](#_ENREF_9_4)] and the United States [[7](#_ENREF_9_7)]; the case fatality ratio (CFR) for the Netherlands was 0.0024% and for the United States was 0.0025%. We used the CFR from the United States as the CFR for all EUR and other subregion A countries, with a 95% confidence interval of 0.0012%-0.0045%. |
| AGE DISTRIBUTION | Acute gastroenteritis and deaths due to *S. aureus* intoxication age distribution: 5% <5 years, 19% 5-14 years, 48% 15-54 years, 28% >=55 years [[8](#_ENREF_9_8)]. |
| SEX DISTRIBUTION | Acute gastroenteritis and deaths due to *S. aureus* intoxication sex distribution: 48% male [[8](#_ENREF_9_8)]. |

Reference

1. Kirk M, Ford L, Glass K, Hall K. Foodborne illness, Australia, circa 2000 and circa 2010. Emerg Infect Dis. 2014;20:1857-1864.

2. Thomas MK, Murray R, Flockhart L, Pintar K, Pollari F, Fazil A, et al. Estimates of the burden of foodborne illness in Canada for 30 specified pathogens and unspecified agents, circa 2006. Foodborne Pathog Dis. 2013;10:639-648.

3. Vaillant V, de Valk H, Baron E, Ancelle T, Colin P, Delmas MC, et al. Foodborne infections in France. Foodborne Pathog Dis. 2005;2:221-232.

4. Havelaar AH, Haagsma JA, Mangen MJ, Kemmeren JM, Verhoef LP, Vijgen SM, et al. Disease burden of foodborne pathogens in the Netherlands, 2009. Int J Food Microbiol. 2012;156:231-238.

5. Cressey P, Lake R. Estimated incidence of foodborne illness in New Zealand: Application of overseas models and multipliers. Report—New Zealand Government, Christ Church, New Zealand. 2011.

6. Adak GK, Long SM, O'Brien SJ. Trends in indigenous foodborne disease and deaths, England and Wales: 1992 to 2000. Gut. 2002;51:832-841.

7. Scallan E, Griffin PM, Angulo FJ, Tauxe RV, Hoekstra RM. Foodborne illness acquired in the United States--unspecified agents. Emerg Infect Dis. 2011;17:16-22.

8. Bennett SD, Walsh KA, Gould LH. Foodborne disease outbreaks caused by Bacillus cereus, Clostridium perfringens, and Staphylococcus aureus--United States, 1998-2008. Clin Infect Dis. 2013;57:425-433.

9. Salomon JA, Vos T, Hogan DR, Gagnon M, Naghavi M, Mokdad A, et al. Common values in assessing health outcomes from disease and injury: disability weights measurement study for the Global Burden of Disease Study 2010. Lancet. 2012;380:2129-2143.

1. ***Bacillus cereus* intoxication**

| INCIDENCE: | Estimates of incidence were only conducted for the 61 EUR and other WHO subregion A (low mortality) countries. Based on a literature review for articles with national estimates of foodborne diseases that included *Bacillus cereus* intoxication*,* we identified national incidence estimates for *Bacillus cereus* intoxication from seven countries: Australia [[1](#_ENREF_10_1)], Canada [[2](#_ENREF_10_2)], France [[3](#_ENREF_10_3)], Netherlands [[4](#_ENREF_10_4)], New Zealand [[5](#_ENREF_10_5)], England and Wales as a proxy for United Kingdom [[6](#_ENREF_10_6)], and the United States [[7](#_ENREF_10_7)]. The median *B. cereus* intoxication incidence from these seven countries was for the United Kingdom (England and Wales), therefore the *B. cereus* intoxication incidence from the United Kingdom (21.4 per 100,000) was used as the *B. cereus* intoxication incidence for all EUR and other WHO subregion A countries; because the available *B. cereus* intoxication incidence estimate from England and Wales did not include a corresponding confidence interval, the average values of the intervals from the countries with the next lowest and next highest *B. cereus* intoxication incidence were used (United States 5.2-49.4 and the Netherlands 11.5-67.2) for a 95% confidence interval 7.9-58.3 per 100,000. |
| --- | --- |
| CLINICAL OUTCOMES: | Clinical outcomes were acute gastroenteritis due to *B. cereus* intoxication [[8](#_ENREF_10_8)]. |
| DURATION: | Acute gastroenteritis due to *B. cereus* intoxication: duration 1 day (minimum duration 0.25 days - maximum duration 2.5 days) [[8](#_ENREF_10_8)]. |
| DISABILITY WEIGHT: | Acute gastroenteritis due to *B. cereus* intoxication: GBD 2010 disability weight 0.061 (95% CI 0.036-0.093) for diarrhea, mild [[9](#_ENREF_10_9)]. |
| MORTALITY | No deaths estimated. |
| AGE DISTRIBUTION | Acute gastroenteritis due to *B. cereus* intoxication age distribution: 3% <5 years, 14% 5-14 years, 53% 15-54 years, 30% >=55 years [[8](#_ENREF_10_8)]. |
| SEX DISTRIBUTION | Acute gastroenteritis due to *B. cereus* intoxication sex distribution: 50% male [[8](#_ENREF_10_8)]. |

Reference

1. Kirk M, Ford L, Glass K, Hall K. Foodborne illness, Australia, circa 2000 and circa 2010. Emerg Infect Dis. 2014;20:1857-1864.

2. Thomas MK, Murray R, Flockhart L, Pintar K, Pollari F, Fazil A, et al. Estimates of the burden of foodborne illness in Canada for 30 specified pathogens and unspecified agents, circa 2006. Foodborne Pathog Dis. 2013;10:639-648.

3. Vaillant V, de Valk H, Baron E, Ancelle T, Colin P, Delmas MC, et al. Foodborne infections in France. Foodborne Pathog Dis. 2005;2:221-232.

4. Havelaar AH, Haagsma JA, Mangen MJ, Kemmeren JM, Verhoef LP, Vijgen SM, et al. Disease burden of foodborne pathogens in the Netherlands, 2009. Int J Food Microbiol. 2012;156:231-238.

5. Cressey P, Lake R. Estimated incidence of foodborne illness in New Zealand: Application of overseas models and multipliers. Report—New Zealand Government, Christ Church, New Zealand. 2011.

6. Adak GK, Long SM, O'Brien SJ. Trends in indigenous foodborne disease and deaths, England and Wales: 1992 to 2000. Gut. 2002;51:832-841.

7. Scallan E, Griffin PM, Angulo FJ, Tauxe RV, Hoekstra RM. Foodborne illness acquired in the United States--unspecified agents. Emerg Infect Dis. 2011;17:16-22.

8. Bennett SD, Walsh KA, Gould LH. Foodborne disease outbreaks caused by Bacillus cereus, Clostridium perfringens, and Staphylococcus aureus--United States, 1998-2008. Clin Infect Dis. 2013;57:425-433.

9. Salomon JA, Vos T, Hogan DR, Gagnon M, Naghavi M, Mokdad A, et al. Common values in assessing health outcomes from disease and injury: disability weights measurement study for the Global Burden of Disease Study 2010. Lancet. 2012;380:2129-2143.

1. **Listeriosis**

| INCIDENCE: | Using a FERG-commissioned systematic review which screened 11,722 papers and national surveillance, listeriosis incidence data were extracted from 43 papers. National listeriosis incidence estimates were then calculated for all countries using the extracted data and imputed estimates through a multilevel random effects model [[1](#_ENREF_11_1)]. |
| --- | --- |
| CLINICAL OUTCOMES: | Clinical outcomes were determined using outcome probabilities from the FERG-commissioned review and a random effects meta-regression model; for each study identified in the review, a weight was assigned reflecting the study quality. These weights were included as a fixed effect in the meta-regression model. Clinical outcomes included perinatal and non-perinatal listerosis; we estimated that 79.3% (minimum 77.3% - maximum 81.3%) of listeriosis cases were perinatal and 20.7% (minimum 19.0% - maximum 22.4%) were non-perinatal. Clinical outcomes among perinatal listerosis cases were neonatal septicemia, neonatal meningitis, neurological sequelae, stillborn, and death; stillborn neonates were estimated but not included in the final FERG estimates of deaths and DALYS. We estimated 30.7% of perinatal listeriosis cases developed neonatal septicemia and 15.2% (minimum 13.1% - maximum 17.3%) neonatal meningitis, of whom 43.8% (minimum 31.8% - maximum 55.8%) had neurological sequelae. Clinical outcomes among non-perinatal listeriosis cases were septicemia, meningitis, neurological sequelae, and death; we estimated 61.6% (minimum 59.4% - maximum 63.8%) of non-perinatal listeriosis cases developed septicemia and 30.7% (minimum 28.7% - maximum 32.7%) meningitis, of whom 13.7% (minimum 8.2% - maximum 19.2%) had neurological sequelae [[1](#_ENREF_11_1)]. |
| DURATION: | For perinatal and non-perinatal listeriosis cases: septicemia duration 7 days, meningitis duration 182 days, and neurological sequelae 7 years [[1](#_ENREF_11_1)]. |
| DISABILITY WEIGHT: | For listeriosis septicemia: GBD 2010 disability weight (DW) of 0.210 (95% CI 0.139-0.298) for infectious disease, acute episode, severe [[2](#_ENREF_11_2)]. For listeriosis meningitis: a DW of 0.426 (95%CI 0.368 - 0.474) derived from multiplicative methodology and expert elicitation (with bootstrap analysis for CI) using combination of following DWs - (1) 0.210 for infectious disease, acute episode, severe (2) 0.126 for intellectual disability, severe, (3) average of 0.488 for epilepsy, severe and epilepsy, treated with recent seizures, and (4) 0.76 for motor impairment, moderate. For listeriosis neurological sequelae: a DW of 0.292 (95% CI 0.272-0.316) derived from a multiplicative methodology and expert elicitation (with bootstrap analysis for CI) using combination of following DWs - (1) 0.047 resulting from average of all 10 DWs involving hearing loss, (2) 0.087 resulting from average of all 5 DWs for vision loss, and (3) 0.303 resulting from average of all 4 DWs for stroke, long-term consequence [[1](#_ENREF_11_1), [2](#_ENREF_11_2)]. |
| MORTALITY | Listeriosis case fatality ratios were estimated following the same approach for estimating clinical outcomes of listeriosis cases; using probabilities from the FERG-commissioned review and a random effects meta-regression model. For each study identified in the review, a weight was assigned reflecting the study quality; these weights were included as a fixed effect in the meta-regression model. The case fatality ratio for perinatal cases was 14.9% (minimum 11.3% - maximum 18.5%); 9.2% (minimum 7.5% - maximum 10.9%) resulted in neonatal deaths and 5.7% (minimum 3.8% - maximum 7.6%) resulted in stillbirths; stillborn neonates were not included in the final FERG estimates of deaths and DALYs. The case fatality ratio for non-perinatal cases was 25.9% (minimum 23.8% - maximum 29.0%). |
| AGE DISTRIBUTION | The age distribution of listeriosis cases and deaths was determined from published papers during the FERG-commissioned review [[1](#_ENREF_11_1)]. The age distribution for perinatal listeriosis cases and deaths was: 100% <1 month. The age distribution for non-perinatal cases and deaths was: 0% <1 year, 2% 1-4 years, 4% 5-14 years, 10% 15-34 years, 6% 35-44 years, 7% 45-54 years, 13% 55-64 years, 20% 65-74 years, 20% 75-84 years, 18%>=85 years. |
| SEX DISTRIBUTION | The sex distribution of listeriosis cases and deaths was determined from published papers during the FERG-commissioned review [[1](#_ENREF_11_1)]. The sex distribution for listeriosis cases and deaths was: 50% male. |

Reference

1. Maertens-de-Noordhout1 C, Devleesschauwer B, Angulo FJ, Verbeke G, Haagsma J, Kirk M, et al. The global burden of listeriosis: a systematic review and meta-analysis. Lancet Infect Dis. 2014;14:1073-1082.

2. Salomon JA, Vos T, Hogan DR, Gagnon M, Naghavi M, Mokdad A, et al. Common values in assessing health outcomes from disease and injury: disability weights measurement study for the Global Burden of Disease Study 2010. Lancet. 2012;380:2129-2143.

1. **Nontyphoidal *Salmonella* infection**

| INCIDENCE: | The incidence of diarrheal non-typhoidal *Salmonella* (NTS) was estimated separately for middle/high mortality countries, and low mortality countries. For the 133 middle/high mortality countries, we used a modification of the Child Health Epidemiology Reference Group (CHERG) approach [[1](#_ENREF_12_1)]. To derive "envelopes" of diarrhea cases, for children <5 years of age we used estimates of diarrhea incidence from a CHERG systematic review [[2](#_ENREF_12_2)] and for persons >5 years of age we used a FERG-commissioned systematic review [[3](#_ENREF_12_3)]. We then estimated the etiological proportions of diarrheal illnesses due to NTS and the 10 other diarrheal pathogens* in children <5 years of age using a CHERG and FERG systematic review of etiology studies among outpatients and persons in the community, and the etiological proportion of diarrheal illnesses due to NTS and the other 10 diarrheal pathogens in persons >5 years of age using an updated FERG systematic review of etiology studies among inpatients, outpatients and persons in the community [[4](#_ENREF_12_4)]. The NTS etiological proportions were extracted from studies, and regional median NTS etiological proportions calculated. We modified the CHERG approach by dropping regional median NTS etiological proportion outliers that were >5 times greater than the global median NTS etiological proportion, and replacing missing regional NTS etiological proportions with the global median. Furthermore, for children <5 years of age, we proportionally decreased the etiological proportions for all 11 diarrheal pathogens in each region so that the sum of the etiological proportions for all diarrheal pathogens in a region equaled 1. The resultant regional NTS etiological proportions were multiplied by the regional estimates of diarrhea incidence, and the resultant regional NTS incidence was applied to all countries in that WHO region. In the 61 low mortality countries (EUR and other WHO subregion "A" countries), we used a literature review that identified national incidence estimates for NTS from seven countries: Australia [[5](#_ENREF_12_5)], Canada [[6](#_ENREF_12_6)], France [[7](#_ENREF_12_7)], Netherlands [[8](#_ENREF_12_8)], New Zealand [[9](#_ENREF_12_9)], United Kingdom [[10](#_ENREF_12_10)], and the United States [[11](#_ENREF_12_11)]. These national estimates were based on systematic reviews, national surveillance data, and expert judgment. In these seven countries, we used the estimated national NTS incidence (and range) for that country. For low mortality countries without a national estimate, we used the median NTS incidence from the seven national studies. The median incidence was from Australia; 301.5 per 100,000 population (which was increased by 19% to account for travelers using proxy information from New Zealand) with range 171.1 - 541.8. |
| --- | --- |
| CLINICAL OUTCOMES: | Clinical outcomes were acute non-typhoidal *Salmonella* (NTS) diarrhea (severe), acute NTS diarrhea (moderate), acute NTS diarrhea (mild), and NTS death. We assumed that 2% of NTS diarrheal cases resulted in severe diarrhea, 25% of NTS diarrheal cases resulted in moderate diarrhea, and 73% of NTS diarrheal cases resulted in mild diarrhea. |
| DURATION: | In children <5 years of age, duration of severe diarrhea was 8.4 days, moderate diarrhea was 6.4 days, and mild diarrhea was 4.3 days [[12](#_ENREF_12_12)]. Based on the assumed distribution of severe, moderate and mild diarrhea cases, the duration of all non-typhoidal *Salmonella* (NTS) diarrhea cases in children <5 years of age was estimated to be 4.9 days (minimum 4.3 days - maximum 8.4 days). In persons >5 years of age, the duration of NTS diarrhea was 2.8 days [[12](#_ENREF_12_12)]. |
| DISABILITY WEIGHT: | Acute non-typhoidal *Salmonella* (NTS) diarrhea (severe): GBD 2010 disability weight of 0.281 (95% CI 0.184-0.399) for diarrhea, severe. Acute NTS diarrhea (moderate): GBD 2010 disability weight of 0.202 (95% CI 0.133-0.299) for diarrhea, moderate. Acute NTS diarrhea (mild): GBD 2010 disability weight of 0.061 (95% CI 0.036-0.093) for diarrhea, mild. [[13](#_ENREF_12_13)] |
| MORTALITY: | The mortality of NTS was estimated separately for middle/high mortality countries, and low mortality countries. For the 133 middle/high mortality countries, we used a modification of the CHERG approach [[1](#_ENREF_12_1)]. We received envelopes of diarrheal deaths from WHO; because this estimate was not available with an uncertainty interval, we used the uncertainty range from the GBD 2010 estimate of diarrheal deaths (81.7% to 114.6% around the point estimate) [[14](#_ENREF_12_14)]. We then estimated the etiological proportions of diarrheal deaths due to NTS and the other 10 diarrheal pathogens* in children <5 years of age using a CHERG and FERG systematic review of etiology studies among inpatients, and the etiological proportions of diarrheal deaths due to NTS and the other 10 diarrheal pathogens in persons >5 years of age using an updated FERG systematic tic review of etiology studies among inpatients [[4](#_ENREF_12_4)]. The NTS etiological proportions were extracted from studies, and regional median NTS etiological proportions calculated. We modified the CHERG approach by dropping regional median NTS etiological proportion outliers that were >5 times greater than the global median NTS etiological proportion, and replacing missing regional NTS etiological proportions with the global median. Furthermore, for children <5 years of age, we proportionally decreased the etiological proportions for all 11 diarrheal pathogens in each region so that the sum of the etiological proportions for all diarrheal pathogens in a region equaled 1. The resultant regional NTS etiological proportions were multiplied by the regional estimates of diarrhea deaths, and the resultant regional NTS mortality was applied to all countries in that WHO region. In the 61 low mortality countries (EUR and other WHO subregion "A" countries), we used a literature review that identified NTS mortality estimates from five countries: Australia [[5](#_ENREF_12_5)], France [[7](#_ENREF_12_7)], Netherlands [[8](#_ENREF_12_8)], New Zealand [[9](#_ENREF_12_9)], and the United States [[11](#_ENREF_12_11)]. These national estimates were based on systematic reviews, national surveillance data, and expert judgment. In these five countries, we used the estimated national NTS mortality (and range) for that country. For low mortality countries without a national estimate, we used the median NTS mortality from the five national studies. The median NTS mortality was from the United States; 0.15 per 100,000 population: range 0 08- 0.40. |
| AGE DISTRIBUTION: | In middle/high mortality countries we estimated incidence and mortality of non-typhoidal *Salmonella* (NTS) diarrhea separately for children <5 years of age and persons >5 years of age. In low mortality countries the age distribution for NTS diarrhea cases was 24% <5 years, 10% 5-14 years, 11% 15-24 years, 42% 25-64 years, and 13% >65 years [[15](#_ENREF_12_15)]. |
| SEX DISTRIBUTION: | *Salmonella* sex distribution: 50% male. |
| * 11 diarrheal pathogens are: nontyphoidal *Salmonella*, *Campylobacter*, *Shigella*, norovirus, enterotoxigenic *E. coli* (ETEC), enteropathogenic *E. coli* (EPEC), *C*ryptosporidia*, Giardia*, *Entamoeba histolytica*, other diarrheal agents not known to be foodborne (rotavirus and astrovirus), and unspecified agents. | |

Reference

1. Lanata CF, Fischer-Walker CL, Olascoaga AC, Torres CX, Aryee MJ, Black RE, et al. Global causes of diarrheal disease mortality in children <5 years of age: a systematic review. PLOS One. 2013;8:e72788.

2. Walker CL, Rudan I, Liu L, Nair H, Theodoratou E, Bhutta ZA, et al. Global burden of childhood pneumonia and diarrhoea. Lancet. 2013;381:1405-1416.

3. Walker CL, Black RE. Diarrhoea morbidity and mortality in older children, adolescents, and adults. Epidemiol Infect. 2010;138:1215-1226.

4. Fischer Walker CL, Sack D, Black RE. Etiology of diarrhea in older children, adolescents and adults: a systematic review. PLOS Negl Trop Dis. 2010;4:e768.

5. Kirk M, Ford L, Glass K, Hall K. Foodborne illness, Australia, circa 2000 and circa 2010. Emerg Infect Dis. 2014;20:1857-1864.

6. Thomas MK, Murray R, Flockhart L, Pintar K, Pollari F, Fazil A, et al. Estimates of the burden of foodborne illness in Canada for 30 specified pathogens and unspecified agents, circa 2006. Foodborne Pathog Dis. 2013;10:639-648.

7. Vaillant V, de Valk H, Baron E, Ancelle T, Colin P, Delmas MC, et al. Foodborne infections in France. Foodborne Pathog Dis. 2005;2:221-232.

8. Havelaar AH, Haagsma JA, Mangen MJ, Kemmeren JM, Verhoef LP, Vijgen SM, et al. Disease burden of foodborne pathogens in the Netherlands, 2009. Int J Food Microbiol. 2012;156:231-238.

9. Cressey P, Lake R. Estimated incidence of foodborne illness in New Zealand: Application of overseas models and multipliers. Report—New Zealand Government, Christ Church, New Zealand. 2011.

10. Tam CC, Rodrigues LC, Viviani L, Dodds JP, Evans MR, Hunter PR, et al. Longitudinal study of infectious intestinal disease in the UK (IID2 study): incidence in the community and presenting to general practice. Gut. 2012;61:69-77.

11. Scallan E, Griffin PM, Angulo FJ, Tauxe RV, Hoekstra RM. Foodborne illness acquired in the United States--unspecified agents. Emerg Infect Dis. 2011;17:16-22.

12. Lamberti LM, Fischer Walker CL, Black RE. Systematic review of diarrhea duration and severity in children and adults in low- and middle-income countries. BMC Public Health. 2012;12:276.

13. Salomon JA, Vos T, Hogan DR, Gagnon M, Naghavi M, Mokdad A, et al. Common values in assessing health outcomes from disease and injury: disability weights measurement study for the Global Burden of Disease Study 2010. Lancet. 2012;380:2129-2143.

14. Lozano R, Naghavi M, Foreman K, Lim S, Shibuya K, Aboyans V, et al. Global and regional mortality from 235 causes of death for 20 age groups in 1990 and 2010: a systematic analysis for the Global Burden of Disease Study 2010. Lancet. 2012;380:2095-2128.

15. Foodborne Diseases Active Surveillance Network (FoodNet) [Internet]. Available from: <http://www.cdc.gov/foodnet/data/trends/tables>.

1. **Invasive Nontyphoidal *Salmonella* (iNTS) infection**

| INCIDENCE | Rates of iNTS are highly correlated with HIV prevalence and malaria risk [[1](#_ENREF_13_1)]. To estimate iNTS incidence globally, we used age-specific estimates of incidence from a FERG commissioned systematic review [[1](#_ENREF_13_1)] to constructed a random effect log linear model using covariates of country specific HIV and malaria deaths, and the log of Gross Domestic Product. As data were sparse, we predicted incidence for all ages, which was converted to age-specific incidence based on age profiles for iNTS cases in low and high incidence settings [[1](#_ENREF_13_1)]. From this, we predicted iNTS incidence among persons not infected with HIV [[2](#_ENREF_13_2), [3](#_ENREF_13_3)]. To estimate deaths, we assumed that the CFR for iNTS in non-HIV infected individuals was a uniform distribution with a most likely value of 10% (range 5–20%) in WHO sub-region B-E countries and a most likely value of 4.3% (range 3.9–6.6%) in WHO sub-region A countries [[4](#_ENREF_13_4)]. |
| --- | --- |
| CLINICAL OUTCOMES: | Clinical outcomes were invasive *Salmonella* infection and death. |
| DURATION: | The duration of invasive NTS infection was assumed to be the same as the duration of typhoid which was estimated to be 28 days (minimum duration 7 days - maximum duration 56 days). |
| DISABILITY WEIGHT: | Invasive NTS infection: GBD 2010 disability weight of 0.210 (95% CI 0.139-0.298) for infectious disease, acute episode, severe [[5](#_ENREF_13_5)]. |
| MORTALITY: | To estimate deaths, we assumed that the CFR for iNTS in non-HIV infected individuals was a uniform distribution with a most likely value of 10% (range 5–20%) in WHO sub-region B-E countries and a most likely value of 4.3% (range 3.9–6.6%) in WHO sub-region A countries [[6](#_ENREF_13_6)]. |
| AGE DISTRIBUTION: | We assessed the age distribution of invasive NTS cases and deaths in high (Mali) and low (United States) burden settings. |
| SEX DISTRIBUTION: | *Salmonella* sex distribution: 50% male. |

Reference

1. Ao T, Feasy N, Gordon M, Keddy K, Angulo F, Crump J. Global burden of invasive nontyphoidal Salmonella disease, 2010. Emerg Infect Dis. 2015;21:941-949.

2. Biggs HM, Lester R, Nadjm B, Mtove G, Todd JE, Kinabo GD, et al. Invasive Salmonella infections in areas of high and low malaria transmission intensity in Tanzania. Clin Infect Dis. 2014;58:638-647.

3. Mtove G, Amos B, von Seidlein L, Hendriksen I, Mwambuli A, Kimera J, et al. Invasive salmonellosis among children admitted to a rural Tanzanian hospital and a comparison with previous studies. PLOS One. 2010;5:e9244.

4. Koch K, al. e. International travel and the risk of hospitalization with non-typhoidal Salmonella bacteremia. A Danish population-based cohort study, 1999-2008. . BMC Infect Dis. 2011;11.

5. Organization WH. WHO methods and data sources for global burden of disease estimates 2000-2011. Global Health Estimates Technical Paper WHO/HIS/HSI/GHE. 2013.

6. Organization WH. Global health risks: mortality and burden of disease attributable to selected major risks: World Health Organization; 2009.

1. ***Campylobacter* infection**

| INCIDENCE: | The incidence of diarrheal *Campylobacter* was estimated separately for middle/high mortality countries, and low mortality countries. For the 133 middle/high mortality countries, we used a modification of the Child Health Epidemiology Reference Group (CHERG) approach [[1](#_ENREF_14_1)]. To derive "envelopes" of diarrhea cases, for children <5 years of age we used estimates of diarrhea incidence from a CHERG systematic review [[2](#_ENREF_14_2)] and for persons >5 years of age we used a FERG-commissioned systematic review [[3](#_ENREF_14_3)]. We then estimated the etiological proportions of diarrheal illnesses due to *Campylobacter* and the 10 other diarrheal pathogens* in children <5 years of age using a CHERG and FERG systematic review of etiology studies among outpatients and persons in the community and the etiological proportions of diarrheal illnesses due to *Campylobacter* and the 10 other diarrheal pathogens in persons >5 years of age using an updated FERG systematic review of etiology studies among inpatients, outpatients and persons in the community [[4](#_ENREF_14_4)]. The *Campylobacter* etiological proportions were extracted from studies, and regional median *Campylobacter* etiological proportions calculated. We modified the CHERG approach by dropping regional median *Campylobacter* etiological proportion outliers that were >5 times greater than the global median *Campylobacter* etiological proportion, and replacing missing regional *Campylobacter* etiological proportions with the global median. Furthermore, for children <5 years of age, we proportionally decreased the etiological proportions for all 11 diarrheal pathogens in each region so that the sum of the etiological proportions for all 11 diarrheal pathogens in a region equaled 1. The resultant regional *Campylobacter* etiological proportions were multiplied by the regional estimates of diarrhea incidence, and the resultant regional *Campylobacter* incidence was applied to all countries in that WHO region.  In the 61 low mortality countries (EUR and other WHO subregion "A" countries), we used a literature review that identified national incidence estimates for Campylobacter from seven countries: Australia [[5](#_ENREF_14_5)], Canada [[6](#_ENREF_14_6)], France [[7](#_ENREF_14_7)], Netherlands [[8](#_ENREF_14_8)], New Zealand [[9](#_ENREF_14_9)], United Kingdom [[10](#_ENREF_14_10)], and the United States [[11](#_ENREF_14_11)]. These national estimates were based on systematic reviews, national surveillance data, and expert judgment. In these seven countries, we used the estimated national *Campylobacter* incidence (and range) for that country. For low mortality countries without a national estimate, we used the median *Campylobacter* incidence from the seven national studies. The median incidence was from Canada; 789.2 per 100,000 population (after increasing by 20% to account for travelers according to proxy information from the United States) with range of 532.8 - 1,140.3. Using a systematic review that identified 63 papers, updated for papers published through 2013 for FERG by the author with the addition of 9 papers, the incidence of Guillain–Barré Syndrome (GBS) in all countries was estimated at 1.4 per 100,000 population (minimum 1.1 - maximum 1.8) [[12](#_ENREF_14_12)]. Based on a systematic review, we assumed that 31% (minimum 28% - maximum 45%) of GBS cases were due to *Campylobacter* infection [[13](#_ENREF_14_13)]. |
| --- | --- |
| CLINICAL OUTCOMES: | Clinical outcomes were acute *Campylobacter* diarrhea (severe), acute *Campylobacter* diarrhea (moderate), acute *Campylobacter* diarrhea (mild), GBS due to *Campylobacter* infection, and *Campylobacter* death. We assumed that 2% of *Campylobacter* diarrheal cases resulted in severe diarrhea, 25% of *Campylobacter* diarrheal cases resulted in moderate diarrhea, and 73% of *Campylobacter* diarrheal cases resulted in mild diarrhea. |
| DURATION: | In children <5 years of age, duration of severe diarrhea was 8.4 days, moderate diarrhea was 6.4 days, and mild diarrhea was 4.3 days [[14](#_ENREF_14_14)]. Based on the assumed distribution of severe, moderate and mild diarrhea cases, the duration of all *Campylobacter* diarrhea cases in children <5 years of age was estimated to be 4.9 days (minimum 4.3 days - maximum 8.4 days). In persons >5 years of age, the duration of *Campylobacter* diarrhea was 2.8 days [[14](#_ENREF_14_14)]. The duration of GBS due to *Campylobacter* infection was assumed life-long [[15](#_ENREF_14_15)]. |
| DISABILITY WEIGHT: | Acute *Campylobacter* diarrhea (severe): GBD 2010 disability weight of 0.281 (95% CI 0.184-0.399) for diarrhea, severe. Acute *Campylobacter* diarrhea (moderate): GBD 2010 disability weight of 0.202 (95% CI 0.133-0.299) for diarrhea, moderate. Acute *Campylobacter* diarrhea (mild): GBD 2010 disability weight of 0.061 (95% CI 0.036-0.093) for diarrhea, mild. GBS due to *Campylobacter* infection: GBD201 disability weight of 0.445 (95% CI 0.303 - 0.593) for multiple sclerosis, moderate [[16](#_ENREF_14_16)]. |
| MORTALITY: | The mortality of *Campylobacter* was estimated separately for middle/high mortality countries, and low mortality countries. For the 133 middle/high mortality countries, we used a modification of the CHERG approach [[1](#_ENREF_14_1)]. We received envelopes of diarrheal deaths from WHO; because this estimate was not available with an uncertainty interval, we used the uncertainty range from the GBD 2010 estimate of diarrheal deaths (81.7% to 114.6% around the point estimate) [[17](#_ENREF_14_17)]. We then estimated the etiological proportions of diarrheal deaths due to *Campylobacter* and the 10 other diarrheal pathogens in children <5 years of age using a CHERG and FERG systematic review of etiology studies among inpatients, and the etiological proportions of diarrheal deaths due to *Campylobacter* and the 10 other diarrheal pathogens in persons >5 years of age using an updated FERG systematic review of etiology studies among inpatients [[4](#_ENREF_14_4)]. The *Campylobacter* etiological proportions were extracted from studies, and regional median *Campylobacter* etiological proportions calculated. We modified the CHERG approach by dropping regional median *Campylobacter* etiological proportion outliers that were >5 times greater than the global median *Campylobacter* etiological proportion, and replacing missing regional *Campylobacter* etiological proportions with the global median. Furthermore, for children <5 years of age, we proportionally decreased the etiological proportions for all 11 diarrheal pathogens in each region so that the sum of the etiological proportions for all diarrheal pathogens in a region equaled 1. The resultant regional *Campylobacter* etiological proportions were multiplied by the regional estimates of diarrhea deaths, and the resultant regional *Campylobacter* mortality was applied to all countries in that WHO region. In the 61 low mortality countries (EUR and other WHO subregion "A" countries), we used a literature review that identified *Campylobacter* mortality estimates from five countries: Australia [[5](#_ENREF_14_5)], France [[7](#_ENREF_14_7)], Netherlands [[8](#_ENREF_14_8)], New Zealand [[9](#_ENREF_14_9)], and the United States [[11](#_ENREF_14_11)]. These national estimates were based on systematic reviews, national surveillance data, and expert judgment. In these five countries, we used the estimated national *Campylobacter* mortality (and range) for that country. For low mortality countries without a national estimate, we used the median *Campylobacter* mortality from the five national studies. The median *Campylobacter* mortality was the mean from the United States; 0.04 per 100,000 population with range 0 - 0.17. We assumed that the case fatality ratio for GBS due to *Campylobacter* infection was 4.1% (minimum 2.4%- maximum 6%) [[15](#_ENREF_14_15)]. |
| AGE DISTRIBUTION: | In middle/high mortality countries we estimated incidence and mortality of *Campylobacter* diarrhea separately for children <5 years of age and persons >5 years of age. In low mortality countries the age distribution for *Campylobacter* diarrhea cases was 11% <5 years, 8% 5-14 years, 10% 15-24 years, 57% 25-64 years, and 14% >65 years [[18](#_ENREF_14_18)]. We assumed the age distribution of *Campylobacter* associated GBS cases and deaths were the same as *Campylobacter* diarrhea cases and deaths. |
| SEX DISTRIBUTION: | *Campylobacter* sex distribution: 50% male. |
| * 11 diarrheal pathogens are: nontyphoidal *Salmonella*, *Campylobacter*, *Shigella*, norovirus, enterotoxigenic *E. coli* (ETEC), enteropathogenic *E. coli* (EPEC), *C*ryptosporidia*, Giardia*, *Entamoeba histolytica*, other diarrheal agents not known to be foodborne (rotavirus and astrovirus), and unspecified agents. | |

Reference

1. Lanata CF, Fischer-Walker CL, Olascoaga AC, Torres CX, Aryee MJ, Black RE, et al. Global causes of diarrheal disease mortality in children <5 years of age: a systematic review. PLOS One. 2013;8:e72788.

2. Walker CL, Rudan I, Liu L, Nair H, Theodoratou E, Bhutta ZA, et al. Global burden of childhood pneumonia and diarrhoea. Lancet. 2013;381:1405-1416.

3. Walker CL, Black RE. Diarrhoea morbidity and mortality in older children, adolescents, and adults. Epidemiol Infect. 2010;138:1215-1226.

4. Fischer Walker CL, Sack D, Black RE. Etiology of diarrhea in older children, adolescents and adults: a systematic review. PLOS Negl Trop Dis. 2010;4:e768.

5. Kirk M, Ford L, Glass K, Hall K. Foodborne illness, Australia, circa 2000 and circa 2010. Emerg Infect Dis. 2014;20:1857-1864.

6. Thomas MK, Murray R, Flockhart L, Pintar K, Pollari F, Fazil A, et al. Estimates of the burden of foodborne illness in Canada for 30 specified pathogens and unspecified agents, circa 2006. Foodborne Pathog Dis. 2013;10:639-648.

7. Vaillant V, de Valk H, Baron E, Ancelle T, Colin P, Delmas MC, et al. Foodborne infections in France. Foodborne Pathog Dis. 2005;2:221-232.

8. Havelaar AH, Haagsma JA, Mangen MJ, Kemmeren JM, Verhoef LP, Vijgen SM, et al. Disease burden of foodborne pathogens in the Netherlands, 2009. Int J Food Microbiol. 2012;156:231-238.

9. Cressey P, Lake R. Estimated incidence of foodborne illness in New Zealand: Application of overseas models and multipliers. Report—New Zealand Government, Christ Church, New Zealand. 2011.

10. Tam CC, Rodrigues LC, Viviani L, Dodds JP, Evans MR, Hunter PR, et al. Longitudinal study of infectious intestinal disease in the UK (IID2 study): incidence in the community and presenting to general practice. Gut. 2012;61:69-77.

11. Scallan E, Griffin PM, Angulo FJ, Tauxe RV, Hoekstra RM. Foodborne illness acquired in the United States--unspecified agents. Emerg Infect Dis. 2011;17:16-22.

12. McGrogan A, Madle GC, Seaman HE, de Vries CS. The epidemiology of Guillain-Barre syndrome worldwide. A systematic literature review. Neuroepidemiology. 2009;32:150-163.

13. Poropatich KO, Walker CL, Black RE. Quantifying the association between Campylobacter infection and Guillain-Barre syndrome: a systematic review. J Health Popul Nutr. 2010;28:545-552.

14. Lamberti LM, Fischer Walker CL, Black RE. Systematic review of diarrhea duration and severity in children and adults in low- and middle-income countries. BMC Public Health. 2012;12:276.

15. European Center for Disease Control. Disease specific information: Campylobacteriosis. . 2013.

16. Salomon JA, Vos T, Hogan DR, Gagnon M, Naghavi M, Mokdad A, et al. Common values in assessing health outcomes from disease and injury: disability weights measurement study for the Global Burden of Disease Study 2010. Lancet. 2012;380:2129-2143.

17. Lozano R, Naghavi M, Foreman K, Lim S, Shibuya K, Aboyans V, et al. Global and regional mortality from 235 causes of death for 20 age groups in 1990 and 2010: a systematic analysis for the Global Burden of Disease Study 2010. Lancet. 2012;380:2095-2128.

18. Foodborne Diseases Active Surveillance Network (FoodNet) [Internet]. Available from: <http://www.cdc.gov/foodnet/data/trends/tables>.

1. **Norovirus infection**

| INCIDENCE: | The incidence of diarrheal norovirus and vomiting only norovirus were estimated separately. The incidence of diarrheal norovirus was estimated separately for middle/high mortality countries, and low mortality countries. For the 133 middle/high mortality countries, we used a modification of the Child Health Epidemiology Reference Group (CHERG) approach [[1](#_ENREF_15_1)]. To derive "envelopes" of diarrhea cases, for children <5 years of age we used estimates of diarrhea incidence from a CHERG systematic review [[2](#_ENREF_15_2)] and for persons >5 years of age we used a FERG-commissioned systematic review [[3](#_ENREF_15_3)]. We then estimated the etiological proportions of diarrheal illnesses due to norovirus and the 10 other diarrheal pathogens* in children <5 years of age using a CHERG and FERG systematic review of etiology studies among outpatients and persons in the community, and the etiological proportions of diarrheal illnesses due to norovirus and the 10 other diarrheal pathogens in persons >5 years of age using an updated FERG systematic review of etiology studies among inpatients, outpatients and persons in the community [[4](#_ENREF_15_4)]; these systematic reviews were supplemented by a FERG-commissioned norovirus systematic review [[5](#_ENREF_15_5)]. The norovirus etiological proportions were extracted from studies, and regional median norovirus etiological proportions calculated. We modified the CHERG approach by dropping regional median norovirus etiological proportion outliers that were >5 times greater than the global median norovirus etiological proportion, and replacing missing regional norovirus etiological proportions with the global median. Furthermore, for children <5 years of age, we proportionally decreased the etiological proportions for all 11 diarrheal pathogens in each region so that the sum of the etiological proportions for all diarrheal pathogens in a region equaled 1. The resultant regional norovirus etiological proportions were multiplied by the regional estimates of diarrhea incidence, and the resultant regional norovirus incidence was applied to all countries in that WHO region.  In the 61 low mortality countries (EUR and other WHO subregion "A" countries), we used a literature review that identified national incidence estimates for norovirus from seven countries: Australia [[6](#_ENREF_15_6)], Canada [[7](#_ENREF_15_7)], France [[8](#_ENREF_15_8)], Netherlands [[9](#_ENREF_15_9)], New Zealand [[10](#_ENREF_15_10)], United Kingdom [[11](#_ENREF_15_11)], and the United States [[12](#_ENREF_15_12)]. These national estimates were based on systematic reviews, national surveillance data, and expert judgment. In these seven countries, we used the estimated national norovirus incidence (and range) for that country. For low mortality countries without a national estimate, we used the median norovirus incidence from the seven national studies. The median incidence was from the United States; 6,978.5 per 100,000 population with range 4,295.0 – 10,282.3. To estimate the incidence of vomiting only norovirus, based on a FERG-commissioned systematic review [[13](#_ENREF_15_13)], we multiplied the incidence of diarrheal norovirus by 19% (minimum 14% - maximum 23%). |
| --- | --- |
| CLINICAL OUTCOMES: | Clinical outcomes were acute norovirus diarrhea (severe), acute norovirus diarrhea (moderate), acute norovirus diarrhea (mild), acute norovirus vomiting-only, and norovirus death. We assumed that 0.5% of norovirus diarrhea cases resulted in severe diarrhea, 8.5% of norovirus diarrhea cases resulted in moderate diarrhea, and 91% of norovirus diarrhea cases resulted in mild diarrhea. |
| DURATION: | The duration of norovirus diarrhea was estimated to be 2 days (minimum 1 day - maximum 4 days). We assumed norovirus vomiting-only cases had the same duration as norovirus diarrhea cases. |
| DISABILITY WEIGHT: | Acute norovirus diarrhea (severe): GBD 2010 disability weight of 0.281 (95% CI 0.184-0.399) for diarrhea, severe. Acute norovirus diarrhea (moderate): GBD 2010 disability weight of 0.202 (95% CI 0.133-0.299) for diarrhea, moderate. Acute norovirus diarrhea (mild) and acute norovirus vomiting-only: GBD 2010 disability weight of 0.061 (95% CI 0.036-0.093) for diarrhea, mild [[14](#_ENREF_15_14)]. |
| MORTALITY: | The mortality of norovirus was estimated separately for middle/high mortality countries, and low mortality countries. For the 133 middle/high mortality countries, we used a modification of the CHERG approach [[1](#_ENREF_15_1)]. We received envelopes of diarrheal death from WHO; because this estimate was not available with an uncertainty interval, we used the uncertainty range from the GBD 2010 estimate of diarrheal deaths (81.7% to 114.6% around the point estimate) [[15](#_ENREF_15_15)]. We then estimated the etiological proportions of diarrheal deaths due to norovirus and the other 10 diarrheal pathogens* in children <5 years of age using a CHERG and FERG systematic review of etiology studies among inpatients, and the etiological proportions of diarrheal deaths due to norovirus and the other 10 diarrheal pathogens in persons >5 years of age using an updated FERG systematic review of etiology studies among inpatients [[4](#_ENREF_15_4)]; these systematic reviews were supplemented by a FERG-commissioned norovirus systematic review [[5](#_ENREF_15_5)]. The norovirus etiological proportions were extracted from studies, and regional median norovirus etiological proportions calculated. We modified the CHERG approach by dropping regional median norovirus etiological proportion outliers that were >5 times greater than the global median norovirus etiological proportion, and replacing missing regional norovirus etiological proportions with the global median. Furthermore, for children <5 years of age, we proportionally decreased the etiological proportions for all 11 diarrheal pathogens in each region so that the sum of the etiological proportions for all diarrheal pathogens in a region equaled 1. The resultant regional norovirus etiological proportions were multiplied by the regional estimates of diarrhea deaths, and the resultant regional norovirus mortality was applied to all countries in that WHO region. In the 61 low mortality countries (EUR and other WHO subregion "A" countries), we used a literature review that identified norovirus mortality estimates from four countries: Australia [[6](#_ENREF_15_6)], Netherlands [[9](#_ENREF_15_9)], New Zealand [[10](#_ENREF_15_10)], and the United States [[12](#_ENREF_15_12)]. These national estimates were based on systematic reviews, national surveillance data, and expert judgment. In these four countries, we used the estimated national norovirus mortality (and range) for that country. For low mortality countries without a national estimate, we used the median norovirus mortality from the four national studies. The median norovirus mortality was the mean from New Zealand and the United States; 0.18 per 100,000 with range of 0.11- 0.28. We assumed no deaths among vomiting only norovirus cases. |
| AGE DISTRIBUTION: | In middle/high mortality countries we estimated incidence and mortality of norovirus separately for children <5 years of age and persons >5 years of age. In low mortality countries the age distribution for norovirus was 40% <5 years, 10% 5-14 years, 30% 15-44 years, 10% 45-64 years, and 10% >65 years [[16](#_ENREF_15_16)]. |
| SEX DISTRIBUTION: | Norovirus sex distribution: 50% male. |
| * 11 diarrheal pathogens are: nontyphoidal *Salmonella*, *Campylobacter*, *Shigella*, norovirus, enterotoxigenic *E. coli* (ETEC), enteropathogenic *E. coli* (EPEC), *C*ryptosporidia*, Giardia*, *Entamoeba histolytica*, other diarrheal agents not known to be foodborne (rotavirus and astrovirus), and unspecified agents. | |

Reference

1. Lanata CF, Fischer-Walker CL, Olascoaga AC, Torres CX, Aryee MJ, Black RE, et al. Global causes of diarrheal disease mortality in children <5 years of age: a systematic review. PLOS One. 2013;8:e72788.

2. Walker CL, Rudan I, Liu L, Nair H, Theodoratou E, Bhutta ZA, et al. Global burden of childhood pneumonia and diarrhoea. Lancet. 2013;381:1405-1416.

3. Walker CL, Black RE. Diarrhoea morbidity and mortality in older children, adolescents, and adults. Epidemiol Infect. 2010;138:1215-1226.

4. Fischer Walker CL, Sack D, Black RE. Etiology of diarrhea in older children, adolescents and adults: a systematic review. PLOS Negl Trop Dis. 2010;4:e768.

5. Ahmed SM, Hall AJ, Robinson AE, Verhoef L, Premkumar P, Parashar UD, et al. Global prevalence of norovirus in cases of gastroenteritis: a systematic review and meta-analysis. Lancet Infect Dis. 2014;14:725-730.

6. Kirk M, Ford L, Glass K, Hall K. Foodborne illness, Australia, circa 2000 and circa 2010. Emerg Infect Dis. 2014;20:1857-1864.

7. Thomas MK, Murray R, Flockhart L, Pintar K, Pollari F, Fazil A, et al. Estimates of the burden of foodborne illness in Canada for 30 specified pathogens and unspecified agents, circa 2006. Foodborne Pathog Dis. 2013;10:639-648.

8. Vaillant V, de Valk H, Baron E, Ancelle T, Colin P, Delmas MC, et al. Foodborne infections in France. Foodborne Pathog Dis. 2005;2:221-232.

9. Havelaar AH, Haagsma JA, Mangen MJ, Kemmeren JM, Verhoef LP, Vijgen SM, et al. Disease burden of foodborne pathogens in the Netherlands, 2009. Int J Food Microbiol. 2012;156:231-238.

10. Cressey P, Lake R. Estimated incidence of foodborne illness in New Zealand: Application of overseas models and multipliers. Report—New Zealand Government, Christ Church, New Zealand. 2011.

11. Tam CC, Rodrigues LC, Viviani L, Dodds JP, Evans MR, Hunter PR, et al. Longitudinal study of infectious intestinal disease in the UK (IID2 study): incidence in the community and presenting to general practice. Gut. 2012;61:69-77.

12. Scallan E, Griffin PM, Angulo FJ, Tauxe RV, Hoekstra RM. Foodborne illness acquired in the United States--unspecified agents. Emerg Infect Dis. 2011;17:16-22.

13. Hall A. Estimating the global burden of foodborne norovirus. Foodborne Disease Epidemiology Reference Group meeting; Geneva, Switzerland: World Health Organization; 2013.

14. Salomon JA, Vos T, Hogan DR, Gagnon M, Naghavi M, Mokdad A, et al. Common values in assessing health outcomes from disease and injury: disability weights measurement study for the Global Burden of Disease Study 2010. Lancet. 2012;380:2129-2143.

15. Lozano R, Naghavi M, Foreman K, Lim S, Shibuya K, Aboyans V, et al. Global and regional mortality from 235 causes of death for 20 age groups in 1990 and 2010: a systematic analysis for the Global Burden of Disease Study 2010. Lancet. 2012;380:2095-2128.

16. Phillips G, Tam CC, Conti S, Rodrigues LC, Brown D, Iturriza-Gomara M, et al. Community incidence of norovirus-associated infectious intestinal disease in England: improved estimates using viral load for norovirus diagnosis. Am J Epidemiol. 2010;171:1014-1022.

1. **Shigellosis**

| INCIDENCE: | The incidence of shigellosis was estimated separately for middle/high mortality countries, and low mortality countries. For the 133 middle/high mortality countries, we used a modification of the Child Health Epidemiology Reference Group (CHERG) approach [[1](#_ENREF_16_1)]. To derive "envelopes" of diarrhea cases, for children <5 years of age we used estimates of diarrhea incidence from a CHERG systematic review [[2](#_ENREF_16_2)] and for persons >5 years of age we used a FERG-commissioned systematic review [[3](#_ENREF_16_3)]. We then estimated the etiological proportions of diarrheal illnesses due to *Shigella* and the 10 other diarrheal pathogens* in children <5 years of age using a CHERG and FERG systematic review of etiology studies among outpatients and persons in the community, and the etiological proportion of diarrheal illnesses due to *Shigella* and the 10 other diarrheal pathogens in persons >5 years of age using an updated FERG systematic review of etiology studies among inpatients, outpatients and persons in the community [[4](#_ENREF_16_4)]. The shigellosis etiological proportions were extracted from studies, and regional median shigellosis etiological proportions calculated. We modified the CHERG approach by dropping regional median shigellosis etiological proportion outliers that were >5 times greater than the global median shigellosis etiological proportion, and replacing missing regional shigellosis etiological proportions with the global median. Furthermore, for children <5 years of age, we proportionally decreased the etiological proportions for all 11 diarrheal pathogens in each region so that the sum of the etiological proportions for all diarrheal pathogens in a region equaled 1. The resultant regional shigellosis etiological proportions were multiplied by the regional estimates of diarrhea incidence, and the resultant regional shigellosis incidence was applied to all countries in that WHO region.  In the 61 low mortality countries (EUR and other WHO subregion "A" countries), we used a literature review that identified national incidence estimates for shigellosis from five countries: Australia [[5](#_ENREF_16_5)], Canada [[6](#_ENREF_16_6)], France [[7](#_ENREF_16_7)], Netherlands [[8](#_ENREF_16_8)], New Zealand [[9](#_ENREF_16_9)], and the United States [[10](#_ENREF_16_10)]. These national estimates were based on systematic reviews, national surveillance data, and expert judgment. In these five countries, we used the estimated national shigellosis incidence (and range) for that country. For low mortality countries without a national estimate, we used the median shigellosis incidence from the five national studies. The median incidence was from Canada (which was increased by 8% to account for travelers using proxy information from the United States) which was 23.6 per 100,000 population with range 13.2 – 38.7. |
| --- | --- |
| CLINICAL OUTCOMES: | Clinical outcomes were acute *Shigella* diarrhea (severe), acute *Shigella* diarrhea (moderate), acute *Shigella* diarrhea (mild), and *Shigella* death. We assumed that 2% of *Shigella* cases resulted in severe diarrhea, 25% of *Shigella* cases resulted in moderate diarrhea, and 73% of *Shigella* cases resulted in mild diarrhea. |
| DURATION: | In children <5 years of age, duration of severe diarrhea was 8.4 days, moderate diarrhea was 6.4 days, and mild diarrhea was 4.3 days [[11](#_ENREF_16_11)]. Based on the assumed distribution of severe, moderate and mild diarrhea cases, the duration of *Shigella* diarrhea cases in children <5 years of age was estimated to be 4.9 days (minimum 4.3 days - maximum 8.4 days). In persons >5 years of age, the duration of *Shigella* diarrhea was 2.8 days [[11](#_ENREF_16_11)]. |
| DISABILITY WEIGHT: | Acute *Shigella* diarrhea (severe): GBD 2010 disability weight of 0.281 (95% CI 0.184-0.399) for diarrhea, severe. Acute *Shigella* diarrhea (moderate): GBD 2010 disability weight of 0.202 (95% CI 0.133-0.299) for diarrhea, moderate. Acute *Shigella* diarrhea (mild): GBD 2010 disability weight of 0.061 (95% CI 0.036-0.093) for diarrhea, mild [[12](#_ENREF_16_12)]. |
| MORTALITY: | The mortality of shigellosis was estimated separately for middle/high mortality countries, and low mortality countries. For the 133 middle/high mortality countries, we used a modification of the CHERG approach [[1](#_ENREF_16_1)]. We received envelopes of diarrheal deaths from WHO; because this estimate was not available with an uncertainty interval, we used the uncertainty range from the GBD 2010 estimate of diarrheal deaths (81.7% to 114.6% around the point estimate) [[13](#_ENREF_16_13)]. We then estimated the etiological proportions of diarrheal deaths due to *Shigella* and 10 other diarrheal pathogens* in children <5 years of age using a CHERG and FERG systematic review of etiology studies among inpatients, and the etiological proportions of diarrheal deaths due to *Shigella* and the 10 other diarrheal pathogens in persons >5 years of age using an updated FERG systematic review of etiology studies among inpatients [[4](#_ENREF_16_4)]. The shigellosis etiological proportions were extracted from studies, and regional median shigellosis etiological proportions calculated. We modified the CHERG approach by dropping regional median shigellosis etiological proportion outliers that were >5 times greater than the global median shigellosis etiological proportion, and replacing missing regional shigellosis etiological proportions with the global median. Furthermore, for children <5 years of age, we proportionally decreased the etiological proportions for all 11 diarrheal pathogens in each region so that the sum of the etiological proportions for all diarrheal pathogens in a region equaled 1. The resultant regional shigellosis etiological proportions were multiplied by the regional estimates of diarrhea deaths, and the resultant regional shigellosis mortality was applied to all countries in that WHO region. In the 61 low mortality countries (EUR and other WHO subregion "A" countries), we used a literature review that identified shigellosis mortality estimates from the United States [[10](#_ENREF_16_10)]. This national estimate was based on national surveillance data, and expert judgment. We used the shigellosis mortality from the United States for all low mortality countries; 0.013 per 100,000 population with range 0.002 – 0.085. |
| AGE DISTRIBUTION: | In middle/high mortality countries we estimated incidence and mortality of *Shigella* separately for children <5 years of age and persons >5 years of age. In low mortality countries the age distribution for *Shigella* cases was 24% <5 years, 23% 5-14 years, 10% 15-24 years, 39% 25-64 years, and 4% >65 years [[14](#_ENREF_16_14)]. |
| SEX DISTRIBUTION: | *Shigella* sex distribution: 50% male. |
| * 11 diarrheal pathogens are: nontyphoidal *Salmonella*, *Campylobacter*, *Shigella*, norovirus, enterotoxigenic *E. coli* (ETEC), enteropathogenic *E. coli* (EPEC), *C*ryptosporidia*, Giardia*, *Entamoeba histolytica*, other diarrheal agents not known to be foodborne (rotavirus and astrovirus), and unspecified agents. | |

Reference

1. Lanata CF, Fischer-Walker CL, Olascoaga AC, Torres CX, Aryee MJ, Black RE, et al. Global causes of diarrheal disease mortality in children <5 years of age: a systematic review. PLOS One. 2013;8:e72788.

2. Walker CL, Rudan I, Liu L, Nair H, Theodoratou E, Bhutta ZA, et al. Global burden of childhood pneumonia and diarrhoea. Lancet. 2013;381:1405-1416.

3. Walker CL, Black RE. Diarrhoea morbidity and mortality in older children, adolescents, and adults. Epidemiol Infect. 2010;138:1215-1226.

4. Fischer Walker CL, Sack D, Black RE. Etiology of diarrhea in older children, adolescents and adults: a systematic review. PLOS Negl Trop Dis. 2010;4:e768.

5. Kirk M, Ford L, Glass K, Hall K. Foodborne illness, Australia, circa 2000 and circa 2010. Emerg Infect Dis. 2014;20:1857-1864.

6. Thomas MK, Murray R, Flockhart L, Pintar K, Pollari F, Fazil A, et al. Estimates of the burden of foodborne illness in Canada for 30 specified pathogens and unspecified agents, circa 2006. Foodborne Pathog Dis. 2013;10:639-648.

7. Vaillant V, de Valk H, Baron E, Ancelle T, Colin P, Delmas MC, et al. Foodborne infections in France. Foodborne Pathog Dis. 2005;2:221-232.

8. Havelaar AH, Haagsma JA, Mangen MJ, Kemmeren JM, Verhoef LP, Vijgen SM, et al. Disease burden of foodborne pathogens in the Netherlands, 2009. Int J Food Microbiol. 2012;156:231-238.

9. Cressey P, Lake R. Estimated incidence of foodborne illness in New Zealand: Application of overseas models and multipliers. Report—New Zealand Government, Christ Church, New Zealand. 2011.

10. Scallan E, Griffin PM, Angulo FJ, Tauxe RV, Hoekstra RM. Foodborne illness acquired in the United States--unspecified agents. Emerg Infect Dis. 2011;17:16-22.

11. Lamberti LM, Fischer Walker CL, Black RE. Systematic review of diarrhea duration and severity in children and adults in low- and middle-income countries. BMC Public Health. 2012;12:276.

12. Salomon JA, Vos T, Hogan DR, Gagnon M, Naghavi M, Mokdad A, et al. Common values in assessing health outcomes from disease and injury: disability weights measurement study for the Global Burden of Disease Study 2010. Lancet. 2012;380:2129-2143.

13. Lozano R, Naghavi M, Foreman K, Lim S, Shibuya K, Aboyans V, et al. Global and regional mortality from 235 causes of death for 20 age groups in 1990 and 2010: a systematic analysis for the Global Burden of Disease Study 2010. Lancet. 2012;380:2095-2128.

14. Foodborne Diseases Active Surveillance Network (FoodNet) [Internet]. Available from: <http://www.cdc.gov/foodnet/data/trends/tables>.

1. **Enterotoxigenic *Escherichia coli* (ETEC) infection**

| INCIDENCE: | The incidence of diarrhea due to ETEC was estimated separately for middle/high mortality countries, and low mortality countries. For the 133 middle/high mortality countries, we used a modification of the Child Health Epidemiology Reference Group (CHERG) approach [[1](#_ENREF_17_1)]. To derive "envelopes" of diarrhea cases, for children <5 years of age we used estimates of diarrhea incidence from a CHERG systematic review [[2](#_ENREF_17_2)] and for persons >5 years of age we used a FERG-commissioned systematic review [[3](#_ENREF_17_3)]. We then estimated the etiological proportions of diarrheal illnesses due to ETEC and 10 other diarrheal pathogens* in children <5 years of age using a CHERG and FERG systematic review of etiology studies among outpatients and persons in the community, and the etiological proportion of diarrheal illnesses due to ETEC and 10 other diarrheal pathogens in persons >5 years of age using an updated FERG systematic review of etiology studies among inpatients, outpatients and persons in the community [[4](#_ENREF_17_4)]. The ETEC etiological proportions were extracted from studies, and regional median ETEC etiological proportions calculated. We modified the CHERG approach by dropping regional median ETEC etiological proportion outliers that were >5 times greater than the global median ETEC etiological proportion, and replacing missing regional ETEC etiological proportions with the global median. Furthermore, for children <5 years of age, we proportionally decreased the etiological proportions for all 11 diarrheal pathogens in each region so that the sum of the etiological proportions for all diarrheal pathogens in a region equaled 1. The resultant regional ETEC etiological proportions were multiplied by the regional estimates of diarrhea incidence, and the resultant regional ETEC incidence was applied to all countries in that WHO region. In the 61 low mortality countries (EUR and other WHO subregion "A" countries), a literature review identified a national incidence estimates for ETEC in the United States which was based on national surveillance data, and expert judgment [[5](#_ENREF_17_5)]. We used the ETEC incidence from the United States for all low mortality countries; 13.3 per 100,000 population with range 3.9 – 34.2. |
| --- | --- |
| CLINICAL OUTCOMES: | Clinical outcomes were acute ETEC diarrhea (severe), acute ETEC diarrhea (moderate), acute ETEC diarrhea (mild), and death. We assumed that 0.5% of ETEC cases resulted in severe diarrhea, 8.5% of ETEC cases resulted in moderate diarrhea, and 91% of ETEC cases resulted in mild diarrhea. |
| DURATION: | In children <5 years of age, duration of severe diarrhea was 8.4 days, moderate diarrhea was 6.4 days, and mild diarrhea was 4.3 days [[6](#_ENREF_17_6)]. Based on the assumed distribution of severe, moderate and mild diarrhea cases, the duration of ETEC diarrhea cases in children <5 years of age was estimated to be 4.9 days (minimum 4.3 days - maximum 8.4 days). In persons >5 years of age, the duration of ETEC diarrhea was 2.8 days [[6](#_ENREF_17_6)]. |
| DISABILITY WEIGHT: | Acute ETEC diarrhea (severe): GBD 2010 disability weight of 0.281 (95% CI 0.184-0.399) for diarrhea, severe. Acute ETEC diarrhea (moderate): GBD 2010 disability weight of 0.202 (95% CI 0.133-0.299) for diarrhea, moderate. Acute ETEC diarrhea (mild): GBD 2010 disability weight of 0.061 (95% CI 0.036-0.093) for diarrhea, mild [[7](#_ENREF_17_7)]. |
| MORTALITY: | The mortality of ETEC was estimated separately for middle/high mortality countries, and low mortality countries. For the 133 middle/high mortality countries, we used a modification of the CHERG approach [[1](#_ENREF_17_1)]. We received envelopes of diarrheal deaths from WHO; because this estimate was not available with an uncertainty interval, we used the uncertainty range from the GBD 2010 estimate of diarrheal deaths (81.7% to 114.6% around the point estimate) [[8](#_ENREF_17_8)]. We then estimated the etiological proportions of diarrheal deaths due to ETEC and 10 other diarrheal pathogens* in children <5 years of age using a CHERG and FERG systematic review of etiology studies among inpatients, and the etiological proportions of diarrheal deaths due to ETEC and 10 other diarrheal pathogens in persons >5 years of age using an updated FERG systematic tic review of etiology studies among inpatients [[4](#_ENREF_17_4)]. The ETEC etiological proportions were extracted from studies, and regional median ETEC etiological proportions calculated. We modified the CHERG approach by dropping regional median ETEC etiological proportion outliers that were >5 times greater than the global median ETEC etiological proportion, and replacing missing regional ETEC etiological proportions with the global median. Furthermore, for children <5 years of age, we proportionally decreased the etiological proportions for all 11 diarrheal pathogens in each region so that the sum of the etiological proportions for all diarrheal pathogens in a region equaled 1. The resultant ETEC etiological proportions were multiplied by the regional estimates of diarrhea deaths, and the resultant regional ETEC mortality was applied to all countries in that WHO region. We estimated no ETEC deaths in the 61 low mortality countries (EUR and other WHO subregion "A" countries). |
| AGE DISTRIBUTION: | In middle/high mortality countries we estimated incidence of diarrhea separately for children <5 years of age and persons >5 years of age. In low mortality countries, no information was available on the age distribution of ETEC cases; therefore, used the age distribution for *Campylobacter* diarrhea cases as a proxy, which was 11% <5 years, 8% 5-14 years, 10% 15-24 years, 57% 25-64 years, and 14% >65 years. |
| SEX DISTRIBUTION: | ETEC sex distribution: 50% male. |
| * 11 diarrheal pathogens are: nontyphoidal *Salmonella*, *Campylobacter*, *Shigella*, norovirus, enterotoxigenic *E. coli* (ETEC), enteropathogenic *E. coli* (EPEC), *C*ryptosporidia*, Giardia*, *Entamoeba histolytica*, other diarrheal agents not known to be foodborne (rotavirus and astrovirus), and unspecified agents. | |

Reference

1. Lanata CF, Fischer-Walker CL, Olascoaga AC, Torres CX, Aryee MJ, Black RE, et al. Global causes of diarrheal disease mortality in children <5 years of age: a systematic review. PLOS One. 2013;8:e72788.

2. Walker CL, Rudan I, Liu L, Nair H, Theodoratou E, Bhutta ZA, et al. Global burden of childhood pneumonia and diarrhoea. Lancet. 2013;381:1405-1416.

3. Walker CL, Black RE. Diarrhoea morbidity and mortality in older children, adolescents, and adults. Epidemiol Infect. 2010;138:1215-1226.

4. Fischer Walker CL, Sack D, Black RE. Etiology of diarrhea in older children, adolescents and adults: a systematic review. PLOS Negl Trop Dis. 2010;4:e768.

5. Scallan E, Griffin PM, Angulo FJ, Tauxe RV, Hoekstra RM. Foodborne illness acquired in the United States--unspecified agents. Emerg Infect Dis. 2011;17:16-22.

6. Lamberti LM, Fischer Walker CL, Black RE. Systematic review of diarrhea duration and severity in children and adults in low- and middle-income countries. BMC Public Health. 2012;12:276.

7. Salomon JA, Vos T, Hogan DR, Gagnon M, Naghavi M, Mokdad A, et al. Common values in assessing health outcomes from disease and injury: disability weights measurement study for the Global Burden of Disease Study 2010. Lancet. 2012;380:2129-2143.

8. Lozano R, Naghavi M, Foreman K, Lim S, Shibuya K, Aboyans V, et al. Global and regional mortality from 235 causes of death for 20 age groups in 1990 and 2010: a systematic analysis for the Global Burden of Disease Study 2010. Lancet. 2012;380:2095-2128.

1. **Enteropathogenic *Escherichia coli* (EPEC) infection**

| INCIDENCE: | The incidence of diarrhea due to EPEC was estimated separately for middle/high mortality countries, and low mortality countries. For the 133 middle/high mortality countries, we used a modification of the Child Health Epidemiology Reference Group (CHERG) approach [[1](#_ENREF_18_1)]. To derive "envelopes" of diarrhea cases, for children <5 years of age we used estimates of diarrhea incidence from a CHERG systematic review [[2](#_ENREF_18_2)] and for persons >5 years of age we used a FERG-commissioned systematic review [[3](#_ENREF_18_3)]. We then estimated the etiological proportions of diarrheal illnesses due to EPEC and the 10 other diarrheal pathogens* in children <5 years of age using a CHERG and FERG systematic review of etiology studies among outpatients and persons in the community, and the etiological proportion of diarrheal illnesses due to EPEC and the 10 other diarrheal pathogens in persons >5 years of age using an updated FERG systematic review of etiology studies among inpatients, outpatients and persons in the community [[4](#_ENREF_18_4)]. The EPEC etiological proportions were extracted from studies, and regional median EPEC etiological proportions calculated. We modified the CHERG approach by dropping regional median EPEC etiological proportion outliers that were >5 times greater than the global median EPEC etiological proportion, and replacing missing regional EPEC etiological proportions with the global median. Furthermore, for children <5 years of age, we proportionally decreased the etiological proportions for all 11 diarrheal pathogens in each region so that the sum of the etiological proportions for all diarrheal pathogens in a region equaled 1. The resultant regional EPEC etiological proportions were multiplied by the regional estimates of diarrhea incidence, and the resultant regional EPEC incidence was applied to all countries in that WHO region. In the 61 low mortality countries (EUR and other WHO subregion "A" countries), we adopted the assumption used in the national study in the United States that EPEC was as common at enterotoxigenic E. coli [[5](#_ENREF_18_5)]. The national estimate for ETEC in the United States was based on national surveillance data, and expert judgment. For low mortality countries, we used the EPEC incidence from the United States which was 13.33 per 100,000 population with range 4.00 – 34.24. |
| --- | --- |
| CLINICAL OUTCOMES: | Clinical outcomes were acute EPEC diarrhea (severe), acute EPEC diarrhea (moderate), acute EPEC diarrhea (mild), and EPEC death. We assumed that 0.5% of EPEC cases resulted in severe diarrhea, 8.5% of EPEC cases resulted in moderate diarrhea, and 91% of EPEC cases resulted in mild diarrhea. |
| DURATION: | In children <5 years of age, duration of severe diarrhea was 8.4 days, moderate diarrhea was 6.4 days, and mild diarrhea was 4.3 days [[5](#_ENREF_18_5)]. Based on the assumed distribution of severe, moderate and mild diarrhea cases, the duration of EPEC diarrhea cases in children <5 years of age was estimated to be 4.9 days (minimum 4.3 days - maximum 8.4 days). In persons >5 years of age, the duration of diarrhea was 2.8 days [[6](#_ENREF_18_6)]. |
| DISABILITY WEIGHT: | Acute EPEC diarrhea (severe): GBD 2010 disability weight of 0.281 (95% CI 0.184-0.399) for diarrhea, severe. Acute EPEC diarrhea (moderate): GBD 2010 disability weight of 0.202 (95% CI 0.133-0.299) for diarrhea, moderate. Acute EPEC diarrhea (mild): GBD 2010 disability weight of 0.061 (95% CI 0.036-0.093) for diarrhea, mild [[7](#_ENREF_18_7)]. |
| MORTALITY: | The mortality of EPEC was estimated separately for middle/high mortality countries, and low mortality countries. For the 133 middle/high mortality countries, we used a modification of the CHERG approach [[1](#_ENREF_18_1)]. We received envelopes of diarrheal deaths from WHO; because this estimate was not available with an uncertainty interval, we used the uncertainty range from the GBD 2010 estimate of diarrheal deaths (81.7% to 114.6% around the point estimate) [[8](#_ENREF_18_8)]. We then estimated the etiological proportions of diarrheal deaths due to EPEC and the 10 other diarrheal pathogens* in children <5 years of age using a CHERG and FERG systematic review of etiology studies among inpatients, and the etiological proportions of diarrheal deaths due to EPEC and the 10 other diarrheal pathogens in persons >5 years of age using an updated FERG systematic tic review of etiology studies among inpatients [[4](#_ENREF_18_4)]. The EPEC etiological proportions were extracted from studies, and regional median EPEC etiological proportions calculated. We modified the CHERG approach by dropping regional median EPEC etiological proportion outliers that were >5 times greater than the global median EPEC etiological proportion, and replacing missing regional EPEC etiological proportions with the global median. Furthermore, for children <5 years of age, we proportionally decreased the etiological proportions for all 11 diarrheal pathogens in each region so that the sum of the etiological proportions for all diarrheal pathogens in a region equaled 1. The resultant EPEC etiological proportions were multiplied by the regional estimates of diarrhea deaths, and the resultant regional EPEC mortality was applied to all countries in that WHO region. We estimated no EPEC deaths in the 61 low mortality countries (EUR and other WHO subregion "A" countries). |
| AGE DISTRIBUTION: | In middle/high mortality countries we estimated incidence and mortality of EPEC separately for children <5 years of age and persons >5 years of age. In low mortality countries, no information was available on the age distribution of EPEC cases; therefore, used the age distribution for *Campylobacter* diarrhea cases as a proxy, which was 11% <5 years, 8% 5-14 years, 10% 15-24 years, 57% 25-64 years, and 14% >65 years. |
| SEX DISTRIBUTION: | EPEC sex distribution: 50% male. |
| * 11 diarrheal pathogens are: nontyphoidal *Salmonella*, *Campylobacter*, *Shigella*, norovirus, enterotoxigenic *E. coli* (ETEC), enteropathogenic *E. coli* (EPEC), *C*ryptosporidia*, Giardia*, *Entamoeba histolytica*, other diarrheal agents not known to be foodborne (rotavirus and astrovirus), and unspecified agents. | |

Reference

1. Lanata CF, Fischer-Walker CL, Olascoaga AC, Torres CX, Aryee MJ, Black RE, et al. Global causes of diarrheal disease mortality in children <5 years of age: a systematic review. PLOS One. 2013;8:e72788.

2. Walker CL, Rudan I, Liu L, Nair H, Theodoratou E, Bhutta ZA, et al. Global burden of childhood pneumonia and diarrhoea. Lancet. 2013;381:1405-1416.

3. Walker CL, Black RE. Diarrhoea morbidity and mortality in older children, adolescents, and adults. Epidemiol Infect. 2010;138:1215-1226.

4. Fischer Walker CL, Sack D, Black RE. Etiology of diarrhea in older children, adolescents and adults: a systematic review. PLOS Negl Trop Dis. 2010;4:e768.

5. Scallan E, Griffin PM, Angulo FJ, Tauxe RV, Hoekstra RM. Foodborne illness acquired in the United States--unspecified agents. Emerg Infect Dis. 2011;17:16-22.

6. Lamberti LM, Fischer Walker CL, Black RE. Systematic review of diarrhea duration and severity in children and adults in low- and middle-income countries. BMC Public Health. 2012;12:276.

7. Salomon JA, Vos T, Hogan DR, Gagnon M, Naghavi M, Mokdad A, et al. Common values in assessing health outcomes from disease and injury: disability weights measurement study for the Global Burden of Disease Study 2010. Lancet. 2012;380:2129-2143.

8. Lozano R, Naghavi M, Foreman K, Lim S, Shibuya K, Aboyans V, et al. Global and regional mortality from 235 causes of death for 20 age groups in 1990 and 2010: a systematic analysis for the Global Burden of Disease Study 2010. Lancet. 2012;380:2095-2128.

1. **Cholera**

| INCIDENCE: | Estimates of the incidence of cholera were adapted from a published systematic review of the global burden of cholera [[1](#_ENREF_19_1)] updated with 2010 population estimates. This review classified 51 countries as cholera endemic countries based on results of the systematic review and national cholera reports in the WHO *Weekly Epidemiological Record*. The review then used WHO 2008 country-specific estimates of the proportion of each country's population that lacked improved sanitation [[2](#_ENREF_19_2)] to estimate the proportion of the population in the cholera endemic countries that were at risk for cholera. Then a cholera incidence was assigned to the population at risk for cholera in the cholera endemic countries based on population-based studies in India [[3](#_ENREF_19_3)], Indonesia [[4](#_ENREF_19_4)] and Mozambique [[5](#_ENREF_19_5)]. The review also identified an additional 18 countries that reported cholera to WHO during 2000 to 2008 but were judged to be not be endemic for cholera; a country-specific cholera incidence in each of these "non-endemic" countries was estimated using the annual average number of cholera cases reported to WHO cases in each country times a multiplier of 10 to account for under-reporting. For all other countries, we used a literature review that identified national cholera incidence estimates from three countries countries: France [[6](#_ENREF_19_6)], New Zealand [[7](#_ENREF_19_7)], and the United States [[8](#_ENREF_19_8)]. The cholera incidence in the United States was the median estimate from these three countries and was used (0.093 per 100,000 population) as the cholera incidence for all countries (other than the cholera endemic and non-endemic countries) which did not have national incidence estimates. We used the global burden of cholera [[1](#_ENREF_19_1)] range of estimates around the mean estimate of global cholera cases (2.8 million with 1.4 to 4.3 million) to derive a range of estimates for cholera incidence. |
| --- | --- |
| CLINICAL OUTCOMES: | Clinical outcomes were cholera (severe), cholera (moderate), cholera (mild), and cholera death. We assumed that 35% of cholera cases resulted in severe cholera, 40% of cholera cases resulted in moderate cholera, and 25% of cholera cases resulted in mild cholera [[9](#_ENREF_19_9), [10](#_ENREF_19_10)]. |
| DURATION: | We assumed the duration of cholera was 7 days (minimum duration 3 day - maximum duration 10 days). |
| DISABILITY WEIGHT: | Cholera (severe): GBD 2010 disability weight of 0.281 (95% CI 0.184-0.399) for diarrhea, severe. Cholera (moderate): GBD 2010 disability weight of 0.202 (95% CI 0.133-0.299) for diarrhea, moderate. Cholera (mild): GBD 2010 disability weight of 0.061 (95% CI 0.036-0.093) for diarrhea, mild [[11](#_ENREF_19_11)]. |
| MORTALITY: | For 51 cholera endemic and 18 cholera non-endemic countries, we used the case fatality ratios (CFRs) estimated in the systematic review of the global burden of cholera [[1](#_ENREF_19_1)]. This review calculated a variance-weighted average cholera CFRs by WHO region; the CFR was 1% in WPR subregion B, 1% in SEAR B (except 1.5% in Bangladesh), 1.3% in EMR B, 3% in SEAR D, 3.2% in EMR D, and 3.8% in AFR. For all other countries, the literature review of national incidence estimates for cholera identified no reported deaths; therefore we assumed no cholera deaths occurred in countries (other than the cholera endemic and non-endemic countries). We used the global burden of cholera [[1](#_ENREF_19_1)] range of estimates around the mean estimate of global cholera deaths (91,000 with 28,000 to 142,000) to derive a range of estimates for cholera deaths. |
| AGE DISTRIBUTION: | Cholera age distribution: 15% <5 years, 25% 5-14 years, 42% 15-34 years, 15% 35-64, 3% >60 years [[12](#_ENREF_19_12), [13](#_ENREF_19_13)]. |
| SEX DISTRIBUTION: | Cholera sex distribution: 50% male. |

Reference

1. Ali M, Lopez AL, You YA, Kim YE, Sah B, Maskery B, et al. The global burden of cholera. Bulletin World Health Organization. 2012;90:209-218A.

2. Organization WH. Population with sustanable access to improved sanitation.

3. Sur D, Deen JL, Manna B, Niyogi SK, Deb AK, Kanungo S, et al. The burden of cholera in the slums of Kolkata, India: data from a prospective, community based study. Archives of Diseases of Childhood. 2005;90:1175-1181.

4. Agtini MD, Soeharno R, Lesmana M, Punjabi NH, Simanjuntak C, Wangsasaputra F, et al. The burden of diarrhoea, shigellosis, and cholera in North Jakarta, Indonesia: findings from 24 months surveillance. BMC Infect Dis. 2005;5:89.

5. Lucas ME, Deen JL, von Seidlein L, Wang XY, Ampuero J, Puri M, et al. Effectiveness of mass oral cholera vaccination in Beira, Mozambique. New Engl J Med. 2005;352:757-767.

6. Vaillant V, de Valk H, Baron E, Ancelle T, Colin P, Delmas MC, et al. Foodborne infections in France. Foodborne Pathog Dis. 2005;2:221-232.

7. Cressey P, Lake R. Estimated incidence of foodborne illness in New Zealand: Application of overseas models and multipliers. Report—New Zealand Government, Christ Church, New Zealand. 2011.

8. Scallan E, Griffin PM, Angulo FJ, Tauxe RV, Hoekstra RM. Foodborne illness acquired in the United States--unspecified agents. Emerg Infect Dis. 2011;17:16-22.

9. Siddique AK, Nair GB, Alam M, Sack DA, Huq A, Nizam A, et al. El Tor cholera with severe disease: a new threat to Asia and beyond. Epidemiol Infect. 2010;138:347-352.

10. Valcin CL, Severe K, Riche CT, Anglade BS, Moise CG, Woodworth M, et al. Predictors of disease severity in patients admitted to a cholera treatment center in urban Haiti. Am J Trop Med Hyg. 2013;89:625-632.

11. Salomon JA, Vos T, Hogan DR, Gagnon M, Naghavi M, Mokdad A, et al. Common values in assessing health outcomes from disease and injury: disability weights measurement study for the Global Burden of Disease Study 2010. Lancet. 2012;380:2129-2143.

12. Gujral L, Sema C, Rebaudet S, Taibo CL, Manjate AA, Piarroux R, et al. Cholera epidemiology in Mozambique using national surveillance data. J Infect Dis. 2013;208 Suppl 1:S107-114.

13. Qadri F, Khan AI, Faruque AS, Begum YA, Chowdhury F, Nair GB, et al. Enterotoxigenic Escherichia coli and Vibrio cholerae diarrhea, Bangladesh, 2004. Emerg Infect Dis. 2005;11:1104-1107.

1. **Cryptosporidiosis**

| INCIDENCE: | The incidence of cryptosporidiosis was estimated separately for middle/high mortality countries, and low mortality countries. For the 133 middle/high mortality countries, we used a modification of the Child Health Epidemiology Reference Group (CHERG) approach [[1](#_ENREF_20_1)]. To derive "envelopes" of diarrhea cases, for children <5 years of age we used estimates of diarrhea incidence from a CHERG systematic review [[2](#_ENREF_20_2)] and for persons >5 years of age we used a FERG-commissioned systematic review [[3](#_ENREF_20_3)]. We then estimated the etiological proportions of diarrheal illnesses due to *Cryptosporidia* and 10 other diarrheal pathogens* in children <5 years of age using a CHERG and FERG systematic review of etiology studies among outpatients and persons in the community, and the etiological proportion of diarrheal illnesses due to *Cryptosporidia* and 10 other diarrheal pathogens in persons >5 years of age using an updated FERG systematic review of etiology studies among inpatients, outpatients and persons in the community [[4](#_ENREF_20_4)]. The cryptosporidiosis etiological proportions were extracted from studies, and regional median cryptosporidiosis etiological proportions calculated. We modified the CHERG approach by dropping regional median cryptosporidiosis etiological proportion outliers that were >5 times greater than the global median cryptosporidiosis etiological proportion, and replacing missing regional cryptosporidiosis etiological proportions with the global median. Furthermore, for children <5 years of age, we proportionally decreased the etiological proportions for all 11 diarrheal pathogens in each region so that the sum of the etiological proportions for all diarrheal pathogens in a region equaled 1. The resultant regional cryptosporidiosis etiological proportions were multiplied by the regional estimates of diarrhea incidence, and the resultant regional cryptosporidiosis incidence was applied to all countries in that WHO region. In the 61 low mortality countries (EUR and other WHO subregion "A" countries), we used a literature review that identified national incidence estimates for cryptosporidiosis from six countries: Australia [[5](#_ENREF_20_5)], Canada [[6](#_ENREF_20_6)], Netherlands [[7](#_ENREF_20_7)], New Zealand [[8](#_ENREF_20_8)], United Kingdom [[9](#_ENREF_20_9)], and the United States [[10](#_ENREF_20_10)]. These national estimates were based on systematic reviews, national surveillance data, and expert judgment. In these six countries, we used the estimated national cryptosporidiosis incidence (and range) for that country. For low mortality countries without a national estimate, we used the median cryptosporidiosis incidence from the six national studies. The median incidence was the mean from Australia (which was increased by 19% to account for travelers using proxy information from New Zealand) and the Netherlands, which was 128.4 per 100,000 population with range 50.33 – 601.63. |
| --- | --- |
| CLINICAL OUTCOMES: | Clinical outcomes were acute cryptosporidiosis diarrhea (severe), acute cryptosporidiosis diarrhea (moderate), acute cryptosporidiosis diarrhea (mild), and death. We assumed that 0.5% of cryptosporidiosis cases resulted in severe diarrhea, 8.5% of cryptosporidiosis cases resulted in moderate diarrhea, and 91% of cryptosporidiosis cases resulted in mild diarrhea. |
| DURATION: | In children <5 years of age, duration of severe diarrhea was 8.4 days, moderate diarrhea was 6.4 days, and mild diarrhea was 4.3 days [[11](#_ENREF_20_11)]. Based on the assumed distribution of severe, moderate and mild diarrhea cases, the duration of cryptosporidiosis diarrhea cases in children <5 years of age was estimated to be 4.9 days (minimum 4.3 days - maximum 8.4 days). In persons >5 years of age, the duration of diarrhea was 2.8 days [[11](#_ENREF_20_11)]. |
| DISABILITY WEIGHT: | Acute cryptosporidiosis diarrhea (severe): GBD 2010 disability weight of 0.281 (95% CI 0.184-0.399) for diarrhea, severe. Acute cryptosporidiosis diarrhea (moderate): GBD 2010 disability weight of 0.202 (95% CI 0.133-0.299) for diarrhea, moderate. Acute cryptosporidiosis diarrhea (mild): GBD 2010 disability weight of 0.061 (95% CI 0.036-0.093) for diarrhea, mild [[12](#_ENREF_20_12)]. |
| MORTALITY: | The mortality of cryptosporidiosis was estimated separately for middle/high mortality countries, and low mortality countries. For the 133 middle/high mortality countries, we used a modification of the CHERG approach [[1](#_ENREF_20_1)]. We received envelopes of diarrheal deaths from WHO; because this estimate was not available with an uncertainty interval, we used the uncertainty range from the GBD 2010 estimate of diarrheal deaths (81.7% to 114.6% around the point estimate) [[13](#_ENREF_20_13)]. We then estimated the etiological proportions of diarrheal deaths due to *Cryptosporidium* and 10 other diarrheal pathogens* in children <5 years of age using a CHERG and FERG systematic review of etiology studies among inpatients, and the etiological proportions of diarrheal deaths due to *Cryptosporidium* and 10 other diarrheal pathogens in persons >5 years of age using an updated FERG systematic tic review of etiology studies among inpatients [[4](#_ENREF_20_4)]. The cryptosporidiosis etiological proportions were extracted from studies, and regional median cryptosporidiosis etiological proportions calculated. We modified the CHERG approach by dropping regional median cryptosporidiosis etiological proportion outliers that were >5 times greater than the global median cryptosporidiosis etiological proportion, and replacing missing regional cryptosporidiosis etiological proportions with the global median. Furthermore, for children <5 years of age, we proportionally decreased the etiological proportions for all 11 diarrheal pathogens in each region so that the sum of the etiological proportions for all diarrheal pathogens in a region equaled 1. The resultant regional cryptosporidiosis etiological proportions were multiplied by the regional estimates of diarrhea deaths, and the resultant regional cryptosporidiosis mortality was applied to all countries in that WHO region. In the 61 low mortality countries (EUR and other WHO subregion "A" countries), we used a literature review that identified cryptosporidiosis mortality estimates from three countries: Netherlands [[7](#_ENREF_20_7)], New Zealand [[8](#_ENREF_20_8)], and the United States [[10](#_ENREF_20_10)]. These national estimates were based on systematic reviews, national surveillance data, and expert judgment. In these three countries, we used the estimated national cryptosporidiosis mortality (and range) for that country. For low mortality countries without a national estimate, we used the median cryptosporidiosis mortality from the three national studies. The median cryptosporidiosis mortality was from the United States; 0.015 per 100,000 population with range 0.003 – 0.080. |
| AGE DISTRIBUTION: | In middle/high mortality countries, we estimated incidence of cryptosporidiosis separately for children <5 years of age and persons >5 years of age. In low mortality countries, the age distribution for cryptosporidiosis was 16% <5 years, 17% 5-14 years, 13% 15-24 years, 14% 25-34 years, 11% 35-44 years, 9% 45-54 years, 7% 55-64 years, 6% 65-74 years, 7% >75 years [[14](#_ENREF_20_14)]. |
| SEX DISTRIBUTION: | Cryptosporidiosis sex distribution: 50% male. |
| * 11 diarrheal pathogens are: nontyphoidal *Salmonella*, *Campylobacter*, *Shigella*, norovirus, enterotoxigenic *E. coli* (ETEC), enteropathogenic *E. coli* (EPEC), *C*ryptosporidia*, Giardia*, *Entamoeba histolytica*, other diarrheal agents not known to be foodborne (rotavirus and astrovirus), and unspecified agents. | |

Reference

1. Lanata CF, Fischer-Walker CL, Olascoaga AC, Torres CX, Aryee MJ, Black RE, et al. Global causes of diarrheal disease mortality in children <5 years of age: a systematic review. PLOS One. 2013;8:e72788.

2. Walker CL, Rudan I, Liu L, Nair H, Theodoratou E, Bhutta ZA, et al. Global burden of childhood pneumonia and diarrhoea. Lancet. 2013;381:1405-1416.

3. Walker CL, Black RE. Diarrhoea morbidity and mortality in older children, adolescents, and adults. Epidemiol Infect. 2010;138:1215-1226.

4. Fischer Walker CL, Sack D, Black RE. Etiology of diarrhea in older children, adolescents and adults: a systematic review. PLOS Negl Trop Dis. 2010;4:e768.

5. Kirk M, Ford L, Glass K, Hall K. Foodborne illness, Australia, circa 2000 and circa 2010. Emerg Infect Dis. 2014;20:1857-1864.

6. Thomas MK, Murray R, Flockhart L, Pintar K, Pollari F, Fazil A, et al. Estimates of the burden of foodborne illness in Canada for 30 specified pathogens and unspecified agents, circa 2006. Foodborne Pathog Dis. 2013;10:639-648.

7. Havelaar AH, Haagsma JA, Mangen MJ, Kemmeren JM, Verhoef LP, Vijgen SM, et al. Disease burden of foodborne pathogens in the Netherlands, 2009. Int J Food Microbiol. 2012;156:231-238.

8. Cressey P, Lake R. Estimated incidence of foodborne illness in New Zealand: Application of overseas models and multipliers. Report—New Zealand Government, Christ Church, New Zealand. 2011.

9. Tam CC, Rodrigues LC, Viviani L, Dodds JP, Evans MR, Hunter PR, et al. Longitudinal study of infectious intestinal disease in the UK (IID2 study): incidence in the community and presenting to general practice. Gut. 2012;61:69-77.

10. Scallan E, Griffin PM, Angulo FJ, Tauxe RV, Hoekstra RM. Foodborne illness acquired in the United States--unspecified agents. Emerg Infect Dis. 2011;17:16-22.

11. Lamberti LM, Fischer Walker CL, Black RE. Systematic review of diarrhea duration and severity in children and adults in low- and middle-income countries. BMC Public Health. 2012;12:276.

12. Salomon JA, Vos T, Hogan DR, Gagnon M, Naghavi M, Mokdad A, et al. Common values in assessing health outcomes from disease and injury: disability weights measurement study for the Global Burden of Disease Study 2010. Lancet. 2012;380:2129-2143.

13. Lozano R, Naghavi M, Foreman K, Lim S, Shibuya K, Aboyans V, et al. Global and regional mortality from 235 causes of death for 20 age groups in 1990 and 2010: a systematic analysis for the Global Burden of Disease Study 2010. Lancet. 2012;380:2095-2128.

14. Yoder JS, Wallace RM, Collier SA, Beach MJ, Hlavsa MC, Centers for Disease C, et al. Cryptosporidiosis surveillance--United States, 2009-2010. MMWR Surveillance Summary. 2012;61:1-12.

1. ***Giardia* infection**

| INCIDENCE: | The incidence of *Giardia* infection was estimated separately for middle/high mortality countries, and low mortality countries. For the 133 middle/high mortality countries, we used a modification of the Child Health Epidemiology Reference Group (CHERG) approach [[1](#_ENREF_21_1)]. To derive "envelopes" of diarrhea cases, for children <5 years of age we used estimates of diarrhea incidence from a CHERG systematic review [[2](#_ENREF_21_2)] and for persons >5 years of age we used a FERG-commissioned systematic review [[3](#_ENREF_21_3)]. We then estimated the etiological proportions of diarrheal illnesses due to *Giardia and the 10 other diarrheal pathogens** in children <5 years of age using a CHERG and FERG systematic review of etiology studies among outpatients and persons in the community, and the etiological proportion of diarrheal illnesses due to *Giardia* and the 10 other diarrheal pathogens in persons >5 years of age using an updated FERG systematic review of etiology studies among inpatients, outpatients and persons in the community [[4](#_ENREF_21_4)]. The *Giardia* etiological proportions were extracted from studies, and regional median *Giardia* etiological proportions calculated. We modified the CHERG approach by dropping regional median *Giardia* etiological proportion outliers that were >5 times greater than the global median giardiasis etiological proportion, and replacing missing regional giardiasis etiological proportions with the global median. Furthermore, for children <5 years of age, we proportionally decreased the etiological proportions for all 11 diarrheal pathogens in each region so that the sum of the etiological proportions for all diarrheal pathogens in a region equaled 1. The resultant regional giardiasis etiological proportions were multiplied by the regional estimates of diarrhea incidence, and the resultant regional giardiasis incidence was applied to all countries in that WHO region.  In the 61 low mortality countries (EUR and other WHO subregion "A" countries), we used a literature review that identified national incidence estimates for *Giardia* infection from six countries: Australia [[5](#_ENREF_21_5)], Canada [[6](#_ENREF_21_6)], Netherlands [[7](#_ENREF_21_7)], New Zealand [[8](#_ENREF_21_8)], United Kingdom [[9](#_ENREF_21_9)], and the United States [[10](#_ENREF_21_10)]. These national estimates were based on systematic reviews, national surveillance data, and expert judgment. In these six countries, we used the estimated national *Giardia* infection incidence (and range) for that country. For low mortality countries without a national estimate, we used the median incidence from the six national studies. The median incidence was the mean from Canada (which was increased by 8% to account for travelers using proxy information from the United States) and the United States, which was 384.6 per 100,000 population with range 266.4 – 537.0. |
| --- | --- |
| CLINICAL OUTCOMES: | Clinical outcomes were acute *Giardia* infection diarrhea (severe), acute *Giardia* infection diarrhea (moderate), acute *Giardia* infection diarrhea (mild), and *Giardia* infection death. We assumed that 0.5% of *Giardia* cases resulted in severe diarrhea, 8.5% of *Giardia* cases resulted in moderate diarrhea, and 91% of *Giardia* cases resulted in mild diarrhea. |
| DURATION: | In children <5 years of age, duration of severe diarrhea was 8.4 days, moderate diarrhea was 6.4 days, and mild diarrhea was 4.3 days [[11](#_ENREF_21_11)]. Based on the assumed distribution of severe, moderate and mild diarrhea cases, the duration of *Giardia* diarrhea cases in children <5 years of age was estimated to be 4.9 days (minimum 4.3 days - maximum 8.4 days). In persons >5 years of age, the duration of diarrhea was 2.8 days [[11](#_ENREF_21_11)]. |
| DISABILITY WEIGHT: | Acute *Giardia* diarrhea (severe): GBD 2010 disability weight of 0.281 (95% CI 0.184-0.399) for diarrhea, severe. Acute *Giardia* diarrhea (moderate): GBD 2010 disability weight of 0.202 (95% CI 0.133-0.299) for diarrhea, moderate. Acute *Giardia* diarrhea (mild): GBD 2010 disability weight of 0.061 (95% CI 0.036-0.093) for diarrhea, mild [[12](#_ENREF_21_12)]. |
| MORTALITY: | We estimated no *Giardia* deaths. |
| AGE DISTRIBUTION: | In middle/high mortality countries, we estimated incidence of *Giardia* infections separately for children <5 years of age and persons >5 years of age. In low mortality countries, the age distribution for *Giardia* was 20% <5 years, 17% 5-14 years, 10% 15-24 years, 11% 25-34 years, 12% 35-44 years, 12% 45-54 years, 9% 55-64 years, 5% 65-74 years, 4% >75 years [[13](#_ENREF_21_13)]. |
| SEX DISTRIBUTION: | *Giardia* infection sex distribution: 50% male. |
| * 11 diarrheal pathogens are: nontyphoidal *Salmonella*, *Campylobacter*, *Shigella*, norovirus, enterotoxigenic *E. coli* (ETEC), enteropathogenic *E. coli* (EPEC), *C*ryptosporidia*, Giardia*, *Entamoeba histolytica*, other diarrheal agents not known to be foodborne (rotavirus and astrovirus), and unspecified agents. | |

Reference

1. Lanata CF, Fischer-Walker CL, Olascoaga AC, Torres CX, Aryee MJ, Black RE, et al. Global causes of diarrheal disease mortality in children <5 years of age: a systematic review. PLOS One. 2013;8:e72788.

2. Walker CL, Rudan I, Liu L, Nair H, Theodoratou E, Bhutta ZA, et al. Global burden of childhood pneumonia and diarrhoea. Lancet. 2013;381:1405-1416.

3. Walker CL, Black RE. Diarrhoea morbidity and mortality in older children, adolescents, and adults. Epidemiol Infect. 2010;138:1215-1226.

4. Fischer Walker CL, Sack D, Black RE. Etiology of diarrhea in older children, adolescents and adults: a systematic review. PLOS Negl Trop Dis. 2010;4:e768.

5. Kirk M, Ford L, Glass K, Hall K. Foodborne illness, Australia, circa 2000 and circa 2010. Emerg Infect Dis. 2014;20:1857-1864.

6. Thomas MK, Murray R, Flockhart L, Pintar K, Pollari F, Fazil A, et al. Estimates of the burden of foodborne illness in Canada for 30 specified pathogens and unspecified agents, circa 2006. Foodborne Pathog Dis. 2013;10:639-648.

7. Havelaar AH, Haagsma JA, Mangen MJ, Kemmeren JM, Verhoef LP, Vijgen SM, et al. Disease burden of foodborne pathogens in the Netherlands, 2009. Int J Food Microbiol. 2012;156:231-238.

8. Cressey P, Lake R. Estimated incidence of foodborne illness in New Zealand: Application of overseas models and multipliers. Report—New Zealand Government, Christ Church, New Zealand. 2011.

9. Tam CC, Rodrigues LC, Viviani L, Dodds JP, Evans MR, Hunter PR, et al. Longitudinal study of infectious intestinal disease in the UK (IID2 study): incidence in the community and presenting to general practice. Gut. 2012;61:69-77.

10. Scallan E, Griffin PM, Angulo FJ, Tauxe RV, Hoekstra RM. Foodborne illness acquired in the United States--unspecified agents. Emerg Infect Dis. 2011;17:16-22.

11. Lamberti LM, Fischer Walker CL, Black RE. Systematic review of diarrhea duration and severity in children and adults in low- and middle-income countries. BMC Public Health. 2012;12:276.

12. Salomon JA, Vos T, Hogan DR, Gagnon M, Naghavi M, Mokdad A, et al. Common values in assessing health outcomes from disease and injury: disability weights measurement study for the Global Burden of Disease Study 2010. Lancet. 2012;380:2129-2143.

13. Yoder JS, Gargano JW, Wallace RM, Beach MJ. Giardiasis surveillance--United States, 2009-2010. MMWR Surveill Summ. 2012;61:13-23.

1. **Amoebiasis**

| INCIDENCE: | The incidence of diarrhea due to amoebiasis was estimated separately for middle/high mortality countries, and low mortality countries. For the 133 middle/high mortality countries, we used a modification of the Child Health Epidemiology Reference Group (CHERG) approach [[1](#_ENREF_22_1)]. To derive "envelopes" of diarrhea cases, for children <5 years of age we used estimates of diarrhea incidence from a CHERG systematic review [[2](#_ENREF_22_2)] and for persons >5 years of age we used a FERG-commissioned systematic review [[3](#_ENREF_22_3)]. We then estimated the etiological proportions of diarrheal illnesses due to *Entamoeba histolytica and the 10 other diarrheal pathogens** in children <5 years of age using a CHERG and FERG systematic review of etiology studies among outpatients and persons in the community, and the etiological proportion of diarrheal illnesses due to *Entamoeba histolytica* and the 10 other diarrheal pathogens in persons >5 years of age using an updated FERG systematic review of etiology studies among inpatients, outpatients and persons in the community [[4](#_ENREF_22_4)]. The amoebiasis etiological proportions were extracted from studies, and regional median amoebiasis etiological proportions calculated. We modified the CHERG approach by dropping regional median amoebiasis etiological proportion outliers that were >5 times greater than the global median amoebiasis etiological proportion, and replacing missing regional amoebiasis etiological proportions with the global median. Furthermore, for children <5 years of age, we proportionally decreased the etiological proportions for all 11 diarrheal pathogens in each region so that the sum of the etiological proportions for all diarrheal pathogens in a region equaled 1. The resultant regional amoebiasis etiological proportions were multiplied by the regional estimates of diarrhea incidence, and the resultant regional amoebiasis incidence was applied to all countries in that WHO region. We estimated no amoebiasis cases in the 61 low mortality countries (EUR and other WHO subregion "A" countries). |
| --- | --- |
| CLINICAL OUTCOMES: | Clinical outcomes were acute amoebiasis diarrhea (severe), acute amoebiasis diarrhea (moderate), acute amoebiasis diarrhea (mild), and amoebiasis death. We assumed that 0.5% of amoebiasis cases resulted in severe diarrhea, 8.5% of amoebiasis cases resulted in moderate diarrhea, and 91% of amoebiasis cases resulted in mild diarrhea. |
| DURATION: | In children <5 years of age, duration of severe diarrhea was 8.4 days, moderate diarrhea was 6.4 days, and mild diarrhea was 4.3 days [[5](#_ENREF_22_5)]. Based on the assumed distribution of severe, moderate and mild diarrhea cases, the duration of amoebiasis diarrhea cases in children <5 years of age was estimated to be 4.9 days (minimum 4.3 days - maximum 8.4 days). In persons >5 years of age, the duration of diarrhea was 2.8 days [[5](#_ENREF_22_5)]. |
| DISABILITY WEIGHT: | Acute amoebiasis diarrhea (severe): GBD 2010 disability weight of 0.281 (95% CI 0.184-0.399) for diarrhea, severe. Acute amoebiasis diarrhea (moderate): GBD 2010 disability weight of 0.202 (95% CI 0.133-0.299) for diarrhea, moderate. Acute amoebiasis diarrhea (mild): GBD 2010 disability weight of 0.061 (95% CI 0.036-0.093) for diarrhea, mild [[6](#_ENREF_22_6)]. |
| MORTALITY: | The mortality of amoebiasis was estimated separately for middle/high mortality countries, and low mortality countries. For the 133 middle/high mortality countries, we used a modification of the CHERG approach [[1](#_ENREF_22_1)]. We received envelopes of diarrheal deaths from WHO; because this estimate was not available with an uncertainty interval, we used the uncertainty range from the GBD 2010 estimate of diarrheal deaths (81.7% to 114.6% around the point estimate) [[7](#_ENREF_22_7)]. We then estimated the etiological proportions of diarrheal deaths due to *Entamoeba histolytica* and the 10 other diarrheal pathogens* in children <5 years of age using a CHERG and FERG systematic review of etiology studies among inpatients, and the etiological proportions of diarrheal deaths due to *Entamoeba histolytica* and the 10 other diarrheal pathogens in persons >5 years of age using an updated FERG systematic tic review of etiology studies among inpatients [[4](#_ENREF_22_4)]. The amoebiasis etiological proportions were extracted from studies, and regional median amoebiasis etiological proportions calculated. We modified the CHERG approach by dropping regional median amoebiasis etiological proportion outliers that were >5 times greater than the global median amoebiasis etiological proportion, and replacing missing regional amoebiasis etiological proportions with the global median. Furthermore, for children <5 years of age, we proportionally decreased the etiological proportions for all 11 diarrheal pathogens in each region so that the sum of the etiological proportions for all diarrheal pathogens in a region equaled 1. The resultant amoebiasis etiological proportions were multiplied by the regional estimates of diarrhea deaths, and the resultant regional amoebiasis mortality was applied to all countries in that WHO region. We estimated no amoebiasis deaths in the 61 low mortality countries (EUR and other WHO subregion "A" countries). |
| AGE DISTRIBUTION: | Estimated incidence of amoebiasis diarrhea separately for children <5 years of age and persons >5 years of age. No other information on age distribution for diarrhea cases. |
| SEX DISTRIBUTION: | Amoebiasis sex distribution: 50% male. |
| * 11 diarrheal pathogens are: nontyphoidal *Salmonella*, *Campylobacter*, *Shigella*, norovirus, enterotoxigenic *E. coli* (ETEC), enteropathogenic *E. coli* (EPEC), *C*ryptosporidia*, Giardia*, *Entamoeba histolytica*, other diarrheal agents not known to be foodborne (rotavirus and astrovirus), and unspecified agents. | |

Reference

1. Lanata CF, Fischer-Walker CL, Olascoaga AC, Torres CX, Aryee MJ, Black RE, et al. Global causes of diarrheal disease mortality in children <5 years of age: a systematic review. PLOS One. 2013;8:e72788.

2. Walker CL, Rudan I, Liu L, Nair H, Theodoratou E, Bhutta ZA, et al. Global burden of childhood pneumonia and diarrhoea. Lancet. 2013;381:1405-1416.

3. Walker CL, Black RE. Diarrhoea morbidity and mortality in older children, adolescents, and adults. Epidemiol Infect. 2010;138:1215-1226.

4. Fischer Walker CL, Sack D, Black RE. Etiology of diarrhea in older children, adolescents and adults: a systematic review. PLOS Negl Trop Dis. 2010;4:e768.

5. Lamberti LM, Fischer Walker CL, Black RE. Systematic review of diarrhea duration and severity in children and adults in low- and middle-income countries. BMC Public Health. 2012;12:276.

6. Salomon JA, Vos T, Hogan DR, Gagnon M, Naghavi M, Mokdad A, et al. Common values in assessing health outcomes from disease and injury: disability weights measurement study for the Global Burden of Disease Study 2010. Lancet. 2012;380:2129-2143.

7. Lozano R, Naghavi M, Foreman K, Lim S, Shibuya K, Aboyans V, et al. Global and regional mortality from 235 causes of death for 20 age groups in 1990 and 2010: a systematic analysis for the Global Burden of Disease Study 2010. Lancet. 2012;380:2095-2128.
